# Linker‐Locking Strategy for the Rigidification of Flexible Metal–Organic Framework: Structural Stabilization and Controlled ESIPT for H_2_O/D_2_O Sensing

**DOI:** 10.1002/advs.77856

**Published:** 2026-09-27

**Authors:** Amitosh Sharma, Seonghwan Lee, Seo I Park, Hak‐Won Nho, Junmo Seong, Eunseo Lee, Oh‐Hoon Kwon, Myoung Soo Lah, Jaewoong Lim

**Affiliations:** ^1^ Department of Chemistry Ulsan National Institute of Science and Technology (UNIST) Ulsan Republic of Korea; ^2^ Department of Science Education Ewha Womans University Seoul Republic of Korea; ^3^ Institute For Multiscale Matter and System (IMMS) Ewha Womans University Seoul Republic of Korea; ^4^ Department of Chemistry Korea Advanced Institute of Science and Technology (KAIST) Daejeon Republic of Korea

**Keywords:** ESIPT control, flexible metal–organic frameworks, H_2_O/D_2_O sensing, linker‐locking, structural stabilization

## Abstract

Controlling the photodynamics of excited‐state intramolecular proton transfer (ESIPT) fluorophores is crucial for sensing, due to their large Stokes shifts and dual emission. However, structural flexibility and solvent‐induced degradation often hinder isolating a single tautomeric emission in water. Here, we introduce a *linker‐locking strategy* that rigidifies the flexible metal–organic framework (MOF) MIL‐53‐(OH)_2_ through cooperative stabilization by encapsulated ligands and structural water, forming a stable phase, **rigid L@MIL‐53‐(OH)_2_**. The hydrogen‐bonding network among structural water, encapsulated ligands, and the framework stabilizes the pore structure while preserving reversible interconversion between flexible and rigid phases via ligand and water removal or post‐encapsulation. This rigidification suppresses particle fragmentation in aqueous media and enables controlled ESIPT behavior. While flexible MIL‐53‐(OH)_2_ exhibits mixed enol and keto emissions due to size‐dependent heterogeneity, **rigid L@MIL‐53‐(OH)_2_** displays a single keto emission with prolonged excited‐state lifetimes (>14 ns in D_2_O vs <5 ns in H_2_O) and distinct optical responses in H_2_O and D_2_O. Consequently, the pronounced kinetic isotope effect allows **rigid L@MIL‐53‐(OH)_2_** to sensitively detect trace H_2_O in D_2_O with high reusability over at least five cycles. This work establishes *linker locking* as a new strategy for stabilizing flexible MOFs and controlling excited‐state processes for aqueous sensing.

## Graphical abstract

A linker‐locking strategy rigidifies the flexible metal–organic framework (MOF) MIL‐53‐(OH)_2_ via the cooperative encapsulation of ligands and structural water. This network significantly enhances the framework's aqueous stability. Crucially, rigidification suppresses enol emission to yield a single keto tautomer state with an extended lifetime, enabling sensitive, reusable H_2_O/D_2_O isotope sensing.

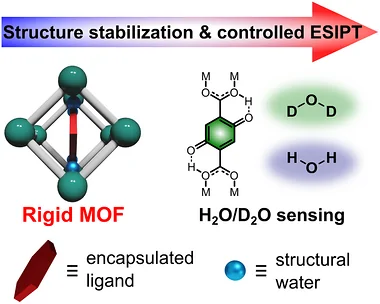

## 1 Introduction

The growing demand for high‐purity materials across multiple scales has intensified the need for sensitive and reliable methods to detect trace impurities in energy and environmental systems [1, 2, 3, 4, 5]. Luminescence‐based detection has emerged as a powerful analytical approach due to its rapid response and exceptional sensitivity, enabling the identification of analytes through optical signals with low detection limits [2, 6, 7, 8]. Organic fluorophores are particularly attractive because their emission properties can be selectively modulated through *π–π* stacking, hydrogen bonding, and electrostatic interactions [9, 10, 11].

Among multi‐emissive organic systems, excited‐state intramolecular proton transfer (ESIPT) represents a powerful photochemical mechanism characterized by ultrafast enol‐keto phototautomerism [12, 13, 14, 15]. Because ESIPT is highly sensitive to hydrogen‐bonding strength, it serves as an optical probe for probing solvent composition, including isotope effects in deuterium‐containing media [12, 13, 14, 15]. For example, trace H_2_O impurities in high‐purity heavy water (D_2_O), a critical material for nuclear reactors and a widely used solvent for synthesizing deuterated organic compounds, can be detected through the fluorescence of organic chromophores bearing proton‐exchangeable functional groups, such as hydroxyl moieties [16, 17, 18, 19, 20]. When the p*K*_a_ of the proton‐exchangeable site is properly matched to the acidity difference between H_2_O (pH 7.0) and D_2_O (pD 7.5) at 25°C, distinct fluorescence responses emerge [16, 17, 18].

For practical sensing applications, materials must be not only sensitive but also chemically stable, reusable, and resistant to degradation under operating conditions [21, 22, 23, 24, 25]. In this regard, metal–organic frameworks (MOFs) have emerged as promising sensing platforms owing to their high chemical and thermal stability, as well as superior long‐term stability, rendering them well suited for heterogeneous sensing [26, 27, 28, 29, 30, 31, 32, 33, 34, 35]. Importantly, the spatial isolation of organic linkers within the frameworks, i.e., *solid‐state fluorophores*, prevents fluorophore aggregation, which typically diminishes emission in the solid state through *π–π* interactions and intermolecular hydrogen bonding [6, 9, 36, 37, 38, 39, 40]. For example, while perylene‐based organic fluorophores do not exhibit detectable emission in the solid state, the corresponding isolated perylene‐based MOF demonstrates strong luminescence for oxygen sensing due to the suppression of intermolecular interactions (*e.g., π–π* stacking) [40]. Furthermore, functional groups incorporated into MOF linkers enable the detection of diverse chemical stimuli, including hazardous substances, solvents, and volatile organic compounds (VOCs), as well as physical stimuli such as temperature, pH, and humidity [41, 42, 43, 44]. ESIPT functionality can be introduced by incorporating, for example, dihydroxyl groups, enabling the sensing of acid vapors (e.g., HCl) through enol‐form emission and base vapors (e.g., triethylamine) through keto‐form emission [45]. However, these systems typically exhibit enhanced emission from either the enol or keto form rather than a well‐defined, isolated single emission band. Consequently, although reported ESIPT‐active MOFs often favor one tautomer, achieving precise control over a single, well‐resolved emission state within the framework remains a significant challenge.

To achieve selective sensing of specific environmental targets, flexible MOFs can undergo reversible structural transformations in response to internal guests, such as solvents or gas molecules, or external stimuli, like temperature, pressure, and light [46, 47, 48, 49, 50, 51, 52, 53]. Functional groups on organic linkers, such as amine and hydroxyl moieties, provide chemically selective interaction sites, while framework flexibility can amplify sensing responses (e.g., ESIPT) through cooperative host‐guest interactions [54, 55, 56]. Among flexible MOFs, the MIL‐53 family is particularly well known for its structural robustness and versatility in linker functionalization (H_2_BDC‐X, where H_2_BDC = 1,4‐benzenedicarboxylic acid and X = NH_2_, OH, Br, COOH, and (OH)_2_) [57, 58, 59, 60, 61, 62, 63]. Depending on the synthesis conditions, additional organic ligands can be encapsulated within the pores of MIL‐53‐X, and their removal is typically required to induce breathing effects [60, 64, 65]. In contrast, as‐synthesized MIL‐53‐X prepared in aqueous media inherently contains free ligands within pore spaces, however, this feature has not been systematically explored.

MIL‐53‐(OH)_2_ is especially attractive for ESIPT‐based sensing because its hydroxyl groups provide hydrogen‐bonding sites and potential coordination sites for metal cations, making it a promising candidate for sensing N_2_H_4_, nitroaromatic compounds, and temperature [63, 66, 67]. Nevertheless, its ESIPT response remains difficult to control, particularly with respect to the relative contribution and relaxation dynamics of the enol (short‐lived) and keto (long‐lived) emissive states, as the framework does not selectively stabilize either tautomer in response to specific analytes.

**SCHEME 1:**
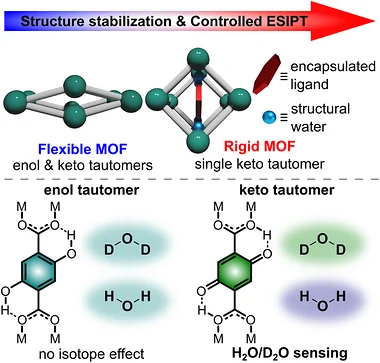
Schematic representation of the *linker‐locking* strategy. (*top*) Transition from a flexible MOF to a rigid phase via encapsulation of ligands and structural water, enabling structural stabilization and ESIPT control. (*bottom*) Proposed ESIPT mechanism highlighting the isotope‐independent enol tautomer and the pronounced H_2_O/D_2_O sensitivity of the keto tautomer mediated by hydrogen‐bonding network.

**FIGURE 1:**
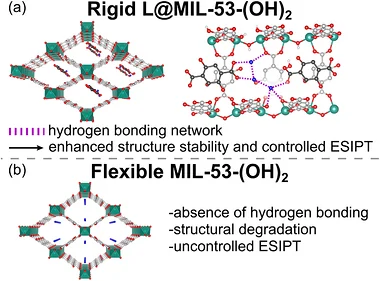
Crystal structures of (a) **rigid L@MIL‐53‐(OH)_2_** and (b) flexible MIL‐53‐(OH)_2_. Dashed lines represent hydrogen‐bonding interactions between the framework, encapsulated ligands, and structural water. Gray and red spheres, and green spheres and polyhedra, represent carbon, oxygen, and indium atoms in the framework, respectively. Dark gray spheres represent carbon atoms in the encapsulated ligand, and blue spheres represent oxygen atoms for structural water. Hydrogen atoms are omitted for clarity.

Herein, we report a *linker‐locking strategy* that rigidifies MIL‐53‐(OH)_2_ through the retention of residual ligands originally formed during synthesis, together with hydrogen‐bonded structural water, thereby enabling precise control over ESIPT behavior with enhanced structural stability (Scheme 1 and Figure 1). The encapsulation of these ligands, in conjunction with hydrogen‐bonded structural water and framework linkers, suppresses the intrinsic breathing behavior of MIL‐53‐(OH)_2_, stabilizes the pore environment, and enables prolonged excited‐state relaxation time, ultimately converting a mixed enol/keto emission into a single keto‐form emission (Scheme 1).

In addition, we demonstrate that flexible MIL‐53‐(OH)_2_ undergoes pronounced topochemical‐driven fragmentation upon exposure to water, resulting in crystal fragmentation into micro‐ and nanoscale particles. These fragments exhibit distinct ESIPT characteristics, adsorption behavior, and fluorescence lifetimes. This size‐dependent heterogeneity, coupled with the absence of a well‐defined hydrogen‐bonding interface between water and the framework hydroxyl groups, leads to the uncontrolled coexistence of enol‐ and keto‐emissive species, which precludes the reliable discrimination between H_2_O and D_2_O.

In contrast, the *linker‐locked* rigid phase (**rigid L@MIL‐53‐(OH)_2_**) containing 0.6 equivalents of encapsulated ligand and 2.0 equivalents of hydrogen‐bonded structural water, exhibits suppressed framework flexibility, enhanced water stability, and a predictable fluorescence response governed by keto‐state emission. We first demonstrate that the rigid and flexible phases are reversibly interconverted: removal of the encapsulated ligands and structural water by polar solvents restores the flexible framework, whereas the reintroduction of ligands in water at elevated temperatures regenerates the rigid structure. This behavior highlights the reversible nature of the rigidification process and the cooperative role of both components.

We further establish that keto‐emission in **rigid L@MIL‐53‐(OH)_2_** is the result of the encapsulated ligand confined within the rigid framework, where structure rigidification stabilizes the excited keto state through strengthened hydrogen‐bonding interactions. This stabilization leads to prolonged excited‐state relaxation, which enables discrimination between H_2_O and D_2_O based on proton/deuteron isotope effects and differences in hydrogen‐bonding strength. Consequently, the fluorescence intensity of **rigid L@MIL‐53‐(OH)_2_** varies systematically with the H_2_O/D_2_O ratio, enabling sensitive detection of trace H_2_O in D_2_O with a detection limit of 0.377% and high reusability over at least five cycles, highlighting its potential as a robust heterogeneous sensing platform.

## 2 Results and Discussion

MIL‐53‐(OH)_2_ was selected to investigate the effects of framework flexibility on fluorescence, particularly ESIPT behavior, for several reasons: (i) its hydroxyl groups provide the hydrogen‐bonding sites necessary for ESIPT; (ii) its emissive MOF structure enables the sensing of guest molecules; (iii) it is among the most stable flexible MOFs known to date, constructed from trivalent metals (M^3+^ = Al, Cr, In, Ga); and (iv) its synthesis can be controlled to yield either ligand‐containing structures or ligand‐free flexible frameworks (Figure 1) [60, 63, 66, 67, 68].

We specifically chose the indium (In)‐based analogue of MIL‐53‐(OH)_2_, which is isostructural with the aluminum‐based congener, because it can be synthesized as micrometer‐sized single crystals suitable for crystallographic analysis. In addition, the synthesis conditions can be tuned to either encapsulate ligands within the pores or exclude them. Furthermore, In‐based MIL‐53 frameworks are known for their high stability due to the strong coordination bonds predicted by hard‐soft acid‐base (HSAB) theory between the indium centers and carboxylate ligands [69, 70, 71].

We first synthesized the *linker‐locked* rigid phase, **rigid L@MIL‐53‐(OH)_2_**, via a hydrothermal reaction of In(NO_3_)_3_∙xH_2_O and 2,5‐dihydroxy‐1,4‐benzenedicarboxylic acid (H_2_BDC‐(OH)_2_) in water, which yielded 10−30 µm‐sized single crystals (Figure 1; Figures S1 and S2; see more details in Supporting Information). The composition of **rigid L@MIL‐53‐(OH)_2_** can be described as [In(OH)(BDC‐(OH)_2_)]·(H_2_BDC‐(OH)_2_)_0.6_·(H2O)_2.0_, where L represents the encapsulated H_2_BDC‐(OH)_2_ ligand. Single‐crystal X‐ray diffraction (SCXRD), thermogravimetric analysis (TGA), and elemental analysis (EA) confirmed that **rigid L@MIL‐53‐(OH)_2_** contains 0.6 equivalents of encapsulated H_2_BDC‐(OH)_2_ ligands and 2.0 equivalents of structural H_2_O within the framework pores (Figure 1; Figures S1 and S2). Both the encapsulated ligands and structural water molecules form hydrogen‐bonding interactions with the framework, which helps in maintaining crystallinity, as evidenced by powder X‐ray diffraction (PXRD) patterns even after air drying (Figure 1).

**FIGURE 2:**
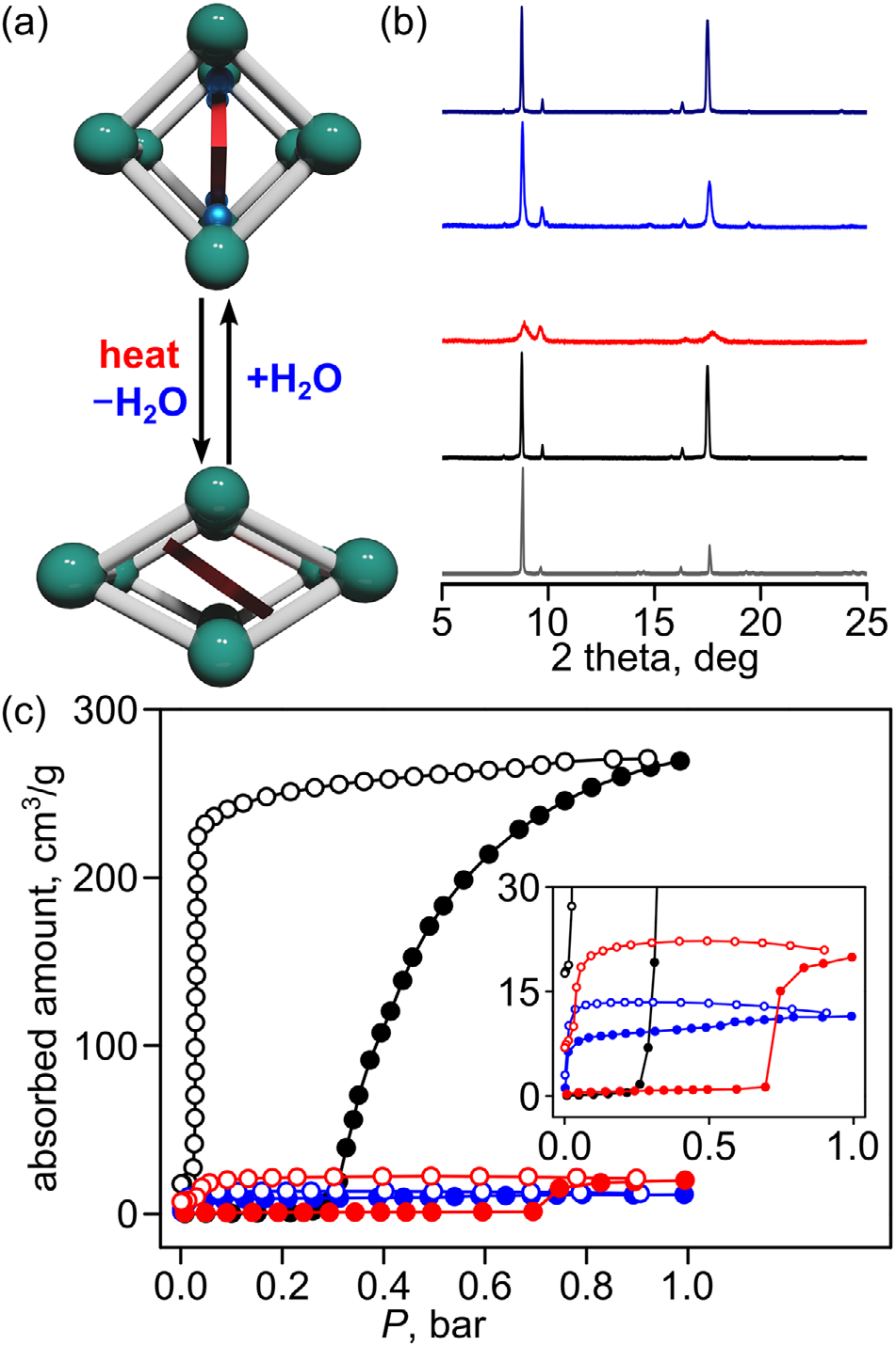
(a) Schematic of the reversible integration and removal of structural water in **rigid L@MIL‐53‐(OH)_2_**, highlighting the transition between open and collapsed pore states. (b) PXRD patterns of **rigid L@MIL‐53‐(OH)_2_**: simulated (gray), as‐synthesized (black), after heating at 150°C for one hour (red), exposure to 98% RH for one hour (blue), and resoaking in H_2_O (dark blue). (c) CO_2_ sorption isotherms of flexible MIL‐53‐(OH)_2_ (black), **L@MIL‐53‐(OH)_2_‐RTa** (red), and **L@MIL‐53‐(OH)_2_‐150a** (blue) at 195 K. Filled circles represent adsorption and empty circles represent desorption.

Notably, the structural water molecules form strong hydrogen bonds with the hydroxyl groups of the framework and the carboxylic acid groups of the encapsulated ligand, generating an extended hydrogen bonding network that further stabilizes the structure (Figure 1; Tables S4 and S5). To remove hydrogen‐bonded structural water, **rigid L@MIL‐53‐(OH)_2_** was heated at 150°C for one hour, resulting in reduced water content and deterioration of crystallinity (Figure 2). Remarkably, the crystallinity was restored upon exposure to 98% relative humidity (RH) or by soaking in water, demonstrating that hydrogen‐bonded structural water plays a critical role in stabilizing the framework (Figure 2).

In comparison, flexible MIL‐53‐(OH)_2_ was obtained under modified synthesis conditions via solvothermal reaction of In(NO_3_)_3_∙xH_2_O and H_2_BDC‐(OH)_2_ in a H_2_O/ *N*,*N*‐dimethylformamide (DMF) mixed solvent, yielding 20−50 µm‐sized single crystals of MIL‐53‐(OH)_2_ (In(OH)(BDC‐(OH)_2_)) (Figure 1 and see more details in Supporting Information) [63]. In this case, only solvent molecules occupy the pore space without forming strong hydrogen‐bonding interactions with the framework, in contrast to **rigid L@MIL‐53‐(OH)_2_**, which contains strongly hydrogen‐bonded solvent molecules associated with both the framework and encapsulated ligands. As a result, these solvent molecules are readily exchanged with other solvents and can be removed by heating. Accordingly, flexible MIL‐53‐(OH)_2_ exhibited different PXRD patterns depending on the solvent environment and lost crystallinity upon solvent removal by air‐drying or heating (Figures 1 and S3). Surprisingly, upon resoaking the dried MIL‐53‐(OH)_2_ in water, the crystals partially decomposed into micrometer‐sized particles, while the overall crystallinity was retained. Additionally, the filtrate contained 20−30 nm‐sized nanoparticles generated during the decomposition process (Figures S3–S5).

Next, to correlate structural rigidity and flexibility with adsorption behavior, we investigated the porosity of flexible MIL‐53‐(OH)_2_ and **rigid L@MIL‐53‐(OH)_2_** with particular emphasis on the role of structural water in the framework rigidity, using gas adsorption experiments, as MIL‐53‐(OH)_2_ is a known flexible framework (Figure 2; Figures S6 and S7) [63, 72]. Flexible MIL‐53‐(OH)_2_ was activated by heating at 120°C following a reported procedure [63]. To evaluate the role of structural water, **rigid L@MIL‐53‐(OH)_2_** was activated under two conditions: at room temperature (RT), retaining structural water (denoted **L@MIL‐53‐(OH)_2_‐RTa**), and at 150°C, removing structural water (denoted **L@MIL‐53‐(OH)_2_‐150a**).

We first confirmed that the pore apertures of all frameworks, **L@MIL‐53‐(OH)_2_‐RTa, L@MIL‐53‐(OH)_2_‐150a**, and flexible MIL‐53‐(OH)_2_ are inaccessible to N_2_ (kinetic diameter: 3.6 Å) [73, 74] as evidenced by exhibited non‐porous behavior toward N_2_ adsorption isotherms at 77 K (Figure S6). To further evaluate framework porosity (CO_2_ kinetic diameter: 3.3 Å) and flexibility (i.e., *gate‐opening* behavior), CO_2_ adsorption measurements were conducted at 195 K [73, 74]. Flexible MIL‐53‐(OH)_2_ exhibited characteristic breathing behavior during CO_2_ adsorption‐desorption, consistent with previous reports [63]. Pore opening occurred at 0.26 bar, reaching a maximum CO_2_ uptake of 269 cm^3^/g at 1.00 bar, followed by hysteretic desorption with pore closure at 0.03 bar (Figure 2).

In contrast, the CO_2_ adsorption behavior of **rigid L@MIL‐53‐(OH)_2_** depended strongly on the presence of structural water. **L@MIL‐53‐(OH)_2_‐RTa** exhibited type I adsorption with a maximum uptake of 12 cm^3^/g at 1.00 bar, but **L@MIL‐53‐(OH)_2_‐150a** displayed gate‐opening behavior, with pore opening at 0.69 bar and a maximum uptake of 20 cm^3^/g, followed by hysteric desorption with pore closing at 0.05 bar (Figure 2). These results suggest that the rigid framework containing both encapsulated ligands and structural water maintains a narrow pore environment that allows limited CO_2_ adsorption without structural transition. Upon removal of structural water, which participates in extensive hydrogen‐bonding interactions with both the framework and the encapsulated ligands, the framework adopts a closed‐pore state that can undergo gate‐opening response to CO_2_ at 195 K but remains inaccessible to N_2_.

These findings highlight the critical role of structural water in modulating the flexibility of **rigid L@MIL‐53‐(OH)_2_**. To further investigate this effect, H_2_O vapor adsorption experiments were conducted to evaluate the hydrophilicity arising from structural water within the framework [75, 76]. **L@MIL‐53‐(OH)_2_‐RTa** exhibited pronounced hydrophilic behavior, as evidenced by significant H_2_O uptake at low relative pressure (0.1 bar, 68 cm^3^/g) attributed to hydrogen‐bonding interactions between structural water and adsorbed H_2_O molecules (Figure S7). In contrast, the collapsed frameworks (**L@MIL‐53‐(OH)_2_‐150a** and flexible MIL‐53‐(OH)_2_) displayed nearly linear H_2_O adsorption isotherms, consistent with gradual pore opening induced by the interactions with water molecules (Figure S7). These results indicate that the coexistence of structural water and encapsulated ligands creates a hydrophilic pore environment that significantly influences adsorption behavior.

We next examined the reversibility of ligand encapsulation. To evaluate encapsulated ligand removal, **rigid L@MIL‐53‐(OH)_2_** was soaked in various solvents, including a protic polar solvent (methanol, MeOH), aprotic polar solvents (acetone and DMF), and nonpolar solvents (hexane and toluene), for periods ranging from one day to two weeks, either at room temperature or at elevated temperatures (100°C–120°C).

**FIGURE 3:**
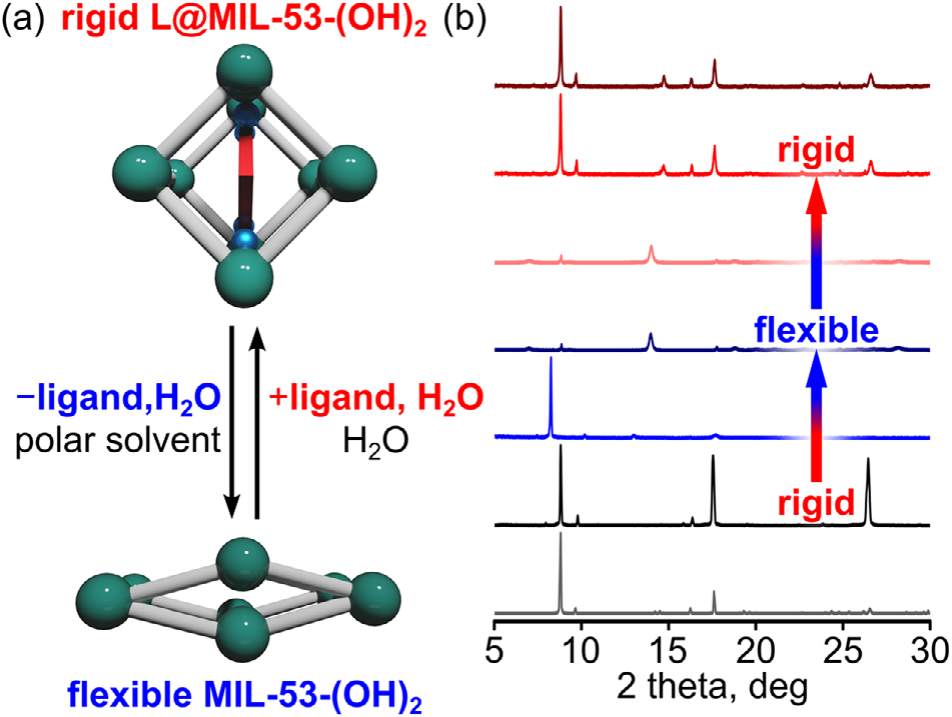
(a) Schematic of reversibility of **rigid L@MIL‐53‐(OH)_2_** and flexible MIL‐53‐(OH)_2_ by soaking in protic solvents and post‐encapsulation. (b) PXRD patterns of simulated (gray), as‐synthesized **rigid L@MIL‐53‐(OH)_2_** (black), **rigid L@MIL‐53‐(OH)_2_** soaked in MeOH for one day at RT as‐synthesized (blue) and after room‐temperature drying (dark blue), dried flexible MIL‐53‐(OH)_2_ (bright red), post‐encapsulation of flexible MIL‐53‐(OH)_2_ as‐synthesized (red), and after room temperature drying (dark red).

In methanol, a protic polar solvent, the encapsulated ligands were readily removed from the framework, as confirmed by the detection of dissolved H_2_BDC‐(OH)_2_ using ^1^H nuclear magnetic resonance (NMR) spectroscopy (CD_3_OD) and by TGA, which gave a formula of [In(OH)(BDC‐(OH)_2_)]·(H_2_BDC‐(OH)_2_)_0.18_·(H_2_O)_1.0_, indicating substantial loss of encapsulated ligands and structural water (Figures S8 and S9). Following removal of both encapsulated ligands and structural water, the framework lost its rigidity and converted to a phase similar to flexible MIL‐53‐(OH)_2_ soaked in MeOH (Figures 3 and S10). Upon drying, the methanol‐treated sample further transformed into a characteristic dried flexible phase (Figures 3 and S10). Consistent with this structural transformation, the framework exhibited flexible behavior, including gate‐opening behavior in the CO_2_ adsorption isotherm at 195 K (Figure S11).

In aprotic polar solvents, acetone and DMF, removal of encapsulated ligands and structural water also occurred, but at a significantly slower rate compared to a protic polar solvent (methanol), requiring one week in acetone and over one month in DMF at room temperature (Figures S12 and S13). However, upon heating to 120°C, the ligand dissociation kinetics were markedly enhanced, and encapsulated ligands were effectively removed in DMF within one day, and the framework exhibited flexible behavior (Figures S12 and S13). These results indicate that polar solvents capable of dissolving the encapsulated ligand (H_2_BDC‐(OH)_2_), promote structural transformation via removal of both encapsulated ligands and structural water. Notably, protic polar solvents induce this transformation more rapidly than aprotic polar solvents, likely due to their ability to act as hydrogen donors, thereby facilitating disruption of the hydrogen‐bonding network among encapsulated ligands, structural water, and the framework.

Furthermore, nonpolar solvents (hexane and toluene), which are unable to dissolve the encapsulated ligands or structural water, were used to soak **rigid L@MIL‐53‐(OH)_2_**. Under these conditions, no transformation from rigid to the flexible phase was observed at room temperature over several weeks (Figures S14 and S15). At elevated temperature (100°C), the framework maintained its rigid structure in toluene, although a slight decrease in crystallinity was observed after one week (Figure S15). Conversion from the rigid to the flexible phase, via removal of encapsulated ligands and structural water, requires the use of polar solvents capable of dissolving these components.

Next, to investigate the reverse process, encapsulation of ligands into flexible MIL‐53‐(OH)_2_, we hypothesized that water plays a key role as structural water. Following the synthesis‐like conditions for **rigid L@MIL‐53‐(OH)_2_**, flexible MIL‐53‐(OH)_2_ and H_2_BDC‐(OH)_2_ were heated together in water at 100°C for one day (Figure 3 and see more details in Supporting Information). Under these conditions, flexible MIL‐53‐(OH)_2_ was converted into a rigid phase, **L@MIL‐53‐(OH)_2_** as confirmed by PXRD. The experimental pattern matched that of directly synthesized **rigid L@MIL‐53‐(OH)_2_** and showed no change before and after air‐drying, indicating structural rigidity. TGA further revealed the incorporation of additional organic ligands (0.27 equivalents) within the framework, confirming successful encapsulation together with structural water, which cooperatively rigidifies the framework (Figures 3 and S16). In contrast, post‐encapsulation of ligands into flexible MIL‐53‐(OH)_2_ in MeOH, where structural water is absent, did not induce conversion to the rigid phase but instead preserved the flexible phase (Figure S17). The framework retained its flexibility, even though 0.25 equivalents of ligands were incorporated into the framework (Figures S17 and S18).

This reversible encapsulation of ligands and structural water enables controlled interconversion between flexible and rigid phases. Both water and encapsulated ligands are the key to rigidification; their presence is necessary to maintain the rigid phase, and their removal converts the structure back to the flexible phase. Furthermore, maintaining the rigid phase relies on hydroxyl groups within the framework, which additionally provides hydrogen‐bonding sites with distinct p*K*_a_ values, offering an opportunity to discriminate between H_2_O and D_2_O based on their different acidities (p*K*_a_; pH 7.0 and pD 7.5) [16, 77].

Before investigating fluorescence properties in aqueous media, we first examined the water stability of both flexible MIL‐53‐(OH)_2_ and **rigid L@MIL‐53‐(OH)_2_**. Upon soaking in H_2_O, flexible MIL‐53‐(OH)_2_ exhibited significant particle size reduction, decreasing from 10–30 µm to approximately 500 nm. When filtered, the filtrate contained 20–30 nm nanoparticles. The PXRD pattern of fragmented particles remained consistent with the simulated pattern, indicating retention of crystallinity despite particle fragmentation (Figures S3–S5 and see more detail in the Supporting Information). This reduction in particle size altered the framework flexibility and pore behavior, as shown in CO_2_ adsorption at 195 K (Figure S19). These results suggest that particle size reduction significantly influences framework flexibility and associated properties (e.g., adsorption behavior and fluorescence response), leading to uncontrolled system behavior that limits practical applications [78, 79, 80].

In contrast, **rigid L@MIL‐53‐(OH)_2_** retained its original crystal size after soaking in water (Figure S1). This enhanced stability is attributed to the presence of encapsulated ligands and structural water, which form a cooperative hydrogen‐bonding network that stabilizes the framework under aqueous conditions.

To investigate fluorescence‐based sensing of H_2_O and D_2_O, we first examined the ground‐state asorption of MOFs and the free ligand (H_2_BDC‐(OH)_2_) dispersed in H_2_O or D_2_O. UV–vis spectra were recorded for suspensions of MOFs in H_2_O under stirring conditions. Both flexible MIL‐53‐(OH)_2_ and **rigid L@MIL‐53‐(OH)_2_** exhibited a single absorption band at 350 nm, corresponding to the linker‐based ground‐state transition and matching that of the free ligand (Figure S20). In addition, MOFs dispersed in D_2_O showed identical absorption spectra to those in H_2_O (Figure S20). These results indicate that UV–vis absorption does not distinguish between H_2_O and D_2_O environments in the ground state, including within the linker coordination environment.

Based on the UV–vis results, fluorescence measurements were carried out using excitation at 350 nm to evaluate the feasibility of H_2_O sensing in D_2_O at room temperature. Notably, flexible MIL‐53‐(OH)_2_ after particle size reduction upon soaking in H_2_O, exhibited two emission bands at 450 and 535 nm under stirring conditions; similar behavior was observed in D_2_O, showing two emission bands at 450 and 550 nm (Figures S21 and S22). The combined blue (450 nm) and yellow (550 nm) emissions resulted in a perceived white‐light emission in D_2_O (Figures S21–S23).

This dual emission is attributed to particle size‐dependent behavior: relatively larger particles contribute to the 450 nm emission, while smaller particles including those present in the filtrate, are responsible for the longer‐wavelength emission (535 nm in H_2_O and 550 nm in D_2_O), which differs from the emission of the free ligand, H_2_BDC‐(OH)_2_ that exhibits emission at 515 nm in both H_2_O and D_2_O (Figures S24–S26). To support this hypothesis, the suspension was filtered to remove larger particles while keeping the 20–30 nm particles (Figure S5). The resulting filtrate exhibited a dominant emission peak at 535 nm in H_2_O and at 550 nm in D_2_O, confirming the longer‐wavelength emission originates from smaller particles (Figures S25 and S26).

Next, to confirm the water stability of **rigid L@MIL‐53‐(OH)_2_**, we measured the single‐crystal dimensions before and after soaking in H_2_O (Figure S1). The crystal dimensions remained unchanged even after immersion, confirming the superior structural stability of the framework (Figure S1). This enhanced water stability is attributed to strong hydrogen‐bonding interactions among the encapsulated ligand, structural water molecules, and the framework hydroxyl groups, which collectively reinforce the rigid host‐guest structure and enable reliable fluorescence sensing of H_2_O in D_2_O.

**FIGURE 4:**
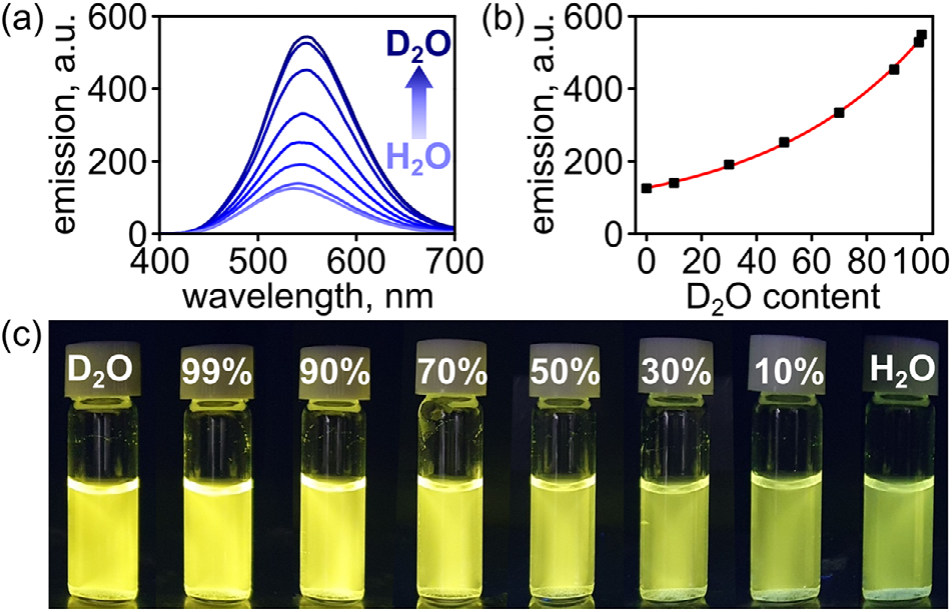
(a) Fluorescence spectra of **rigid L@MIL‐53‐(OH)_2_** at varying H_2_O concentration in D_2_O, excited at 350 nm. The concentration correspond to 100%, 99%, 90%, 70%, 50%, 30%, 10%, and 0% D_2_O at room temperature. (b) Fluorescence spectra intensity of **rigid L@MIL‐53‐(OH)_2_** as a function of D_2_O concentration (*λ*_ex_ = 350 nm). The emission intensity (square) was fitted with a single exponential function (red line), yielding *R* ^2^ = 0.999. (c) Emission images of **rigid L@MIL‐53‐(OH)_2_** in ratio of H_2_O to D_2_O excited at 365 nm.

Based on this structural stability, we investigated the emission properties of **rigid L@MIL‐53‐(OH)_2_** dispersed in H_2_O and D_2_O. The framework exhibited a single emission band (*λ*_ex_ = 350 nm) corresponding to the keto tautomer, appearing at 535 nm in H_2_O and red‐shifted to 550 nm in D_2_O, without any detectable enol tautomer emission (Figure 4). To evaluate its potential for sensing H_2_O in D_2_O, the H_2_O concentration was systematically varied from 0% (pure D_2_O) to 100% (pure H_2_O), resulting in concentration‐dependent changes in emission intensity (Figure 4). The emission intensity was well fitted to a single exponential function and subsequently used to determine the detection limit (Figure 4; Tables S2 and S3) [63, 81]. The detection limit was calculated to be 0.377% of H_2_O in D_2_O, as shown in Tables S2 and S3. These results indicate that the rigidified framework provides sufficient time for ESIPT to relax to the keto state, enabling sensitive H_2_O detection in D_2_O while preserving the structural integrity through hydrogen‐bonded structural water.

In contrast, **rigid L@MIL‐53‐(OH)_2_** after soaking in MeOH, where the framework converts to a flexible phase due to the removal of structural water and encapsulated ligands, exhibited dual emission bands in both H_2_O and D_2_O (450 and 535 nm in H_2_O; 450 and 550 nm in D_2_O) (Figures S27 and S28). The emission at 450 nm is attributed to the enol tautomer, arising from the flexible framework. This behavior is consistent with that observed for flexible MIL‐53‐(OH)_2_ [66, 67].

To further demonstrate the applicability of the *linker‐locking* strategy, we performed post‐synthetic installation of a different type of ligand, 2‐aminoterephthalic acid (H_2_BDC‐NH_2_), into flexible MIL‐53‐(OH)_2_ (see more details in Supporting Information). NH_2_@MIL‐53‐(OH)_2_ was synthesized by a similar method to **rigid L@MIL‐53‐(OH)_2_** using water as a synthesis solvent, yielding a rigid structure containing 0.18 H_2_BDC‐NH_2_ molecules per framework (Figures S29 and S30). Due to its rigid structure, NH_2_@MIL‐53‐(OH)_2_ exhibited a single emission spectrum in both H_2_O and D_2_O (Figures S31 and S32). These results further support the strategy of installing encapsulated ligands and structural water for the rigidification of flexible MOFs, enabling the sensing of H_2_O and D_2_O with a single emission spectrum.

The isotope effect (D_2_O vs H_2_O) increases both the fluorescence intensity and emission wavelength (535 nm in H_2_O and 550 nm in D_2_O). To further quantify these effects, quantum yield (Q.Y.) measurements were performed. Both **rigid L@MIL‐53‐(OH)_2_** and flexible MIL‐53‐(OH)_2_ exhibited higher Q.Y. values in D_2_O (51% and 44%, respectively) compared to H_2_O (12% for both frameworks). The enhanced Q.Y. and red‐shifted emission in D_2_O are attributed to stronger hydrogen‐bonding interactions and isotope effects, which stabilize the excited keto state [82, 83]. As a result, the emission intensity is sufficiently enhanced to allow visual detection, with D_2_O exhibiting the highest brightness and H_2_O the lowest, following the H_2_O concentration in D_2_O (Figure 4).

**FIGURE 5:**
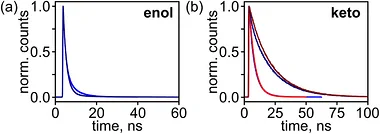
Time‐resolved fluorescence decay profiles of (a) the enol tautomer (*λ*_em_ = 450 nm) for flexible MIL‐53‐(OH)_2_ in H_2_O (blue) and D_2_O (dark blue), and (b) the keto tautomer for flexible MIL‐53‐(OH)_2_ (blue/dark blue) and **rigid L@MIL‐53‐(OH)_2_** (red/dark red) in H_2_O (*λ*_em_ = 535 nm) and D_2_O (*λ*_em_ = 550 nm). All profiles were obtained upon photoexcitation at 377 nm (*λ*_ex_).

Time‐resolved fluorescence lifetime measurements were performed to gain mechanistic insight into the ESIPT process in both flexible MIL‐53‐(OH)_2_ and **rigid L@MIL‐53‐(OH)_2_** dispersed in H_2_O and D_2_O (Figure 5 and Table S1). Based on steady‐state emission spectra, three representative emission wavelengths were selected: 450 nm (enol in both H_2_O and D_2_O systems), 535 nm (keto emission in H_2_O), and 550 nm (keto emission in D_2_O).

Flexible MIL‐53‐(OH)_2_ exhibited dual emission corresponding to enol and keto tautomers, each with distinct excited‐state lifetimes (Figure 5). In H_2_O, lifetimes (*τ*_avg_) of 1.95 ns (*λ*_em_ = 450 nm, enol) and 4.23 ns (*λ*_em_ = 535 nm, keto) were observed. In D_2_O, the enol lifetime remained similar (*τ*_avg_ = 1.76 ns at 450 nm), whereas the keto lifetime increased significantly to 14.24 ns (*λ*_em_ = 550 nm) (Figure 5 and Table S1). The marginal variation in enol lifetime suggests that the enol decay pathway is largely decoupled from the isotopic nature of the solvent, whereas the keto state exhibits a pronounced primary kinetic isotope effect. In contrast, a more than threefold increase in keto lifetime in D_2_O reflects substantial stabilization of the keto excited state via stronger hydrogen bonding between framework and D_2_O and reduced vibrational relaxation, consistent with a kinetic isotope effect [84, 85].

In contrast to the flexible framework, **rigid L@MIL‐53‐(OH)_2_** exhibits exclusively keto‐form emission with no detectable enol contribution (Figure 4). The measured lifetimes are *τ*_avg_ = 4.54 ns at 535 nm in H_2_O and *τ*_avg_ = 17.75 ns at *λ*_em_ = 550 nm in D_2_O, closely matching the keto lifetimes observed in the flexible system (Figure 5 and Table S1). The absence of an enol component suggests that framework rigidification constrains the linker in ESIPT‐favorable geometries (*linker‐locking*), thereby minimizing the enol‐like linker population in the rigid framework and enabling complete conversion to the keto state before radiative relaxation.

This pronounced isotope dependence highlights that the keto tautomer governs H_2_O/D_2_O discrimination, whereas the enol tautomer does not significantly contribute to sensing. In H_2_O, the Q.Y. of the keto‐tautomer emission is markedly suppressed from 51% to 12%, and its emission lifetime is shortened from 17.75 to 4.54 ns compared with those in D_2_O. This indicates that the keto tautomer undergoes more efficient nonradiative quenching in H_2_O, rather than an acceleration in its radiative decay channel. This quenching process can be attributed to solvent‐assisted vibrational coupling with nearby O─H oscillators located in the local solvation or hydrogen‐bonding environment of the keto tautomer. Maillard et al. reported that O─H containing solvents can act as efficient fluorescence quenchers through resonant energy transfer from electronically excited fluorophores to O─H vibrational overtone and combination modes, particularly for fluorophores that emit at lower energies [85]. In contrast, O─D vibrational modes in D_2_O have lower frequencies than O─H modes, shifting the corresponding overtone/combination bands to lower energies. Consequently, coupling of the keto excited state to O─D modes is less efficient because it requires higher‐order overtone or combination transitions with weaker oscillator strengths, making D_2_O a much weaker vibrational quencher.

**Rigid L@MIL‐53‐(OH)_2_** displays substantially stronger hydrogen bonding interactions that stabilize and rigidify the framework. The crystal structure reveals a strong hydrogen bond between the framework *µ*‐hydroxo group and an encapsulated ligand (H···O distance = 1.88 Å; O─H···O angle = 173.1°). Furthermore, three structural water molecules are involved in extensive hydrogen‐bonding interactions with the framework ligand or the encapsulated ligand (Figure S37 and Table S6). These strengthened and directional hydrogen‐bonding interactions rigidify the local environment and enhance proton transfer efficiency, thereby promoting ESIPT and yielding pronounced keto emission in **rigid L@MIL‐53‐(OH)_2_**. Overall, the crystallographic and fluorescence data reveal a direct correlation between hydrogen‐bonding interactions and ESIPT efficiency. The observed enhancement of hydrogen bonding upon encapsulated ligand and structural water incorporation in **rigid L@MIL‐53‐(OH)_2_** and the corresponding increase in ESIPT response provide strong experimental support for the proposed hydrogen bond strength dependent ESIPT mechanism [54, 86]. However, we believe that theoretical energetic calculations for **rigid L@MIL‐53‐(OH)_2_** with encapsulated ligand and water molecule and flexible MIL‐53‐(OH)_2_ relating hydrogen bonding strength with ESIPT efficiency will be helpful for further mechanistic support and therefore remains interesting topic for future interest. Consequently, ligand encapsulation provides an effective strategy for tuning and controlling ESIPT‐based fluorescence through framework rigidity and host‐guest interactions.

In contrast, in flexible MIL‐53‐(OH)_2_, weak intramolecular hydrogen bonding is observed between the ligand hydroxyl groups and the metal‐bound carboxylate oxygen atoms (H···O distance = 1.95 Å; O─H···O angle = 125.8°) [63]. Such insufficient donor–acceptor coupling suppresses efficient ESIPT, resulting predominantly in enol emission in flexible phase. Because of the extremely small size of fragmented nanoparticles formed in aqueous media, single‐crystal structural determination was not feasible; thus, the structural origin of their keto emission could not be directly established.

**FIGURE 6:**
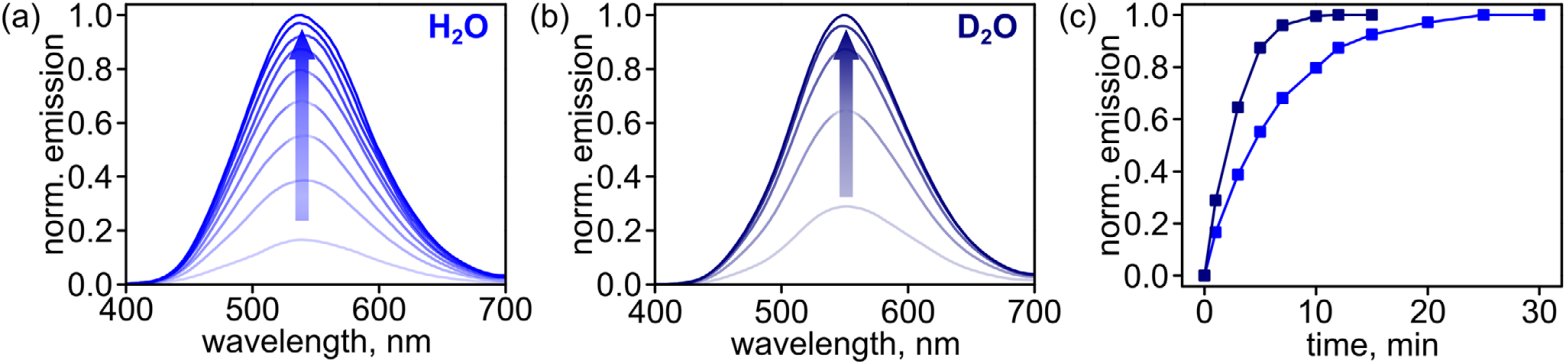
Time‐dependent fluorescence spectra of **rigid L@MIL‐53‐(OH)_2_** upon soaking in (a) H_2_O and (b) D_2_O (*λ*_ex_ = 350 nm). (c) Emission kinetic profiles in H_2_O (blue) and D_2_O (dark blue) over time.

A rapid response time is critical for the prompt detection of target analytes for real‐world sensing applications [2, 6, 7, 8]. To evaluate the kinetic performance of **rigid L@MIL‐53‐(OH)_2_**, we conducted time‐dependent fluorescence measurements upon immersion in H_2_O and D_2_O (Figure 6). Remarkably, this is the first study to compare dynamic sensing response times mediated by solvent isotope effect using MOFs. **Rigid L@MIL‐53‐(OH)_2_** reached its fully emissive state within 10 min in D_2_O, more than twice as fast as in H_2_O (25 min) (Figure 6). This accelerated response in D_2_O can be attributed to the stronger hydrogen‐bonding interactions and distinct zero‐point vibrational energy associated with deuterium, which facilitate faster guest‐framework binding dynamics and local structural stabilization relative to protium [87].

We further found that hydrogen‐bonding interactions between water molecules and the host frameworks are strongly influenced by both proton concentration (pH) and thermal vibrational dynamics of proton (temperature), which provides the potential for dual parameter sensing of pH and temperature. We first confirmed the chemical stability of **rigid L@MIL‐53‐(OH)_2_** across a pH range of 1–10 using PXRD (Figure S33). We subsequently evaluated its emission responsiveness as a function of pH, which revealed effective sensing capability below pH 4 with clear discrimination between pH 1 and 3 (Figure S34). This behavior aligns with the primary acid dissociation (p*K*_a1_ = 4.16) of H_2_BDC‐(OH)_2_ corresponding to protonation/deprotonation behaviors within this pH regime [88].

Furthermore, because excited‐state luminescence is strongly influenced by hydrogen‐bonding interactions and molecular vibrational dynamics, we conducted temperature‐dependent fluorescence measurements (30°C–90°C) in H_2_O and D_2_O to assess their differentiation at elevated temperatures (Figure S35) [45]. In both media, **rigid L@MIL‐53‐(OH)_2_** exhibited a monotonic decrease in emission intensity with increasing temperature due to enhanced vibrational non‐radiative decay and thermal perturbation of the hydrogen‐bonding network. Notably, even at 90°C, clear discrimination between H_2_O and D_2_O was maintained ($I_{D_{2}O}$/$I_{H_{2}O}$ = 2.2). The slight drop from the room‐temperature ratio (3.1) can be attributed to temperature‐dependent variations in the relative acidities and hydrogen‐bonding strengths of H_2_O and D_2_O [89].

**FIGURE 7:**
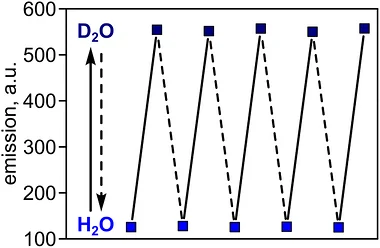
Cyclability of **rigid L@MIL‐53‐(OH)_2_** in H_2_O and D_2_O over five cycles. Solid lines correspond to the activated sample after soaking in D_2_O, while dashed lines correspond to the activated sample after soaking in H_2_O.

For practical sensing applications, heterogeneous sensors are desirable due to their inherent reusability; however, such systems require high structural stability under operating conditions [4, 6, 7]. Given that **rigid L@MIL‐53‐(OH)_2_** remains stable in aqueous media while exhibiting a single keto emission in both H_2_O and D_2_O with distinct intensities, we evaluated its recyclability (Figure 7). In a typical cycle, **rigid L@MIL‐53‐(OH)_2_** was first soaked in H_2_O and its fluorescence emission was measured, followed by activation at 150°C for one day. The sample was then immersed in D_2_O, and the fluorescence emission was recorded again. This H_2_O/D_2_O cycling process was repeated for five cycles. The emission profiles remained consistent in both solvents throughout all cycles, demonstrating superior recyclability and structural robustness (Figure 7). Furthermore, PXRD analysis confirmed that the crystallinity of **rigid L@MIL‐53‐(OH)_2_** was fully preserved after five cycles without degradation (Figure S36). Additionally, the use of water as a green synthesis solvent lowers the overall production cost to approximately $0.07/g at the industrial scale compared to $34.21/g at the laboratory scale, highlighting its potential economic viability for reusable, real‐world sensor applications (Table S4 and see Supporting Information for detailed cost calculations). These results indicate that the rigid framework is well suited for reusable heterogeneous sensing systems and may also be applicable to separation systems.

## 3 Conclusion

In summary, we demonstrate a *linker‐locking* strategy to rigidify flexible MIL‐53‐(OH)_2_ through incorporation of encapsulated ligands and hydrogen‐bonded structural water, generating a stable rigid phase, **rigid L@MIL‐53‐(OH)_2_**. This rigidification suppresses framework flexibility and enables precise control over ESIPT, converting mixed enol/keto emission into a single keto‐form emission that allows discrimination between H_2_O and D_2_O.

The cooperative hydrogen‐bonding network among the framework, encapsulated ligands, and structural water significantly enhances water stability, preventing particle fragmentation and preserving crystallinity in aqueous environments. In contrast, flexible MIL‐53‐(OH)_2_ undergoes particle size reduction and exhibits heterogeneous emission behavior, precluding reliable sensing. The rigid phase can be reversibly interconverted with the flexible phase through removal and reintroduction of encapsulated ligands and structural water, highlighting the dynamic nature of the linker‐locking process.

Time‐resolved fluorescence measurements reveal that sensing originates from the keto tautomer, which exhibits pronounced isotope‐dependent lifetimes (>14 ns in D_2_O vs <5 ns in H_2_O), whereas the enol tautomer remains insensitive to isotopic substitution. This result provides the first demonstration that framework rigidification stabilizes the excited keto state via hydrogen‐bonding interactions, enabling sensitive detection of trace H_2_O in D_2_O with a two‐fold faster detection time.

Overall, this work establishes linker locking as a new strategy for stabilizing flexible MOFs and controlling excited‐state processes. By coupling structural rigidity with tunable host‐guest interactions, this approach provides a versatile platform for designing functional materials for sensing, photophysics, and catalysis in aqueous environments.

## Author Contributions

**Amitosh Sharma**: writing – original draft, writing – review and editing, software, conceptualization, data curation, formal analysis, methodology. **Seonghwan Lee**: writing – review and editing, writing – original draft, data curation, formal analysis. **Hak‐Won Nho**: formal analysis, writing – review and editing, data curation. **Junmo Seong**: data curation, formal analysis, writing – review and editing. **Eunseo Lee**: writing – review and editing, data curation, formal analysis. **Oh‐Hoon Kwon**: funding acquisition, writing – review and editing, resources. **Myoung Soo Lah**: supervision, writing – review and editing, resources. **Jaewoong Lim**: conceptualization, methodology, software, data curation, investigation, validation, formal analysis, supervision, funding acquisition, visualization, project administration, resources, writing – original draft, writing – review and editing.

## Conflicts of Interest

The authors declare no conflicts of interest.

## Supporting information




**Supporting File**: advs77856‐sup‐0001‐SuppMat.pdf.

## Data Availability

The data that supports the findings of this study are available in the supplementary material of this article. Deposition numbers 2545529 (for rigid L@MIL‐53‐(OH)2) Contains the Supplementary Crystallographic Data for this paper. These data are Provided Free of Charge by the Joint Cambridge Crystallographic Data Centre and Fachinformationszentrum Karlsruhe Access Structures service.
