# Supplementary material for: Linker‐Locking Strategy for the Rigidification of Flexible Metal–Organic Framework: Structural Stabilization and Controlled ESIPT for H2O/D2O Sensing
Source: Adv Sci (Weinh). 2026 Sep 27:e77856. Online ahead of print. doi: 10.1002/advs.77856 (PMC13616277; doi:10.1002/advs.77856)
Supplement: Supplementary file 1 [file ADVS-9999-e77856-s001.pdf]

Supporting Information
©Wiley-VCH 2021
69451 Weinheim, Germany

Linker-Locking Strategy for the Rigidification of Flexible Metal-Organic Framework: Structural Stabilization and Controlled ESIPT for H_2O/D_2O Sensing

A. Sharma,[+] S. Lee,[+] S. I. Park,[+] H.-W. Nho, J. Seong, E. Lee, O.-H. Kwon,[*] M. S. Lah,[*] and J. Lim[*]

Abstract: Controlling the photodynamics of excited-state intramolecular proton transfer (ESIPT) fluorophores is crucial for sensing, due to their large Stokes shifts and dual emission. However, structural flexibility and solvent-induced degradation often hinder isolating a single tautomeric emission in water. Here, we introduce a *linker-locking strategy* that rigidifies the flexible metal-organic framework (MOF) MIL-53-$(OH)_2$ through cooperative stabilization by encapsulated ligands and structural water, forming a stable phase, **rigid L@MIL-53-$(OH)_2$**. The hydrogen-bonding network among structural water, encapsulated ligands, and the framework stabilizes the pore structure while preserving reversible interconversion between flexible and rigid phases via ligand and water removal or post-encapsulation. This rigidification suppresses particle fragmentation in aqueous media and enables controlled ESIPT behavior. While flexible MIL-53-$(OH)_2$ exhibits mixed enol and keto emissions due to size-dependent heterogeneity, **rigid L@MIL-53-$(OH)_2$** displays a single keto emission with prolonged excited-state lifetimes (> 14 ns in D_2O vs < 5 ns in H_2O) and distinct optical responses in H_2O and D_2O. Consequently, the pronounced kinetic isotope effect allows **rigid L@MIL-53-$(OH)_2$** to sensitively detect trace H_2O in D_2O with high reusability over at least five cycles. This work establishes *linker locking* as a general strategy for stabilizing flexible MOFs and controlling excited-state processes for aqueous sensing.

DOI: 10.1002/anie.2021XXXXX

Table of Contents

1. Materials S4
2. Preparation of **rigid L@MIL-53-(OH)$_2$** S4
3. Preparation of flexible MIL-53-(OH)$_2$ S4
4. Leaching test of **rigid L@MIL-53-(OH)$_2$** after soaking in solvents S5
5. Preparation of L@MIL-53-(OH)$_2$ through post-encapsulation S5
6. Preparation of NH_2@MIL-53-(OH)$_2$ through post-encapsulation S6
7. Preparation of filtrate of flexible MIL-53-(OH)$_2$ after soaking in H_2O S6
8. General procedures S6
9. UV-vis spectroscopy S6
10. Fluorescence spectroscopy S7
11. Time-resolved fluorescence decay measurements S7
12. **Table S1**. Fluorescence lifetimes of flexible MIL-53-(OH)$_2$ and **rigid L@MIL-53-(OH)$_2$** dispersed in H_2O and D_2O S7
13. Calculation of detection limit S7
14. **Table S2**. Fluorescence intensities of **rigid L@MIL-53-(OH)$_2$** dispersed in D_2O (99.9%) S8
15. **Table S3**. Parameters used for the detection limit calculation S8
16. Calculation **of rigid L@MIL-53-(OH)$_2$** production cost at laboratory and industrial scale S8
17. **Table S4**. Parameter breakdown and cost analysis for the production of **rigid L@MIL-53-(OH)$_2$** at laboratory and industrial scales S9
18. X-ray crystal structure determination of **rigid L@MIL-53-(OH)$_2$** S10
19. **Table S4**. X-ray crystal structure refinement data for **rigid L@MIL-53-(OH)$_2$** S11
20. **Table S5**. Hydrogen bonds and close contacts information between the framework and encapsulated H_2BDC-(OH)$_2$/water molecules S12
21. **Table S6**. π-π stacking interactions information between the framework and encapsulated H_2BDC-(OH)$_2$ S12
22. **Figure S1**. Microscope images of **rigid L@MIL-53-(OH)$_2$** S13
23. **Figure S2**. TGA of **rigid L@MIL-53-(OH)$_2$** S13
24. **Figure S3**. PXRD patterns of flexible MIL-53-(OH)$_2$ S14
25. **Figure S4**. SEM images of flexible MIL-53-(OH)$_2$ before and after soaking in H_2O S15
26. **Figure S5**. TEM images of the dried filtrate after soaking flexible MIL-53-(OH)$_2$ in H_2O S15
27. **Figure S6**. N_2 adsorption isotherm of flexible MIL-53-(OH)$_2$, **rigid L@MIL-53-(OH)$_2$**, and **rigid L@MIL-53-(OH)$_2$-150a** at 77 K. S16
28. **Figure S7**. H_2O vapor adsorption isotherm of flexible MIL-53-(OH)$_2$, **rigid L@MIL-53-(OH)$_2$**, and **rigid L@MIL-53-(OH)$_2$-150a** at 298 K. S16
29. **Figure S8**. ^{1}H NMR spectrum of **rigid L@MIL-53-(OH)$_2$** in CD_3OD S17
30. **Figure S9**. TGA of **rigid L@MIL-53-(OH)$_2$** after soaking in MeOH S17
31. **Figure S10**. PXRD patterns of **rigid L@MIL-53-(OH)$_2$** after soaking in MeOH S18
32. **Figure S11**. CO_2 adsorption isotherm of **rigid L@MIL-53-(OH)$_2$** after soaking in MeOH at 195 K S18
33. **Figure S12**. PXRD patterns of **rigid L@MIL-53-(OH)$_2$** after soaking in acetone S19
34. **Figure S13**. PXRD patterns of **rigid L@MIL-53-(OH)$_2$** after soaking in DMF S19
35. **Figure S14**. PXRD patterns of **rigid L@MIL-53-(OH)$_2$** after soaking in hexane S20
36. **Figure S15**. PXRD patterns of **rigid L@MIL-53-(OH)$_2$** after soaking in toluene S20
37. **Figure S16**. TGA of L@MIL-53-(OH)$_2$ prepared by post-encapsulation in H_2O S21
38. **Figure S17**. PXRD patterns of L@MIL-53-(OH)$_2$ prepared by post-encapsulation in MeOH S22

39. Figure S18. TGA of L@MIL-53-(OH)$_2$ prepared by post-encapsulation in MeOH **S22**
40. Figure S19. CO_2 adsorption isotherm of flexible MIL-53-(OH)$_2$ after soaking in H_2O at 195 K **S23**
41. Figure S20. Normalized UV-vis spectra of H_2BDC-(OH)$_2$, flexible MIL-53-(OH)$_2$, and **rigid L@MIL-53-(OH)$_2$** in H_2O and D_2O **S23**
42. Figure S21. Fluorescence spectra of flexible MIL-53-(OH)$_2$ at varying H_2O/D_2O ratios **S24**
43. Figure S22. Fluorescence intensity of flexible MIL-53-(OH)$_2$ with H_2O/D_2O ratio at 450 nm and 550 nm **S24**
44. Figure S23. Emission images of flexible MIL-53-(OH)$_2$ dispersed in H_2O and D_2O **S25**
45. Figure S24. Fluorescence spectra of H_2BDC-(OH)$_2$ in H_2O and D_2O **S25**
46. Figure S25. Fluorescence spectra of bulk flexible MIL-53-(OH)$_2$ crystals and the filtrate obtained after crystal removal following soaking in H_2O **S26**
47. Figure S26. Fluorescence spectra of bulk flexible MIL-53-(OH)$_2$ crystals and the filtrate obtained after crystal removal following soaking in D_2O **S26**
48. Figure S27. Fluorescence spectra of **rigid L@MIL-53-(OH)$_2$** after soaking in MeOH measured in H_2O **S27**
49. Figure S28. Fluorescence spectra of **rigid L@MIL-53-(OH)$_2$** after soaking in MeOH measured in D_2O **S27**
50. Figure S29. PXRD patterns of NH_2@MIL-53-(OH)$_2$ prepared by post-encapsulation **S28**
51. Figure S30. ^{1}H NMR spectrum of NH_2@MIL-53-(OH)$_2$ **S28**
52. Figure S31. Fluorescence spectra of H_2BDC-NH_2, NH_2@MIL-53-(OH)$_2$, and flexible MIL-53-(OH)$_2$ measured in H_2O **S29**
53. Figure S32 Fluorescence spectra of H_2BDC-NH_2, NH_2@MIL-53-(OH)$_2$, and flexible MIL-53-(OH)$_2$ measured in D_2O **S29**
54. Figure S33. PXRD pattern of **rigid L@MIL-53-(OH)$_2$** for chemical stability **S30**
55. Figure S34. Fluorescence spectra of **rigid L@MIL-53-(OH)$_2$** measured at pH 1−10 **S30**
56. Figure S35. Fluorescence spectra of **rigid L@MIL-53-(OH)$_2$** measured at various temperatures in H_2O and D_2O **S31**
57. Figure S36. PXRD patterns of **rigid L@MIL-53-(OH)$_2$** after recyclability test **S31**
58. Figure S37. Crystal structure of **rigid L@MIL-53-(OH)$_2$** **S32**
59. References **S33**

Materials.

Indium nitrate hydrate (99.999%, Sigma Aldrich), 2,5-dihydroxyterephthalic acid (98%, Sigma-Aldrich), 2-aminoterephthalic acid (98%, TCI), sodium hydroxide (97%, Junsei), *N*,*N*-dimethylformamide (99.5%, Junsei), acetone (99.5%, Junsei), methanol (99.8%, Junsei), hexane (99.5%, Junsei), toluene (99.8%, Sigma Aldrich), hydrochloric acid (37%, Sigma Aldrich), methanol-d_4 (99.8%, Cambridge Isotope Laboratories, Inc), dmso-d_6 (99.5%, Cambridge Isotope Laboratories, Inc), sulfuric acid-d_2 (99.5%, Sigma Aldrich), and deuterium oxide (99.98%, Cambridge Isotope Laboratories, Inc.) were used as received.

Preparation of rigid L@MIL-53-(OH)$_2$.

To a 20-mL vial, $In(NO_3)_3 \cdot xH_2O$ (225 mg, 0.750 mmol) was dissolved in 10.0 mL of H_2O and 2,5-dihydroxy-1,4-benzenedicarboxylic acid (H_2BDC-$(OH)_2$) (99.0 mg, 0.500 mmol) was added to the solution. The resulting solution was then heated in an oven at 100 °C for 24 hours. After 24 hours, the yellow crystals with dimensions of 10–30 µm were harvested and were washed with hot H_2O (5 × 10.0 mL) (Figure S1). The crystals of $[In(OH)((BDC)\text{-}(OH)_2)] \cdot (H_2BDC\text{-}(OH)_2)_{0.6} \cdot (H_2O)_2$ (0.114 g) were isolated in 47% yield and suitable for single-crystal X-ray diffraction studies (Figures 1 and S37). Refinement details and a description of the data collection are given below in Tables S5–S7. The powder X-ray diffraction (PXRD) patterns of the as-synthesized sample of rigid L@MIL-53-(OH)$_2$ matched the simulated pattern from single crystal data (Figures 2 and 3). The formula was determined using thermogravimetric analysis (TGA)[1] and elemental analysis (EA) indicating a composition of 1.6 H_2BDC-$(OH)_2$ per formula unit: 1.0 BDC-$(OH)_2^-$ (framework linker) and 0.60 H_2BDC-$(OH)_2$ (encapsulated ligand), and 2.0 H_2O (Figure S2). EA of $[In(OH)(BDC\text{-}(OH)_2)] \cdot (H_2BDC\text{-}(OH)_2)_{0.60} \cdot (H_2O)_{2.0}$, $InC_{12.8}H_{12.6}O_{12.6}$, fw = 482.85 g/mol. Calculated: In, 23.8; C, 31.8; H, 2.63%. Found: In, 24.0; C, 31.5; H, 2.57%. N_2 (77 K) and CO_2 (195 K) adsorption measurements showed that the framework activated at room temperature for 24 hours removed free solvent molecules contained in the pores accessible to CO_2 but not to N_2 molecules (Figures 2 and S6). After removal of structural water by activation at 150 °C for 24 hours, the framework (denoted L@MIL-53-(OH)$_2$-150a) collapsed and exhibited gate-opening behavior in CO_2 adsorption isotherm at 195 K (Figures 2 and S6). H_2O (298 K) adsorption measurements demonstrated increased hydrophilicity after removal of structural water (activation at 150 °C for 24 hours) (Figure S7). The L@MIL-53-(OH)$_2$-150a was converted to rigid L@MIL-53-(OH)$_2$ after exposure to 98% relative humidity (RH) at 25 °C for one hour in a humidity chamber or soaking in H_2O, as confirmed by PXRD patterns in Figure 2.

Preparation of flexible MIL-53-(OH)$_2$.

The preparation of flexible MIL-53-(OH)$_2$ was based on a modified literature procedure.[2] To a 20-mL vial, $In(NO_3)_3 \cdot xH_2O$ (150 mg, 0.500 mmol) was dissolved in 5.00 mL of H_2O. Separately, H_2BDC-$(OH)_2$ (99.0 mg, 0.500 mmol) was dissolved in 5.00 mL of *N*,*N*-dimethylformamide (DMF) in a second 20-mL vial. The two solutions were then mixed and the resulting solution was then heated in an oven at 70 °C for 24 hours. After cooling to room temperature, the colorless crystals with dimensions of 20–50 µm were harvested and were washed with DMF (4 × 10.0 mL) and acetone (4 × 10.0 mL) (Figure S4). The crystals of $In(OH)((BDC)\text{-}(OH)_2)$ (0.135 g) were isolated in 62% yield. The PXRD pattern of the as-synthesized sample of $In(OH)(BDC\text{-}(OH)_2)$ matched the simulated pattern from single crystal data[2] (Figure S3). Flexible MIL-53-(OH)$_2$ was activated at 120°C for 24 hours to remove solvent molecules

from the pores. Subsequent N_2 (77 K), CO_2 (195 K), and H_2O (298 K) adsorption measurements demonstrated the flexible MOF behavior of the framework (Figures 2, S6, and S7).

Leaching test of rigid L@MIL-53-(OH)$_2$ after soaking in solvents.

To evaluate the stability of the encapsulated ligand and structural water in rigid L@MIL-53-(OH)$_2$ after solvent exposure, the crystals were soaked in various solvents, including a protic solvent (MeOH), aprotic solvents (DMF and acetone), and nonpolar solvents (hexane and toluene), at room temperature or elevated temperatures (100 or 120 °C) for periods ranging from one to 14 days. In MeOH, a protic solvent, the encapsulated ligand was detected after soaking in CD_3OD by ^{1}H nuclear magnetic resonance (NMR) result (Figure S8). Release of the encapsulated ligand and structural water from the framework was further confirmed by TGA, which gave a formula of $[In(OH)(BDC\text{-}(OH)_2)]\cdot(H_2BDC\text{-}(OH)_2)_{0.18}\cdot(H_2O)_{1.0}$ (Figure S9). After the loss of the encapsulated ligand and structural water, rigid L@MIL-53-(OH)$_2$ converted to the identical structure as flexible MIL-53-(OH)$_2$ in MeOH, as confirmed by PXRD (Figures 3 and S10). Consistent with the structural transformation, the framework exhibited flexible behavior, showing gate-opening behavior in the CO_2 adsorption isotherm at 195 K (Figures 3 and S11).[3]

Similarly, in the aprotic solvents DMF and acetone, in which H_2BDC-(OH)$_2$ is soluble, rigid L@MIL-53-(OH)$_2$ maintained its structure after soaking at room temperature for one day (Figures S12 and S13). However, after seven days in acetone, the structure transformed to flexible MIL-53-(OH)$_2$ (Figure S14). In DMF, the framework remained stable at room temperature for 14 days but converted to flexible MIL-53-(OH)$_2$ after heating at 120 °C for one day (Figure S13). In the nonpolar solvents hexane and toluene, in which H_2BDC-(OH)$_2$ is insoluble, rigid L@MIL-53-(OH)$_2$ maintained its structure after soaking at room temperature for 14 days (Figures S14 and S15). In toluene at 100 °C, the framework also maintained its structure but exhibited slightly decreased the crystallinity (Figure S15).

Preparation of L@MIL-53-(OH)$_2$ through post-encapsulation.

For the preparation of L@MIL-53-(OH)$_2$ through the post-encapsulation of H_2BDC-(OH)$_2$ into flexible MIL-53-(OH)$_2$, freshly synthesized flexible MIL-53-(OH)$_2$ was harvested, thoroughly washed with DMF and acetone, and then dried in air overnight. After drying, flexible MIL-53-(OH)$_2$ (66.0 mg, corresponding to 0.200 mmol of BDC-(OH)$_2$) was placed in a 20-mL vial, and H_2BDC-(OH)$_2$ (79.0 mg, 0.400 mmol) and 10.0 mL of H_2O were added. The vial was placed in a preheated oven at 100 °C for 24 hours. After 24 hours, the yellow crystals were collected and washed with hot H_2O (5 × 10.0 mL). The crystallinity, structural transformation, and rigidification of $[In(OH)((BDC)\text{-}(OH)_2)]\cdot(H_2BDC\text{-}(OH)_2)_{0.27}\cdot(H_2O)_{1.0}$ were confirmed by PXRD, and the composition was determined by TGA result (Figures 3 and S16).

For the preparation of L@MIL-53-(OH)$_2$ in a synthetic solvent (MeOH) instead of H_2O, flexible MIL-53-(OH)$_2$ (66.0 mg, corresponding to 0.200 mmol of BDC-(OH)$_2$) was placed in a 20-mL vial, and H_2BDC-(OH)$_2$ (79.0 mg, 0.400 mmol) and 10.0 mL of MeOH were added. The vial was kept at room temperature for 24 hours. After 24 hours, the yellow crystals were collected by filtration and thoroughly washed with MeOH (2.00 mL). The crystals maintained a structure similar to flexible MIL-53-(OH)$_2$ in MeOH and exhibited flexible behavior in PXRD, although the framework contained encapsulated ligands, which gave a composition of $[In(OH)((BDC)\text{-}(OH)_2)]\cdot(H_2BDC\text{-}(OH)_2)_{0.25}\cdot(H_2O)_{1.0}$ by TGA result (Figures S18 and S19).

Preparation of NH_2@MIL-53-$(OH)_2$ through post-encapsulation.

For preparation of NH_2@MIL-53-$(OH)_2$ through the post-synthetic installation of 2-aminoterephthalic acid (H_2BDC-NH_2) into flexible MIL-53-$(OH)_2$, freshly synthesized flexible MIL-53-$(OH)_2$ was harvested, thoroughly washed with DMF and acetone, and then dried on air for overnight. After drying, flexible MIL-53-$(OH)_2$ (66.0 mg, corresponding to 0.200 mmol of BDC-$(OH)_2$) was placed in a 20-mL vial, and H_2BDC-NH_2 (72.0 mg, 0.400 mmol) and 10.0 mL of H_2O were added. The vial was placed in a preheated oven at 100 °C for 24 hours. After 24 hours, the yellow crystals were harvested and washed using hot H_2O (5 × 10.0 mL). The structural transformation into the rigid phase and the crystallinity of $[In(OH)((BDC)\text{-}(OH)_2)]\cdot(H_2BDC\text{-}NH_2)_{0.18}$ were confirmed by PXRD analysis (Figure S29). The number of encapsulated ligands of NH_2@MIL-53-$(OH)_2$ was estimated using ^{1}H NMR data (Figure S30).

Preparation of flexible MIL-53-$(OH)_2$ after soaking in H_2O.

Flexible MIL-53-$(OH)_2$ was soaked in H_2O for one hour and then separated into a filtrate and solid precipitate by filtration. The filtrate was dried under vacuum, and the resulting particles exhibited sizes of 20–30 nm in TEM images (Figure S5). The solid precipitate of flexible MIL-53-$(OH)_2$ showed reduced particle size after soaking in H_2O and a change in the gate-opening pressure of the CO_2 adsorption isotherm at 195 K due to particle size reduction (Figures S4 and S19).[4]

General procedures.

All reagents were purchased from commercial sources and used without further purification. PXRD data were recorded using a Bruker D2 Phaser automated diffractometer at room temperature, with a step size of 2θ = 0.02°. Elemental analyses (EA) were conducted using a Flash 2000 elemental analyzer. ^{1}H nuclear magnetic resonance (NMR) spectra were obtained using an Agilent 400-MR DD2 NMR spectrometer operating at 400 MHz for ^{1}H. TGA was performed using a Q-600 series instrument from TA Instruments at a heating rate of 5 ºC min^{-1} under flowing air. Scanning electron microscopy (SEM) was performed using SU-8220 from Hitachi. High-resolution transmission electron microscopy (HR-TEM) was performed using a JEOL USA JEM-2100F (Cs corrector). Gas sorption isotherms were measured using a BELSORP-max (BEL Japan, Inc.) adsorption system, employing a standard volumetric technique up to saturation pressure. N_2 (99.999%) and CO_2 (99.999%) sorption isotherms were monitored at 77 K and 195 K, respectively. All UV-Vis spectra were collected using a Cary 5000 (Agilent) UV-Vis-NIR (200–3300 nm) spectrophotometer. Fluorescence spectra were collected with a Cary Eclipse fluorometer (Varian) with a 5 nm slit width. Quantum yields of MOFs were collected using a FP-8500ST spectrofluorometer (Jasco International).

UV-vis spectroscopy.

UV–vis spectra were collected by dispersing 0.50 mg of MOF crystals or ligand in a cuvette containing 2.5 mL of H_2O (or D_2O). UV–vis spectra were recorded in the range of 200–800 nm.

Fluorescence spectroscopy.

Fluorescence spectra were collected by dispersing 1.20 mg of MOF crystals in a cuvette containing 2.5 mL of H_2O or D_2O (or a series of H_2O–D_2O mixtures). The suspension was excited at 350 nm, and the fluorescence emission was recorded in the range of 370–700 nm. The suspension was continuously stirred at a constant rate to maintain homogeneity.

Time-resolved fluorescence decay measurements.

Time-resolved fluorescence decay profiles were obtained using a time-correlated single-photon counting spectrofluorometer (Fluotime 300, PicoQuant) equipped with a 375-nm picosecond laser diode (LDH-D-C-375, PicoQuant) as the excitation source. The instrumental response function was 150 ps. The fluorescence decay profiles were fitted using software provided by PicoQuant Instruments. The emission decay kinetics of the MOFs were fitted using a multiexponential decay function.[5,6] The average emission lifetime (τ_{avg}) was obtained from the best fitting (Table S1). The emission wavelengths were selected based on the peaks in the fluorescence emission spectra: flexible MIL-53-$(OH)_2$ in H_2O (450 nm and 535 nm), flexible MIL-53-$(OH)_2$ in D_2O (450 nm and 550 nm), rigid L@MIL-53-$(OH)_2$ in H_2O (535 nm), and rigid L@MIL-53-$(OH)_2$ in D_2O (550 nm) (Figure 5).

Table S1. Fluorescence lifetimes of flexible MIL-53-$(OH)_2$ and rigid L@MIL-53-$(OH)_2$ dispersed in H_2O and D_2O.

sample	emission, nm	τ_{avg}, ns	χ^2
flexible MIL-53-$(OH)_2$ in H_2O	450	1.95	1.183
flexible MIL-53-$(OH)_2$ in D_2O	450	1.76	1.108
flexible MIL-53-$(OH)_2$ in H_2O	535	4.23	1.140
flexible MIL-53-$(OH)_2$ in D_2O	550	14.24	1.359
**rigid L@MIL-53-$(OH)_2$ in H_2O**	535	4.54	1.210
**rigid L@MIL-53-$(OH)_2$ in D_2O**	550	17.75	1.696

Calculation of detection limit.

The detection limit was calculated based on $3\sigma/m$, where σ is the standard deviation of the fluorescence intensity of the blank solution and m is the slope of the calibration curve.[2,7] The standard deviation (σ) was calculated from the fluorescence intensities of five independent measurements of rigid L@MIL-53-$(OH)_2$ (0.40 mg) dispersed in 2.5 mL of D_2O (99.9%) under continuous stirring (Table S2). These measurements were used to determine the average intensity (I_{avg}) and the standard deviation (σ).

Table S2. Fluorescence intensities of rigid L@MIL-53-$(OH)_2$ dispersed in D_2O (99.9%).

entry number	fluorescence intensity, a.u.
1	665.1
2	663.2
3	662.4
4	665.2
5	663.5
average (I_{avg})	663.9
standard deviation (σ)	1.1

The slope of the fluorescence response (m) was obtained from the best fit of the fluorescence intensity as a function of the H_2O mole fraction in a series of H_2O−D_2O mixed solutions (Figure 4b). The data were fitted using an exponential decay function with R^2 = 0.999.

$$y = a_1 \times e^{x/R_1} + y_0 \quad \text{(Eq. S1)}$$

The slope (m) of the fluorescence response in the H_2O−D_2O mixed solutions at x = 0.1 mol% H_2O (99.9 mol% of D_2O) was calculated from Eq. S1 as:

$$m = \frac{dy}{dx} \quad \text{(Eq. S2)}$$

Table S3. Parameters used for the detection limit calculation.

parameter	value
normalized standard deviation (σ_n)	0.00167
a_1	87.124
R_1	57.132
slope (m)	8.745
**detection limit, %**	**0.377**

Calculation of rigid L@MIL-53-$(OH)_2$ production cost at laboratory and industrial scale.

The cost analysis includes the costs of the starting chemicals, reaction energy, and filtration/separation step used for isolation and washing of the MOF where chemicals are purchased as mentioned in supporting manuscript. For the industrial-scale estimation, the projected production cost is based on linear scale-up of the experimentally demonstrated synthesis focusing on the required quantities of the raw materials and the corresponding large-scale filtration process per kg scale. The labour cost and instrument cost has not been considered for industrial scale synthesis.[8] The laboratory scale synthesis include $In(NO_3)_3{\cdot}xH_2O$ from sigma ($98.28 for 10 g) and H_2BDC-$(OH)_2$ from TCI ($301 for 25 g). For the industrial scale the purchase cost is predicted from bulk vendors such as Alibaba which shows H_2BDC-$(OH)_2$ cost to be $8.45/kg (Jinan Diamond Chemical Co. Ltd.). No vendors were found for $In(NO_3)_3{\cdot}xH_2O$ and it is predicted that $InCl_3$ can be used for similar synthesis with similar yield where the cost of $InCl_3$ was $29.56/kg (Hangzhou Baosyn Chemical Co. Ltd.). The detailed cost calculation, including the raw-material costs, filtration costs, and the estimated total production cost at both scales, has now been added to the Supporting Information (Table S4).

Table S4. Parameter breakdown and cost analysis for the production of **rigid L@MIL-53-$(OH)_2$** at laboratory and industrial scales.

parameter	laboratory scale	industrial scale (kg)
MOF produced	0.114 g	1 kg
Indium(III) source	0.0225 g	1.45 kg
H_2BDC-$(OH)_2$	0.099 g	0.868 kg
water	60 mL	526.3 L
Indium(III) source cost	$2.21	$42.86
H_2BDC-$(OH)_2$ cost	$1.24	$7.33
water cost	$0.05	$3
filtration cost	$0.20	$15
energy cost	$0.20	$5
estimated total	$3.90/batch	
**cost per gram**	**$34.21/g**	**$73.19/kg ($0.07/g)**

X-ray crystal structure determination of rigid L@MIL-53-$(OH)_2$.

A single crystal of rigid L@MIL-53-$(OH)_2$ was coated with Paratone oil. The diffraction data were measured at 298 K with synchrotron radiation (λ = 0.700 Å) on an ADSC Quantum-210 detector at 2D SMC with a silicon (111) double crystal monochromator at the Pohang Accelerator Laboratory, Korea. The ADSC Q210 ADX software[9] was used for data collection and HKL3000sm (ver. 703r)[10] was used for cell refinement, reduction, and absorption correction. The crystal structure was solved by a direct method using the XS program and refined by full-matrix least-squares calculations using the XL program of the SHELX program package.[11]

The diffraction data could formally be indexed and merged in either the centrosymmetric space group *C*2/*m* or its non-centrosymmetric subgroup *Cm*. However, upon refinement in *C*2/*m*, the residual electron density within the pore could not be resolved into a chemically meaningful H_2BDC-$(OH)_2$ model because the imposed crystallographic symmetry averaged the orientations, which could not be modeled in a manner consistent with the ligand composition determined by TGA and EA results. By contrast, refinement in *Cm* resolved this density as a single partially occupied H_2BDC-$(OH)_2$ ligand, yielding a chemically reasonable structural model consistent with the TGA and EA results.[12]

The asymmetric unit consists of two indium ions, two μ-OH residues, two H_2BDC-$(OH)_2$ ligands, a statistically disordered H_2BDC-$(OH)_2$ ligand with 0.5 site occupancy, and five water sites with a total site occupancy of 3.5. All non-hydrogen atoms were refined anisotropically, and the hydrogen atoms were assigned isotropic displacement coefficients (U(H) = 1.2U(C) or 1.5U(O)) allowed to ride on their respective atoms. The least-squares refinement of the structural model was performed using DFIX and ISOR for the statistically disordered H_2BDC-$(OH)_2$ ligand. Although the EA of rigid L@MIL-53-$(OH)_2$ suggested the presence of additional 0.5 water molecules and 0.6 H_2BDC-$(OH)_2$ ligands in the solvent pores per asymmetric unit, the final refinement was performed by modifying the structure factors for the electron densities of the disordered solvents (198 Å^3, 6.3% of the total unit cell volume; 147 electrons per unit cell) using the SQUEEZE option in PLATON.[13]

The symmetry reduction from *C*2/*m* to *Cm* is accompanied by the loss of the crystallographic inversion and twofold operators; macroscopically, this manifests as inversion twinning (a form of merohedral twinning), which was modeled explicitly in SHELXL using the TWIN/BASF instructions. Refinement converged to a twin (Flack) fraction of 0.6(2), which is within experimental error of 0.5. A twin fraction approaching 0.5 represents the theoretical limit at which the two twin domains contribute statistically indistinguishable, fully overlapping intensities; if the twin fraction equals 0.5, the crystal is perfectly twinned and the intensity measurements cannot be deconvoluted regardless of data quality or refinement strategy.[14] Under these exact overlapping conditions, elevated R_1 = 0.1494, wR_2 = 0.4751, and GOF on F^2 = 1.872 values, together with residual electron-density features near the heaviest scatterers (The largest difference peak and hole were 6.016 and −3.776 e·Å^{-3}, respectively), are largely attributable to the near-1:1 inversion twinning rather than an indication of an incorrect structural model.[15]

The pore-confined guest species (isolated O atoms O1W–O5W) represent highly mobile guest water molecules. Due to their severe dynamic disorder within the pores and the heavily smeared electron density caused by the 1:1 inversion twinning, hydrogen-atom positions could not be located with confidence from the difference Fourier map and were therefore left unrefined.

Table S5. X-ray crystal structure refinement data for **rigid L@MIL-53-(OH)$_2$**.

compound	**L@MIL-53-(OH)$_2$**
formula	$C_{20}H_{20}O_{20.5}In_2$
FW, g·mol^{-1}	818.00
T, K	298(2)
wavelength, Å	0.700
crystal system	Monoclinic
space group	*Cm*
Z	4
a, Å	14.004(3)
b, Å	18.504(4)
c, Å	13.693(3)
α, °	90
β, °	117.68(3)
γ, °	90
V, Å^3	3142.2(13)
d_{calc}, g cm^{-3}	1.729
μ, mm^{-1}	1.467
F(000)	1608
crystal size, mm^3	0.035 × 0.035 × 0.035
2 theta range, °	3.308 to 67.412
index ranges	$-20 \le h \le 20$<br>$-24 \le k \le 24$<br>$-19 \le l \le 19$
reflections collected	16379
data/restraints/parameters	9780/26/477
GOF on F^2	1.872
largest peak/hole, e/Å^3	6.016/−3.776
R_1 (wR_2), [I ≥ 2sigma(I)]	0.4751 (0.5124)

Table S6. Hydrogen bonds and close contacts between the framework and encapsulated H_2BDC-$(OH)_2$/water molecules.

atom (D)	atom (H)	atom (A)	bond length (D–H), Å	Hydrogen bond length (H···A), Å	distance (D···A), Å	angle (DHA), °
O4M	H4M	O6F[a]	0.93	1.88	2.80(3)	173.1
O2F		O1W			2.64(2)	
O3F		O2W			2.66(3)	
O4F		O3W			2.62(3)	
O5	H5	O1W[b]	0.82	2.23	3.04(2)	167.7
O2M	H2M	O3W	0.93	2.03	2.93(3)	163.7
O1W		O15			2.83(3)	
O1W		O2W			2.76(3)	
O2W		O3W			2.75(3)	
O1M	H1M	O4W	0.93	1.83	2.74(2)	164.5
O3M	H3M	O5W	0.93	1.74	2.66(3)	171.6

[a]x−1/2,−y+1/2,z−1; [b]x−1/2,y−1/2,z

Table S7. π-π Stacking interactions between the framework ligand and encapsulated ligand H_2BDC-$(OH)_2$.

atom (FL)	atom (EL)	close contact distance, Å	Δ*D*, Å
O4	C5F	3.030	−0.190
C4	C4F	3.314	−0.086
C16	C7F	3.371	−0.029
C3	O3F	3.216	−0.004

FL: framework ligand BDC-$(OH)_2$; EL: encapsulated ligand H_2BDC-$(OH)_2$
Δ*D*: the close contact distance between the framework atom and encapsulated ligand atom minus the sum of the van der Waals radii of the close contact atoms.

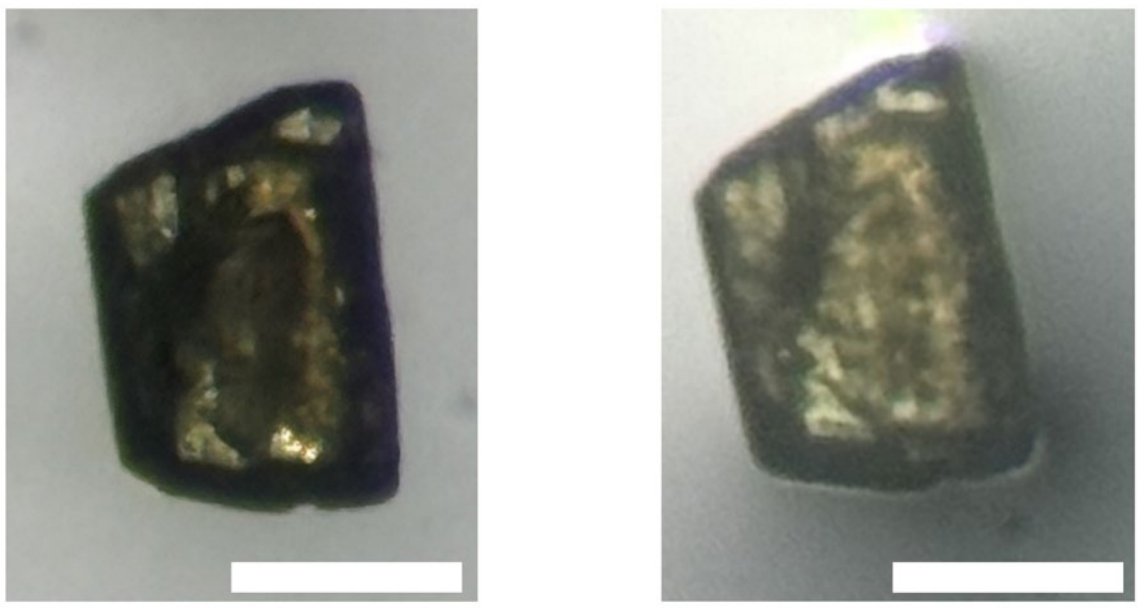

Figure S1. Microscope images of **rigid L@MIL-53-(OH)$_2$**: (*left*) dried state (*right*) after resoaking in H_2O of air-dried **rigid L@MIL-53-(OH)$_2$**. Scale bar represents 5 μm.

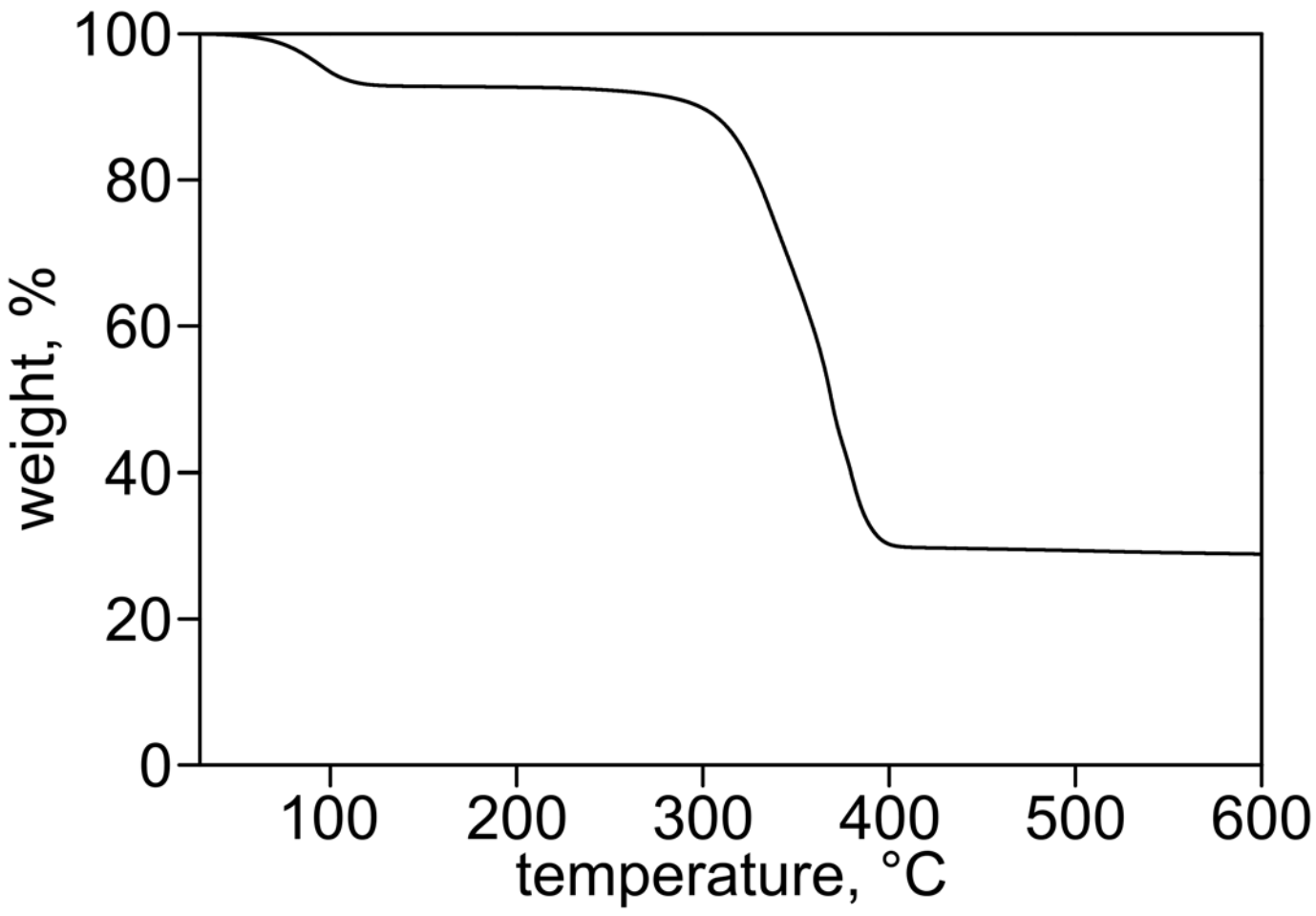

Figure S2. TGA of **rigid L@MIL-53-(OH)$_2$**. The formula is $[In(OH)((BDC)\text{-}(OH)_2)]\cdot(H_2BDC\text{-}(OH)_2)_{0.60}\cdot(H_2O)_{2.0}$.

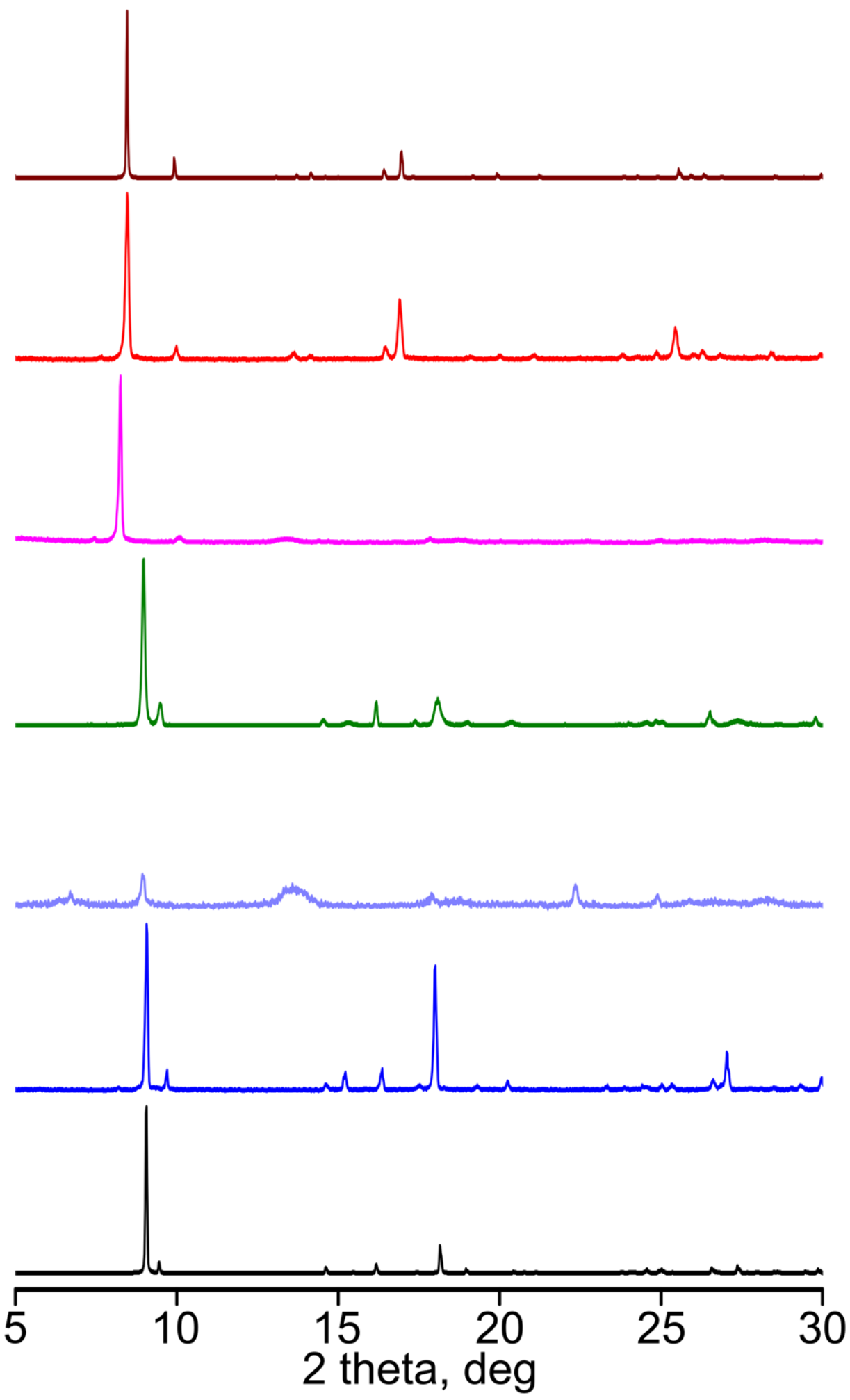

Figure S3. PXRD patterns of flexible MIL-53-$(OH)_2$: simulated of as-synthesized (black),[2] as-synthesized (blue), after air drying (light blue), soaking in, acetone (green), MeOH (pink), and H_2O (red), and simulated of H_2O (dark red)[2].

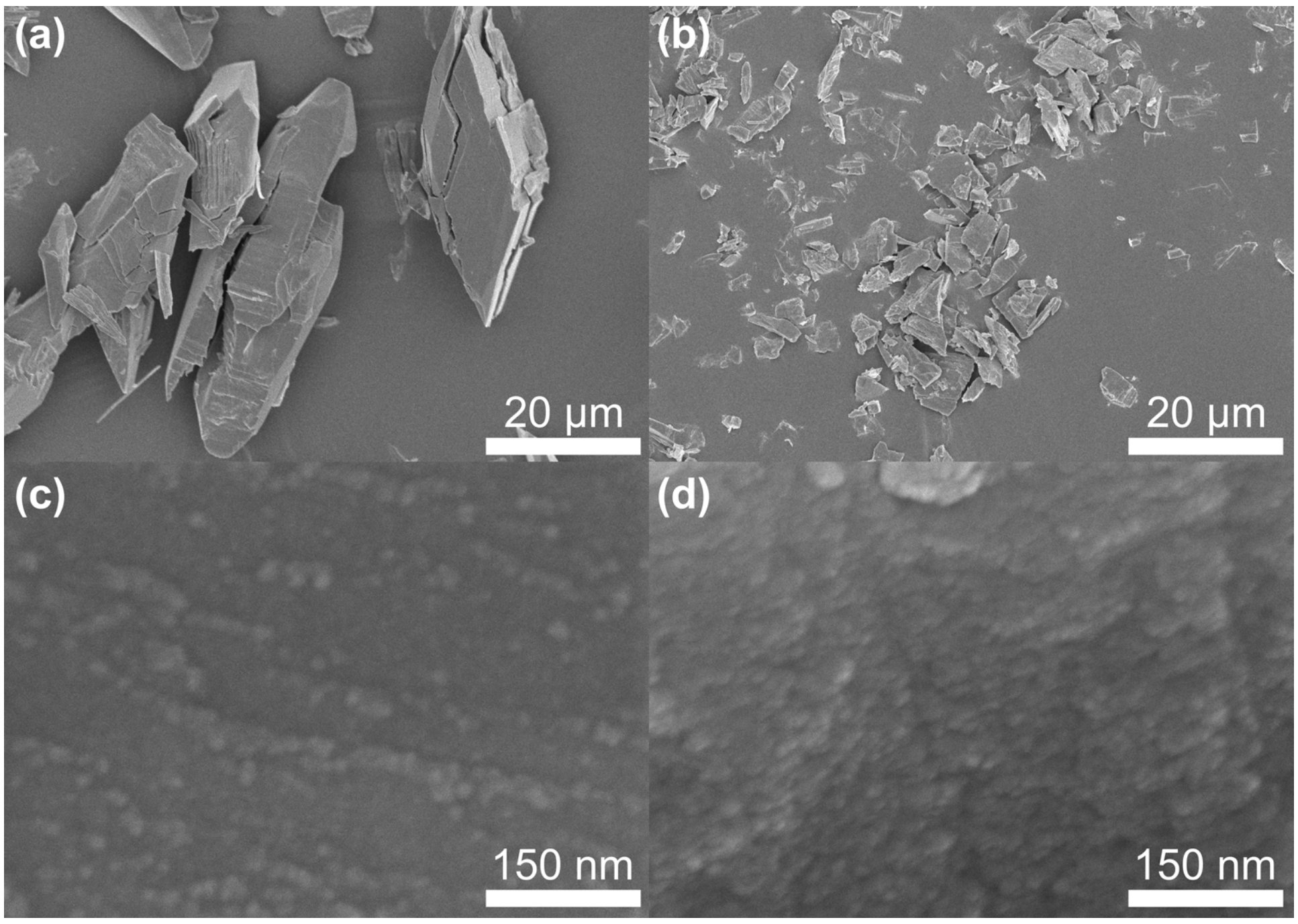

Figure S4. SEM images of flexible MIL-53-$(OH)_2$: (a, c) before soaking in H_2O and (b, d) after soaking in H_2O.

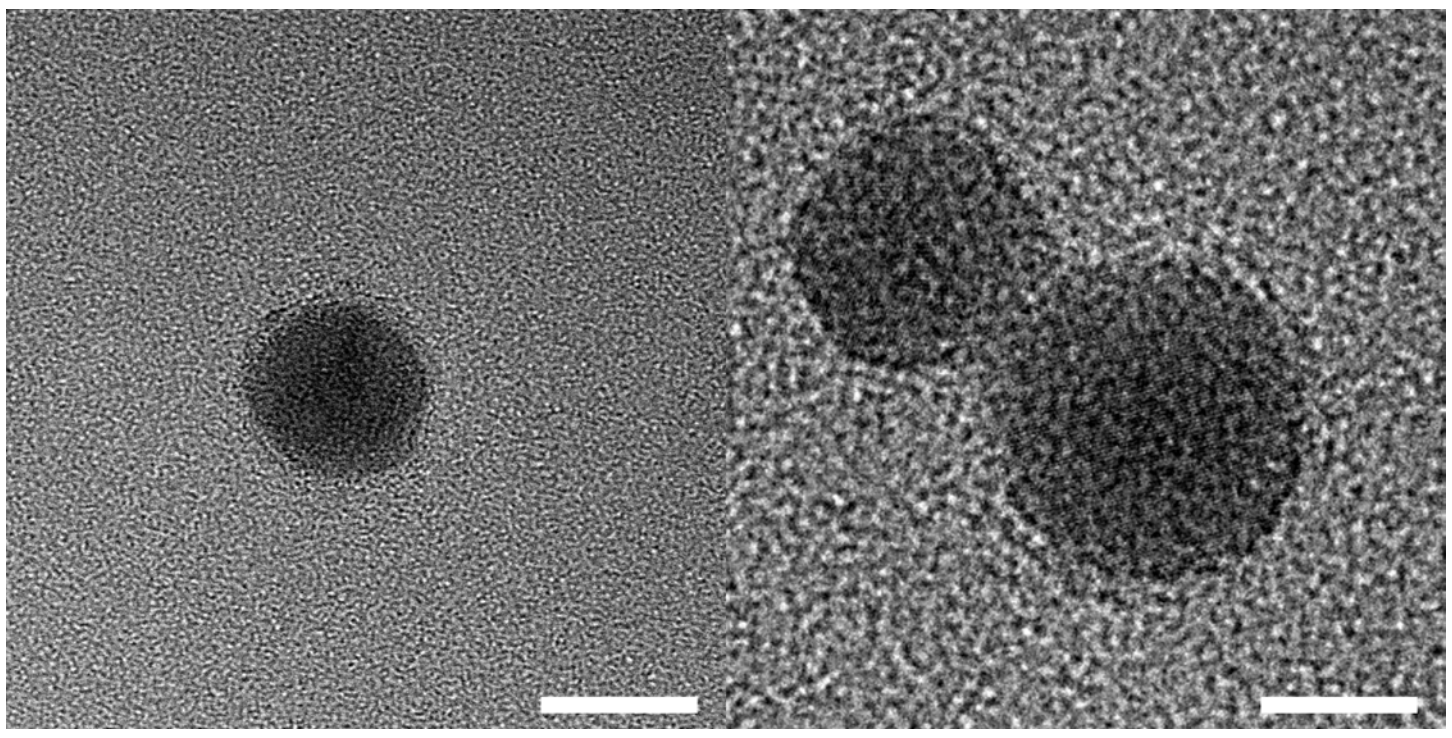

Figure S5. TEM images of dried filtrate after soaking flexible MIL-53-$(OH)_2$ in H_2O. Scale bars represent 20 nm.

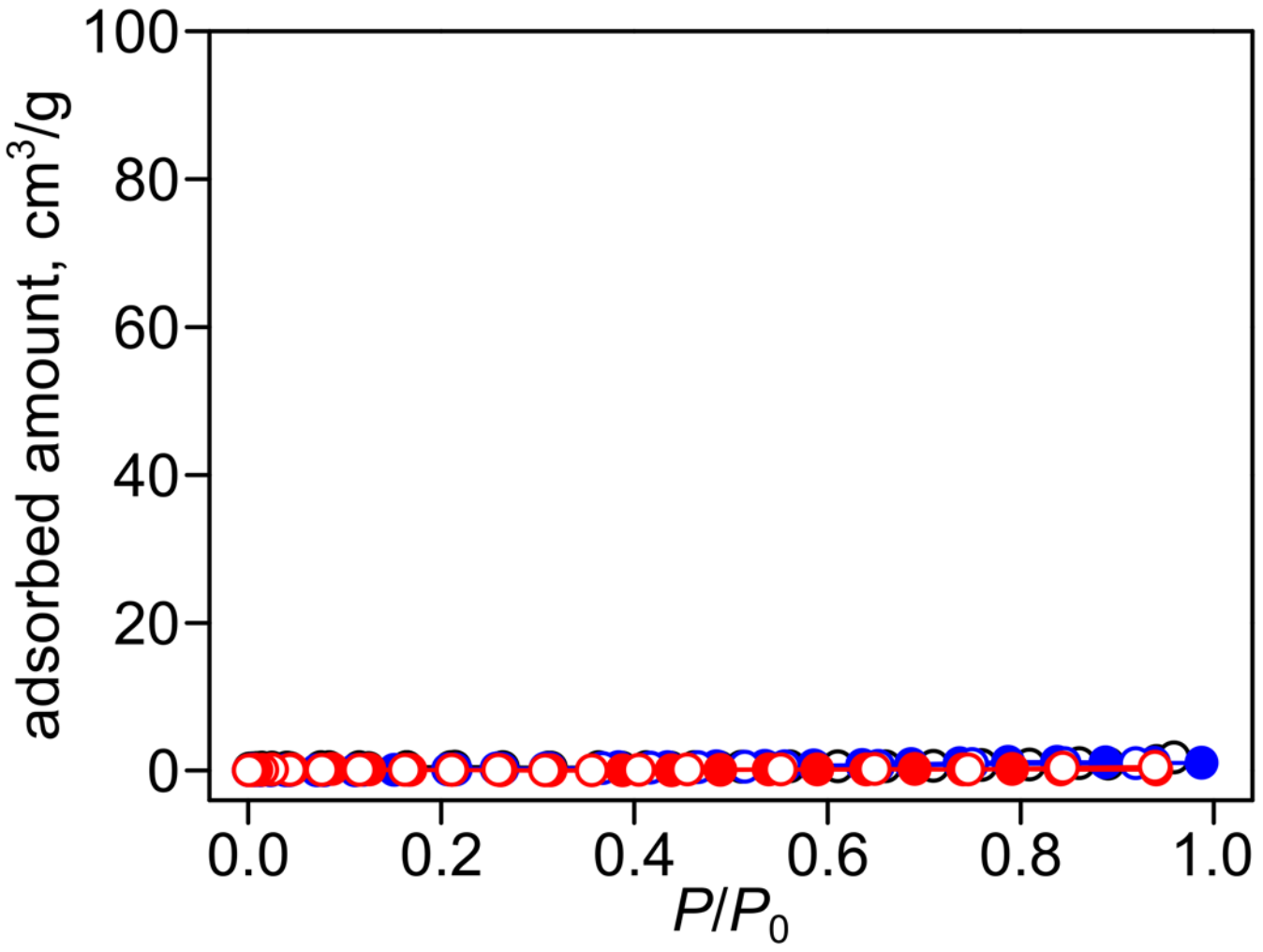

Figure S6. N_2 adsorption isotherm of flexible MIL-53-(OH)$_2$ (black), **rigid L@MIL-53-(OH)$_2$** (blue), and **rigid L@MIL-53-(OH)$_2$-150a** (red) at 77 K. Filled circles represent adsorption and empty circles represent desorption.

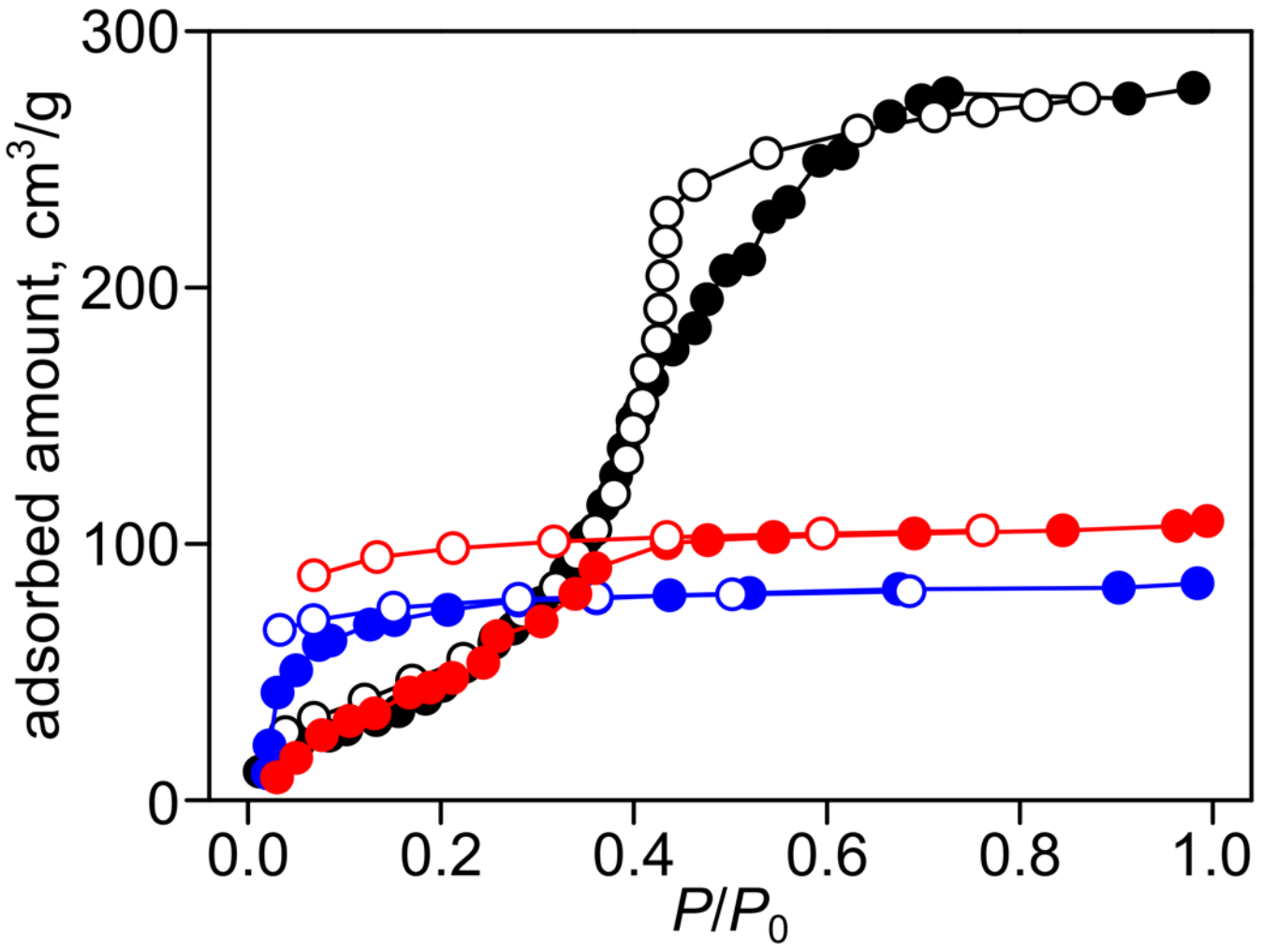

Figure S7. H_2O vapor adsorption isotherm of flexible MIL-53-(OH)$_2$ (black), **rigid L@MIL-53-(OH)$_2$** (blue), and **rigid L@MIL-53-(OH)$_2$-150a** (red) at 298 K. Filled circles represent adsorption and empty circles represent desorption.

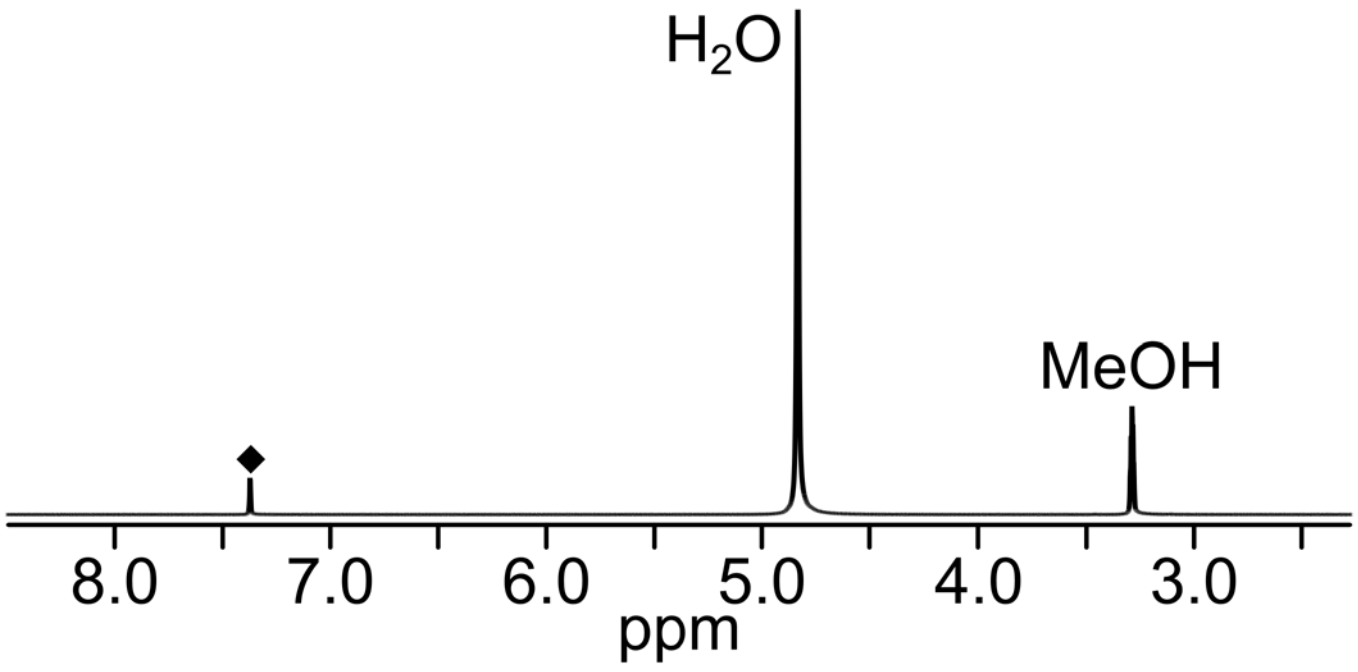

Figure S8. ^{1}H NMR spectrum of **rigid L@MIL-53-(OH)$_2$** in CD_3OD after one day at room temperature. Square represents H_2BDC-$(OH)_2$.

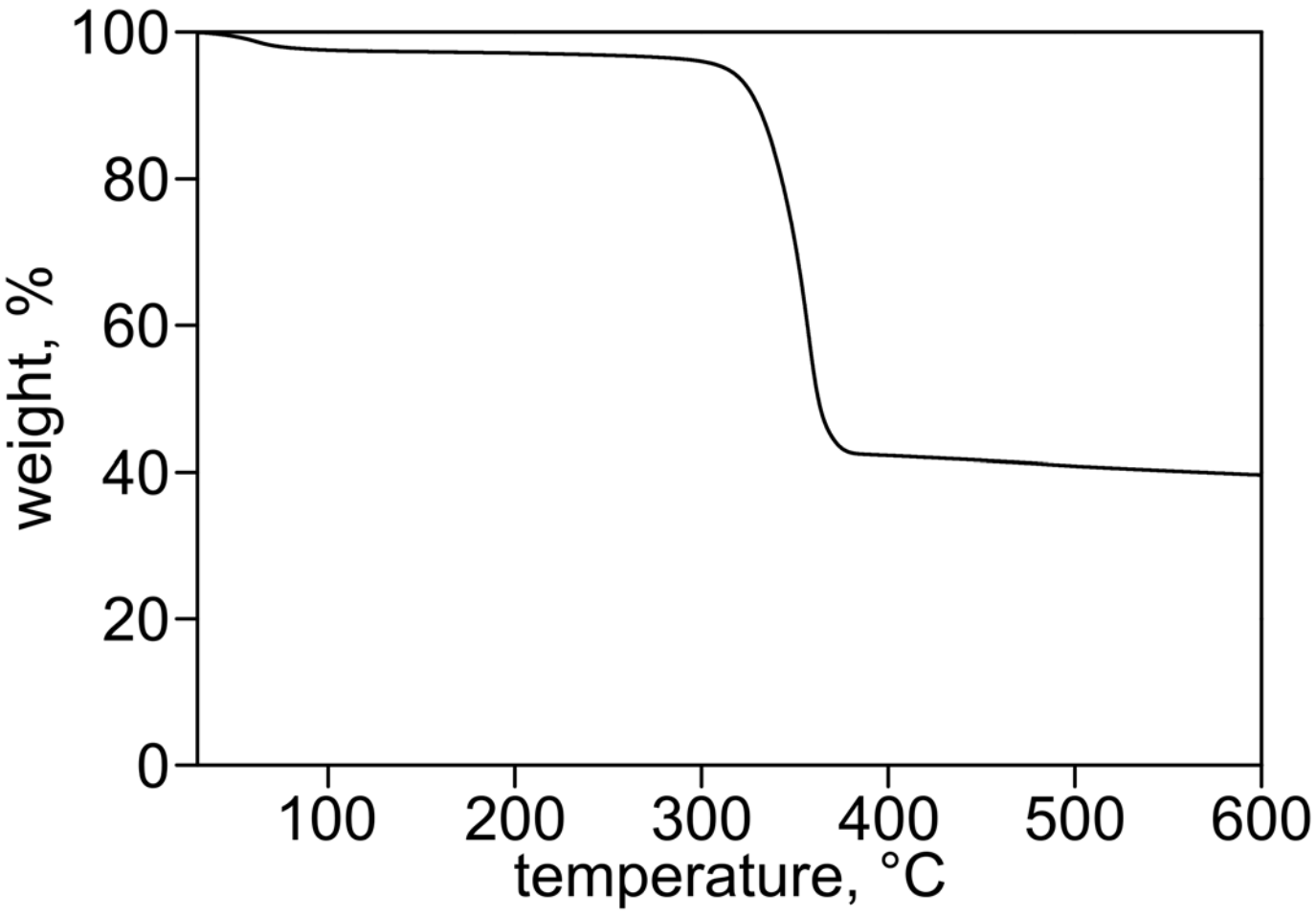

Figure S9. TGA of **rigid L@MIL-53-(OH)$_2$** after soaking in MeOH for one day at room temperature. The formula is $[In(OH)((BDC)\text{-}(OH)_2)]\cdot(H_2BDC\text{-}(OH)_2)_{0.18}\cdot(H_2O)_{1.0}$

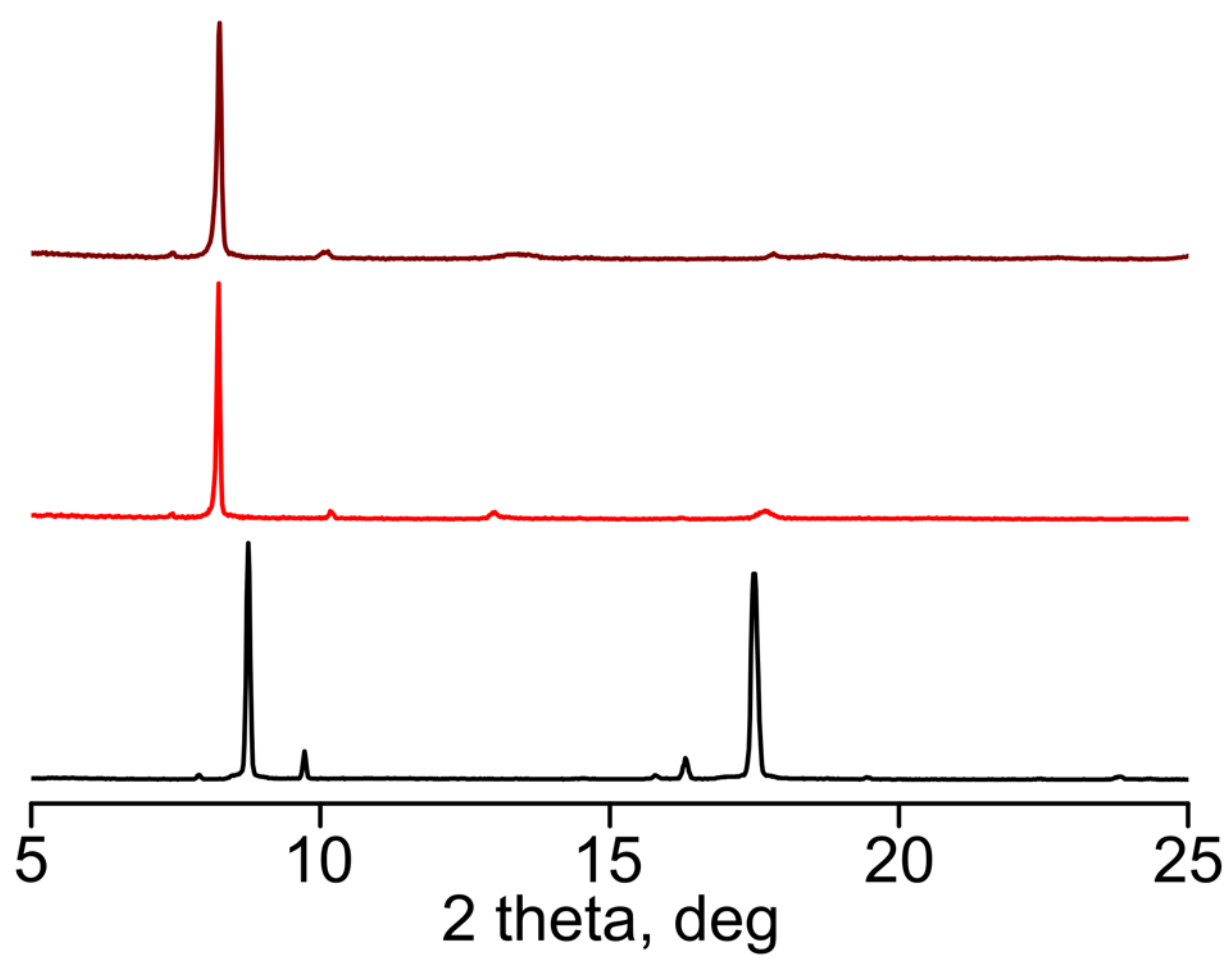

Figure S10. PXRD patterns of as-synthesized **rigid L@MIL-53-(OH)$_2$** (black), **rigid L@MIL-53-(OH)$_2$** after soaking in MeOH for one day at room temperature (red), and flexible MIL-53-(OH)$_2$ soaked in MeOH (dark red).

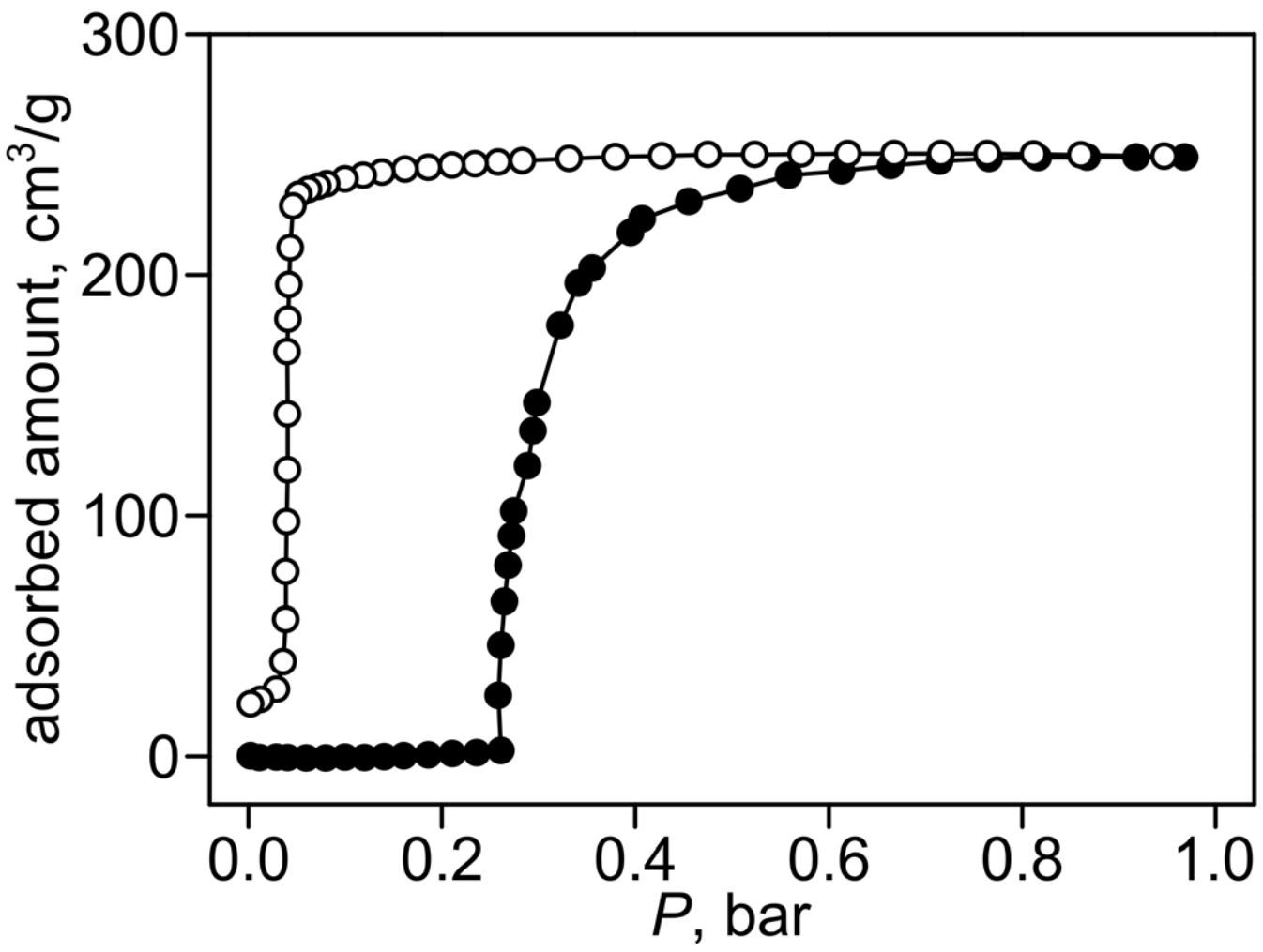

Figure S11. CO_2 adsorption isotherm of **rigid L@ MIL-53-(OH)$_2$** after soaking in MeOH at 195 K. Filled circles represent adsorption and open circles represent desorption.

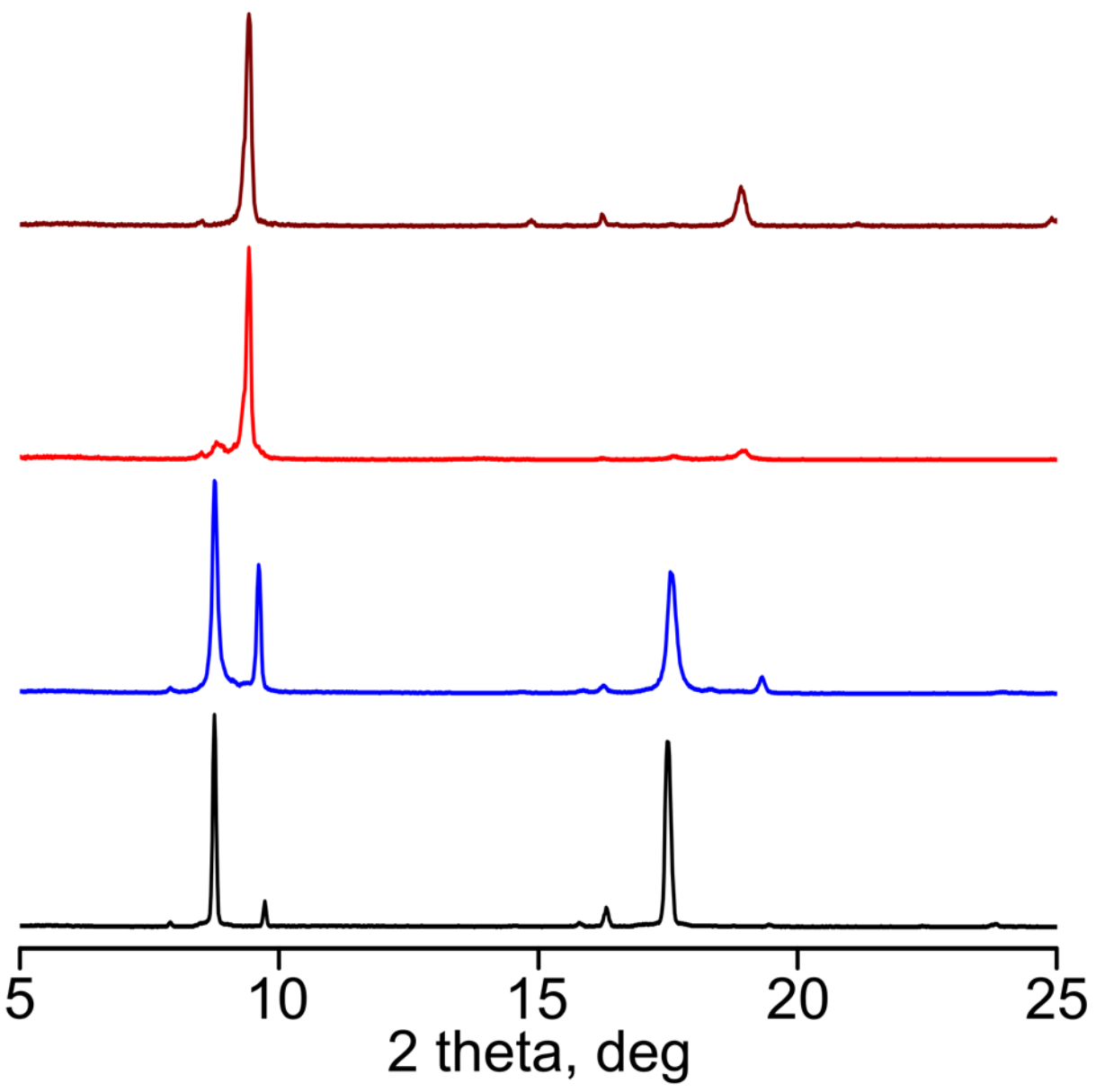

Figure S12. PXRD patterns of as-synthesized **rigid L@MIL-53-(OH)$_2$** (black), **rigid L@MIL-53-(OH)$_2$** after soaking in acetone for one day (blue) and seven days (red) at room temperature, and flexible MIL-53-(OH)$_2$ soaked in acetone (dark red).

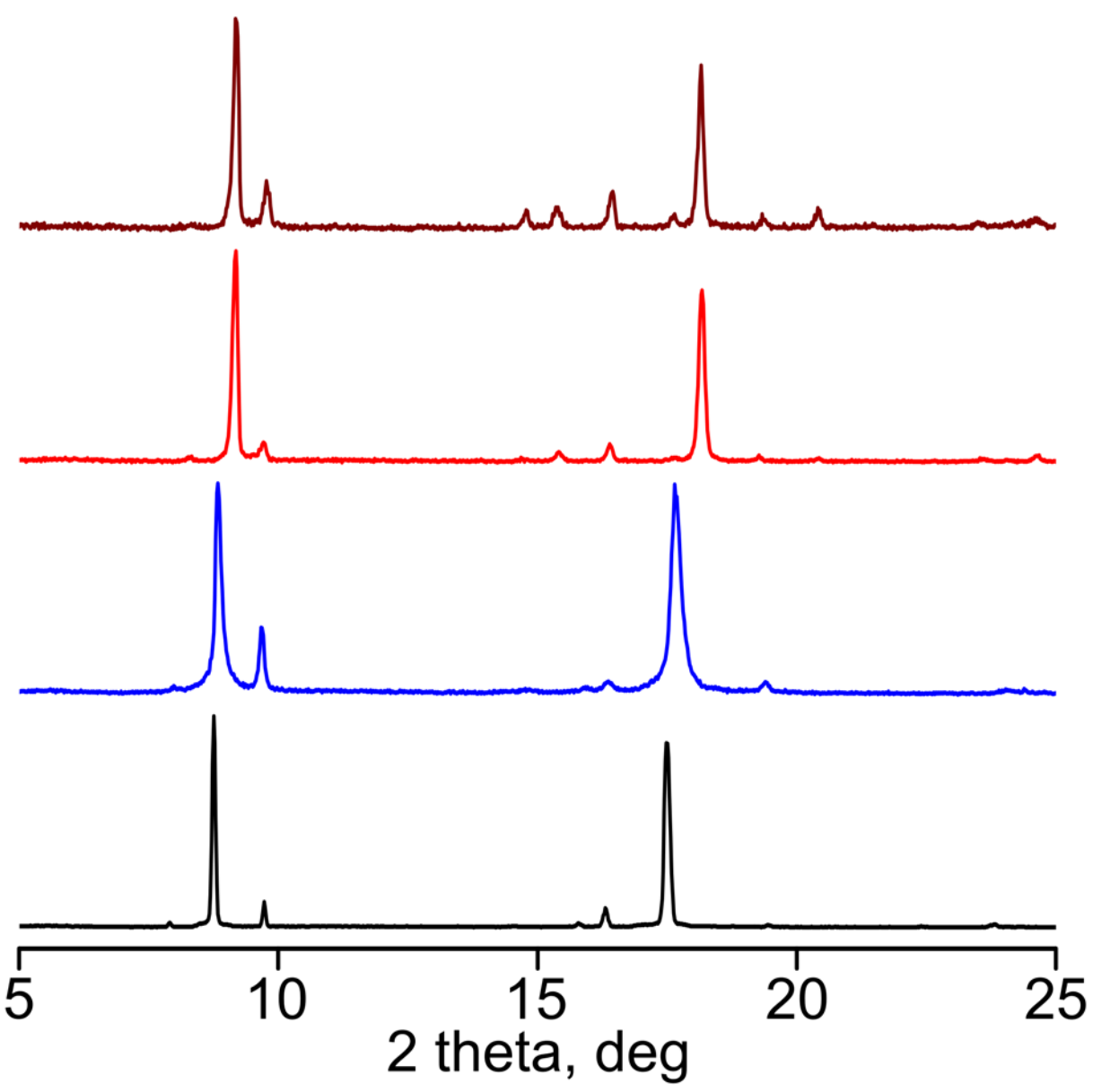

Figure S13. PXRD patterns of as-synthesized **rigid L@MIL-53-(OH)$_2$** (black), **rigid L@MIL-53-(OH)$_2$** after soaking in DMF for 14 days at room temperature (blue) and one day at 120 °C (red), and flexible MIL-53-(OH)$_2$ soaked in DMF (dark red).

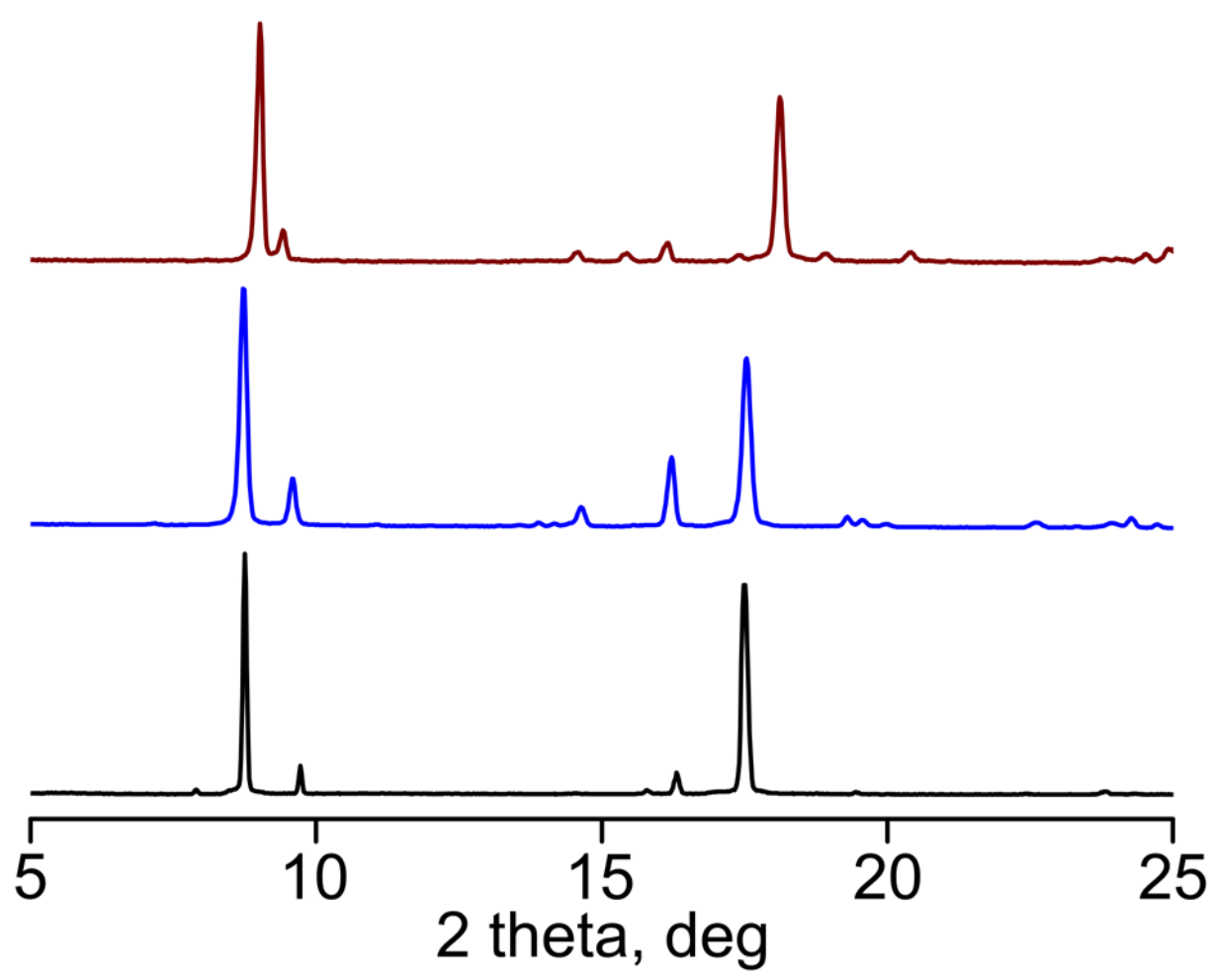

Figure S14. PXRD patterns of as-synthesized **rigid L@MIL-53-(OH)$_2$** (black), **rigid L@MIL-53-(OH)$_2$** after soaking in hexane for 14 days at room temperature (blue), and flexible MIL-53-(OH)$_2$ soaked in hexane (dark red).

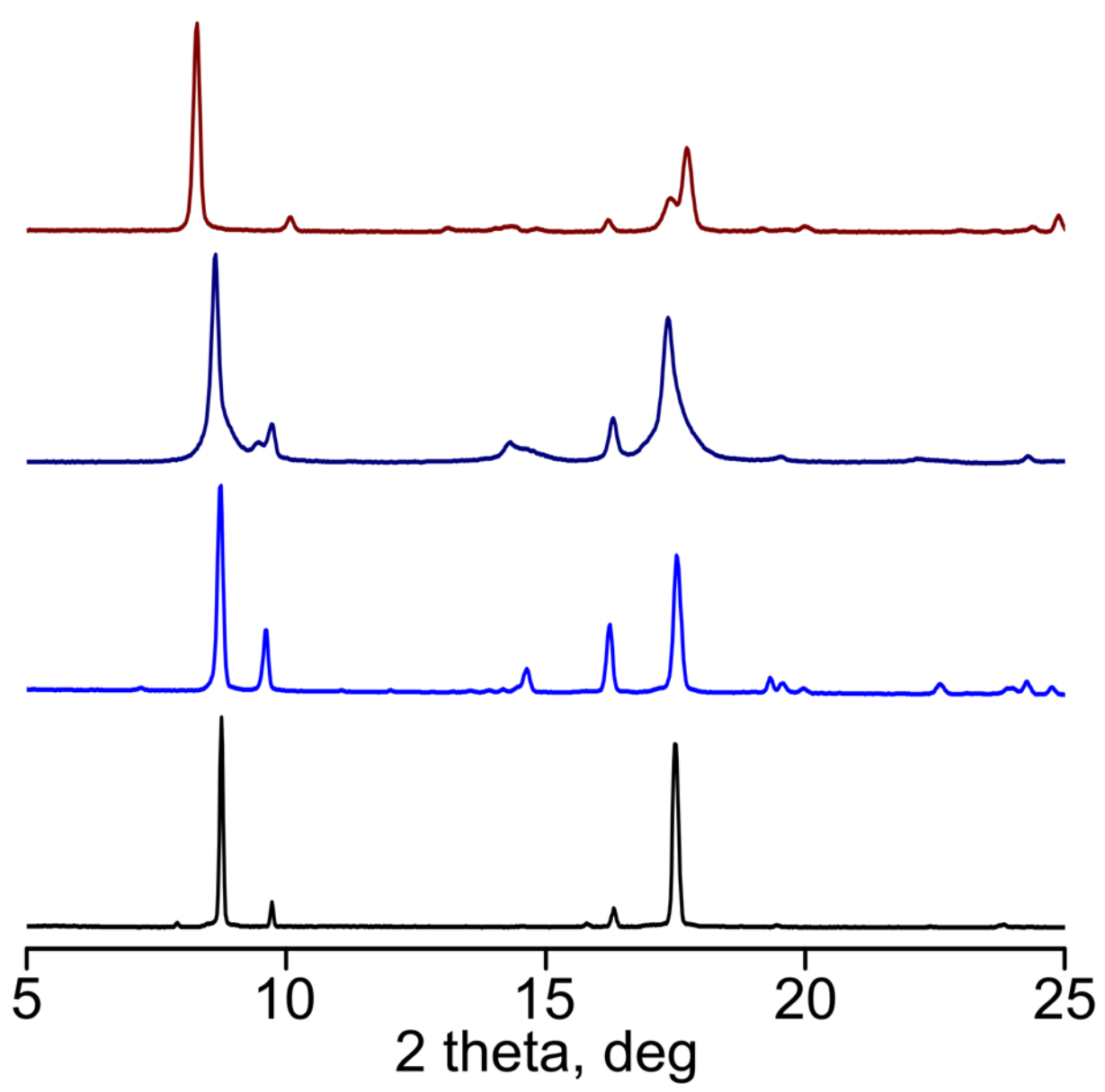

Figure S15. PXRD patterns of as-synthesized **rigid L@MIL-53-(OH)$_2$** (black), **rigid L@MIL-53-(OH)$_2$** after soaking in toluene for 14 days at room temperature (blue) and seven days at 100 °C (dark blue), and flexible MIL-53-(OH)$_2$ soaked in toluene (dark red).

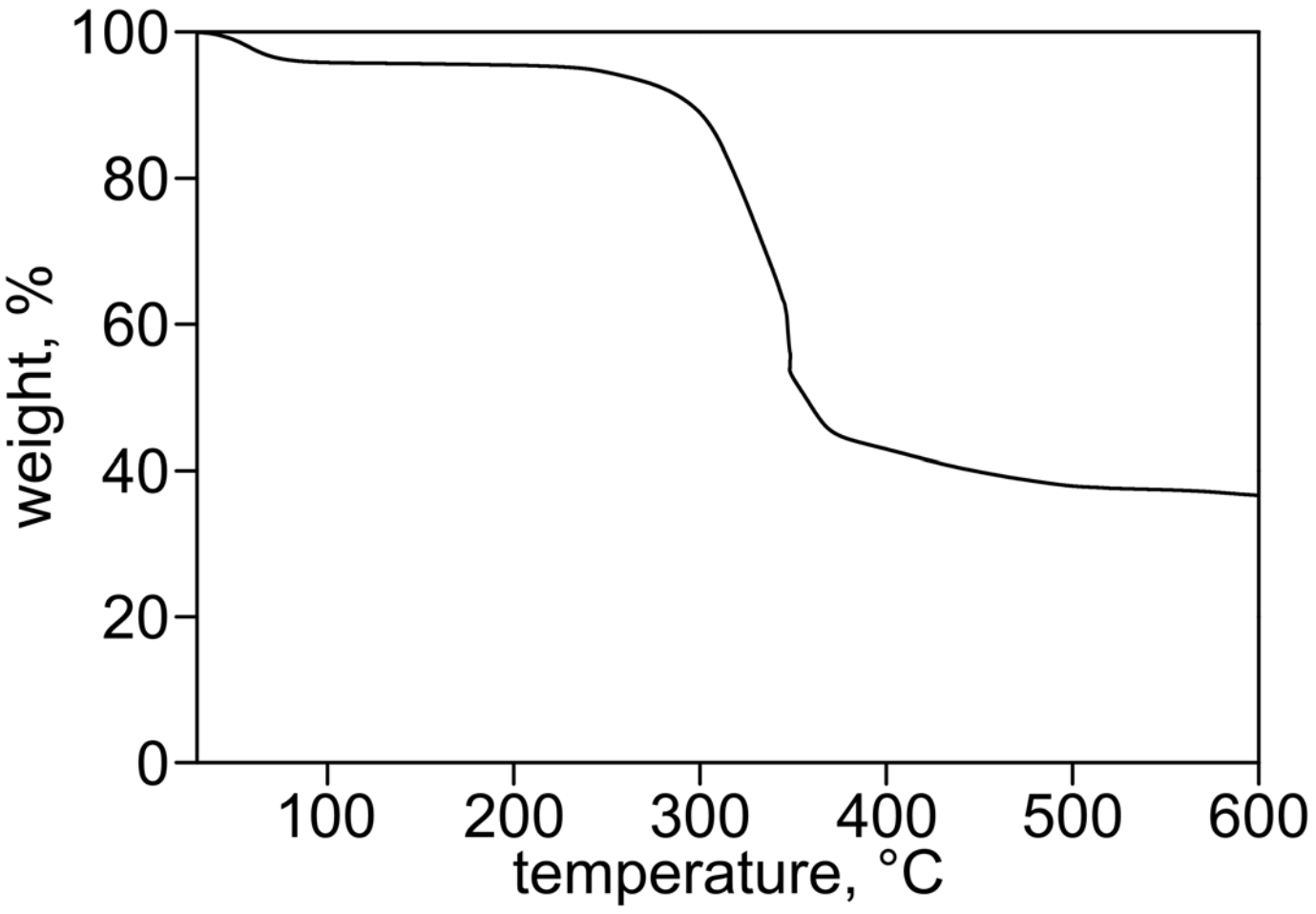

Figure S16. TGA of L@MIL-53-$(OH)_2$ prepared by post-encapsulation of H_2BDC-$(OH)_2$ into flexible MIL-53-$(OH)_2$ synthesized in H_2O. The formula is $[In(OH)((BDC)\text{-}(OH)_2)]\cdot(H_2BDC\text{-}(OH)_2)_{0.27}\cdot(H_2O)_{1.0}$.

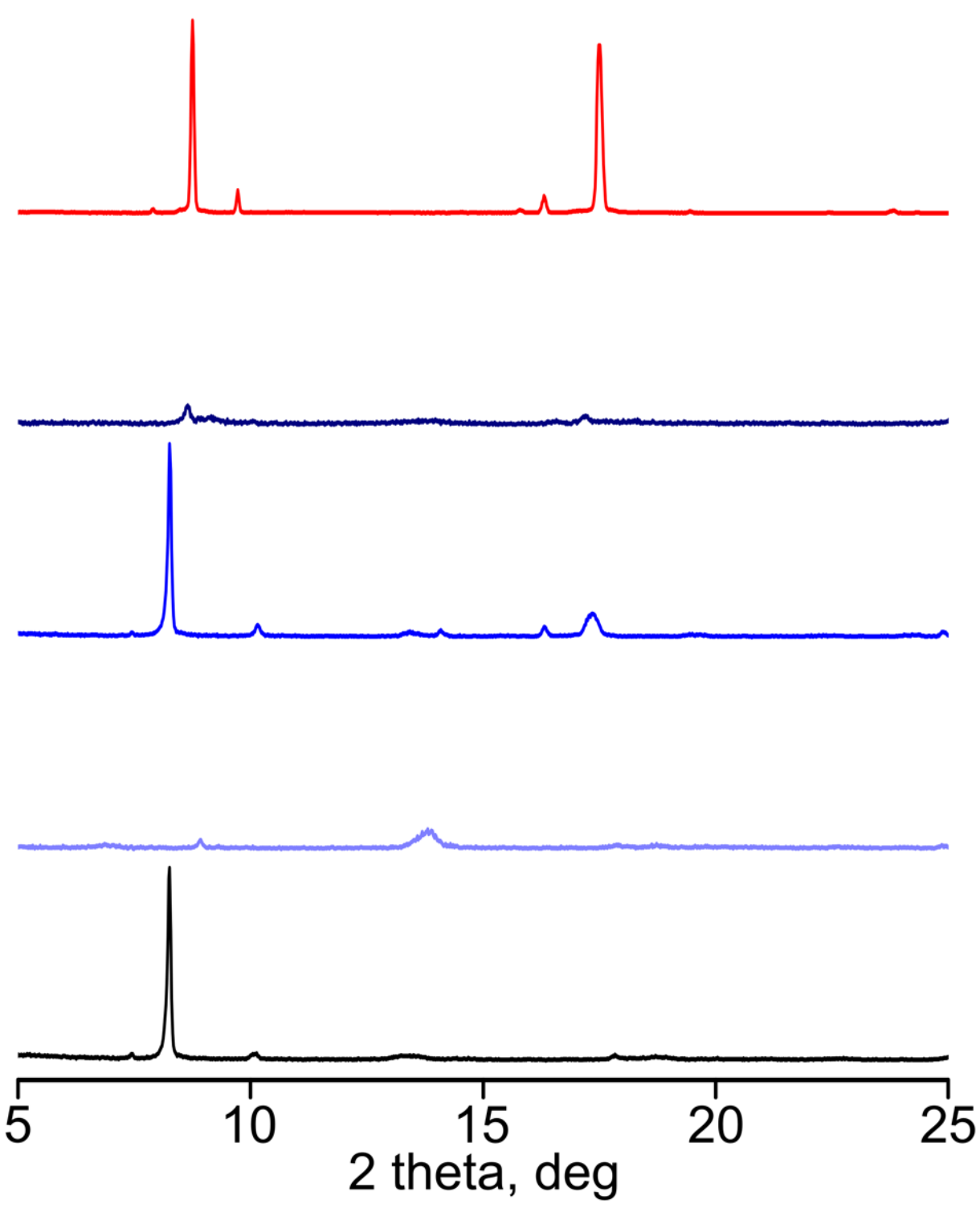

Figure S17. PXRD patterns of flexible MIL-53-$(OH)_2$ soaked in MeOH (black) and after drying (light blue); L@MIL-53-$(OH)_2$ prepared by post-encapsulation in MeOH, as-synthesized (blue) and after air-drying (dark blue), and **rigid L@MIL-53-$(OH)_2$** (red).

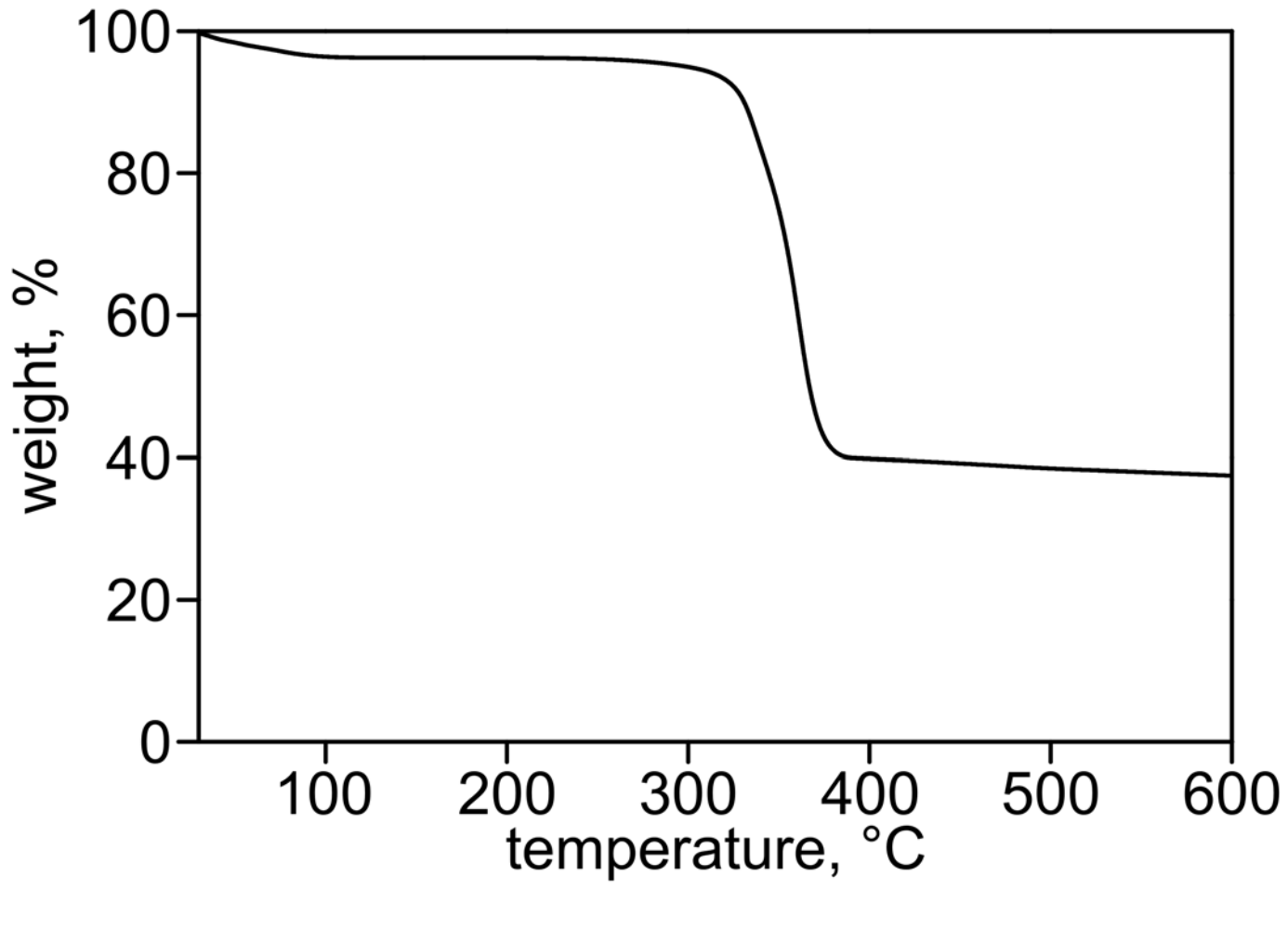

Figure S18. TGA of L@MIL-53-$(OH)_2$ prepared by post-encapsulation of H_2BDC-$(OH)_2$ into flexible MIL-53-$(OH)_2$ synthesized in MeOH. The formula is [In(OH)((BDC)-$(OH)_2$)]·(H_2BDC-$(OH)_2)_{0.25}$·$(H_2O)_{1.0}$

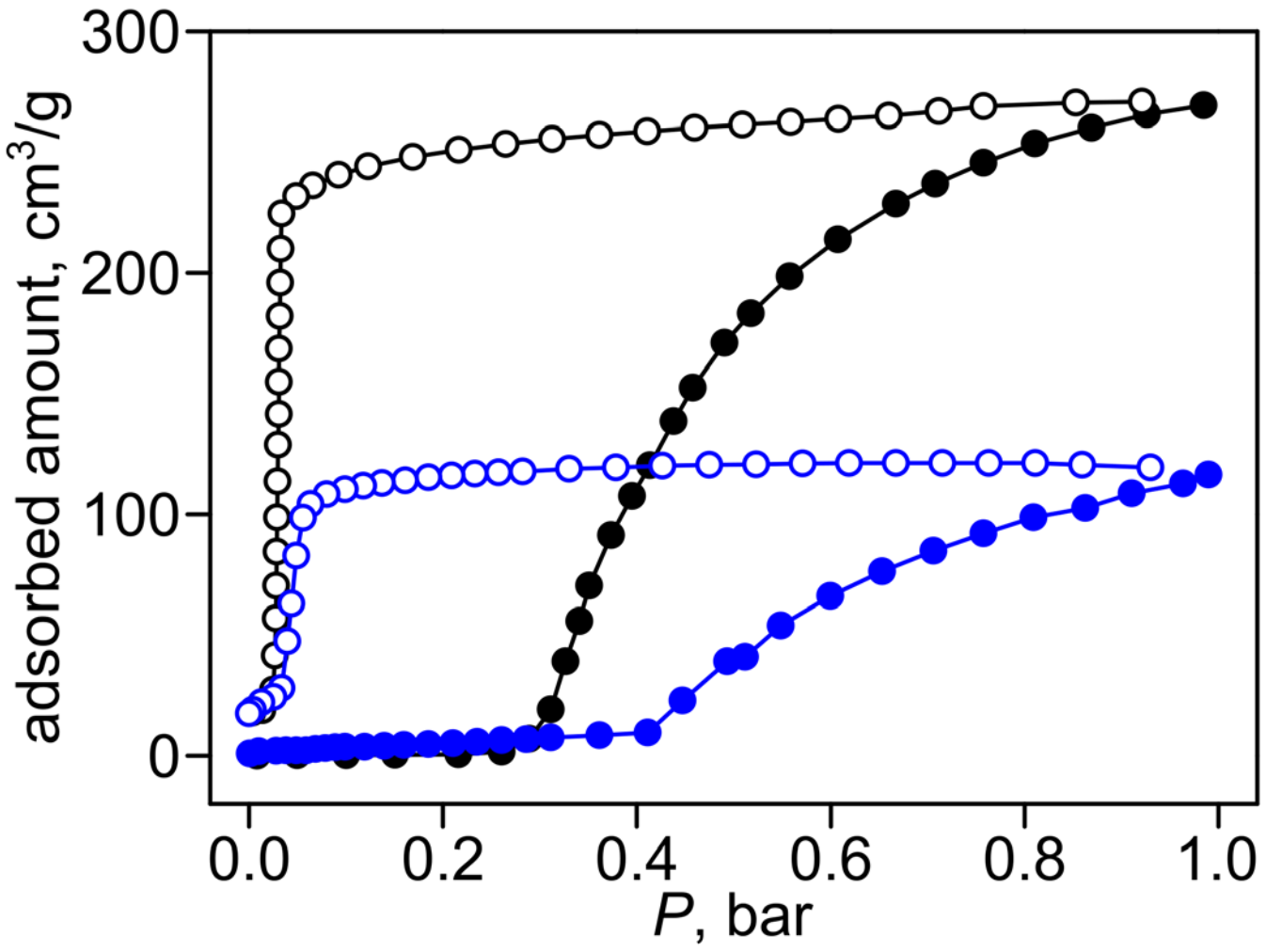

Figure S19. CO_2 adsorption isotherm of flexible MIL-53-$(OH)_2$: before (black) and after (blue) soaking in H_2O at 195 K. Filled circles represent adsorption and open circles represent desorption.

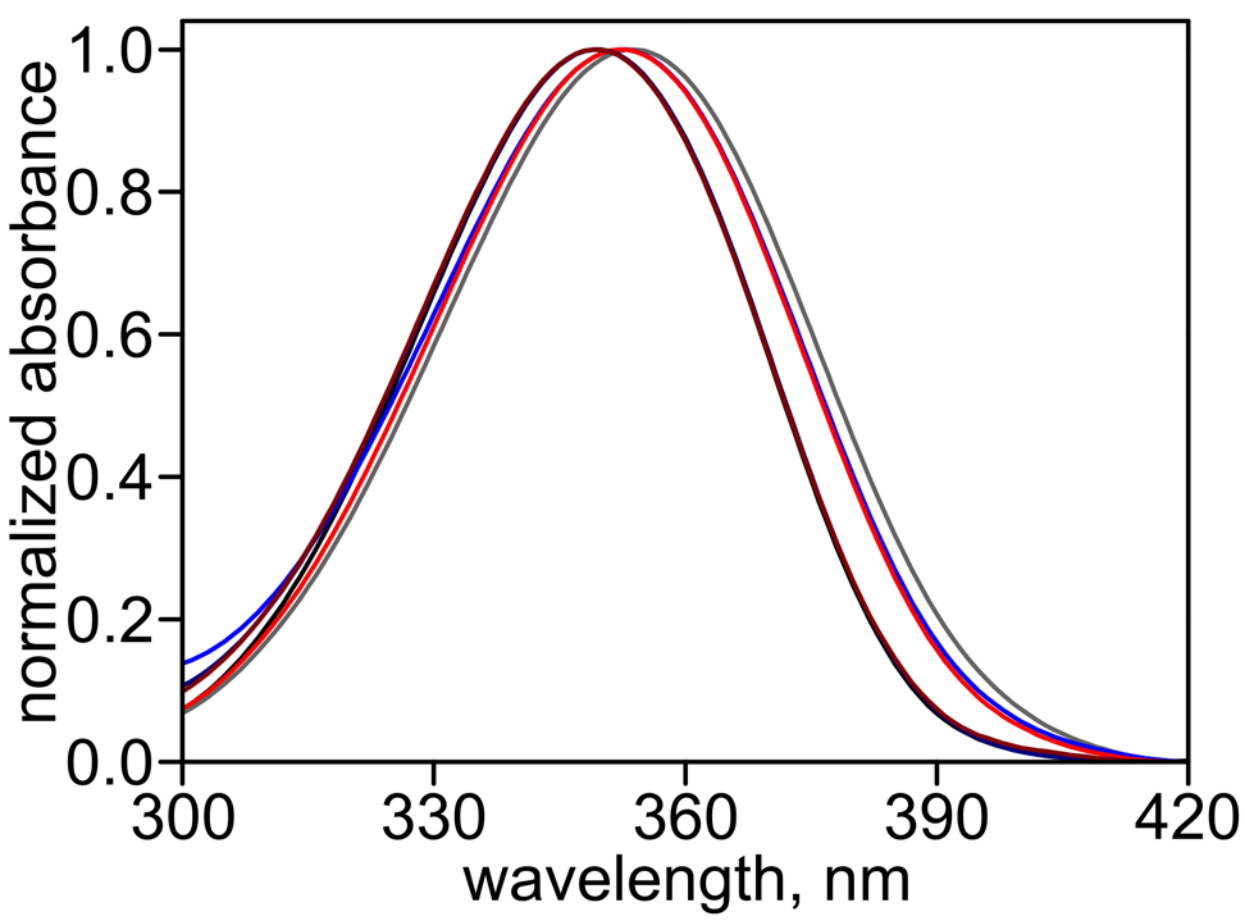

Figure S20. Normalized UV-vis spectra of H_2BDC-$(OH)_2$ in H_2O (gray), flexible MIL-53-$(OH)_2$ in H_2O (blue), and **rigid L@MIL-53-(OH)$_2$** in H_2O (red) and H_2BDC-$(OH)_2$ in D_2O (black), flexible MIL-53-$(OH)_2$ in D_2O (dark blue), and **rigid L@MIL-53-(OH)$_2$** in D_2O (dark red).

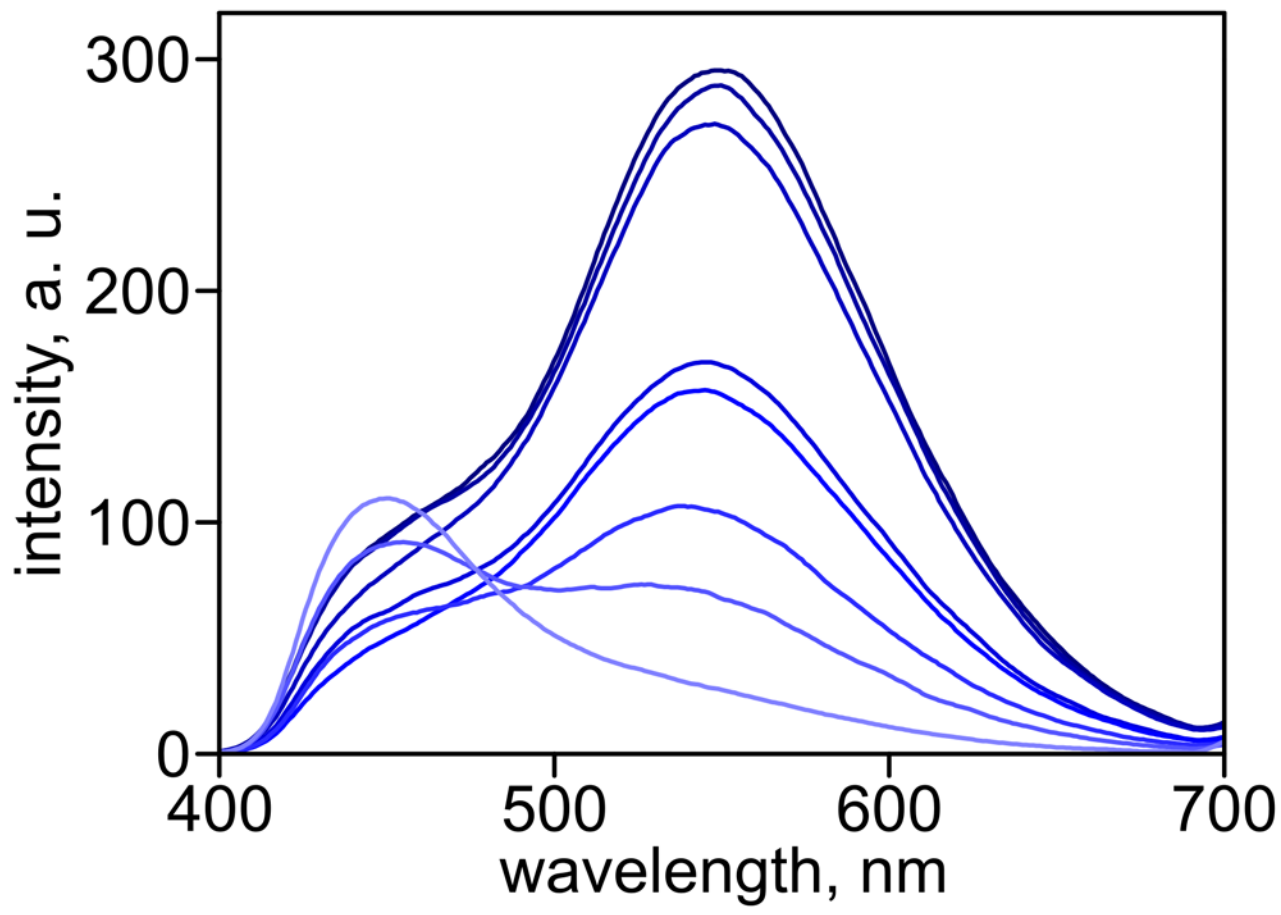

Figure S21. Fluorescence spectra of flexible MIL-53-$(OH)_2$ at varying H_2O/D_2O ratios (0.2%, 1%, 10%, 30%, 50%, 70%, 90%, and 100% H_2O), excited at 350 nm. The color changes from dark blue to light blue with increasing H_2O fraction.

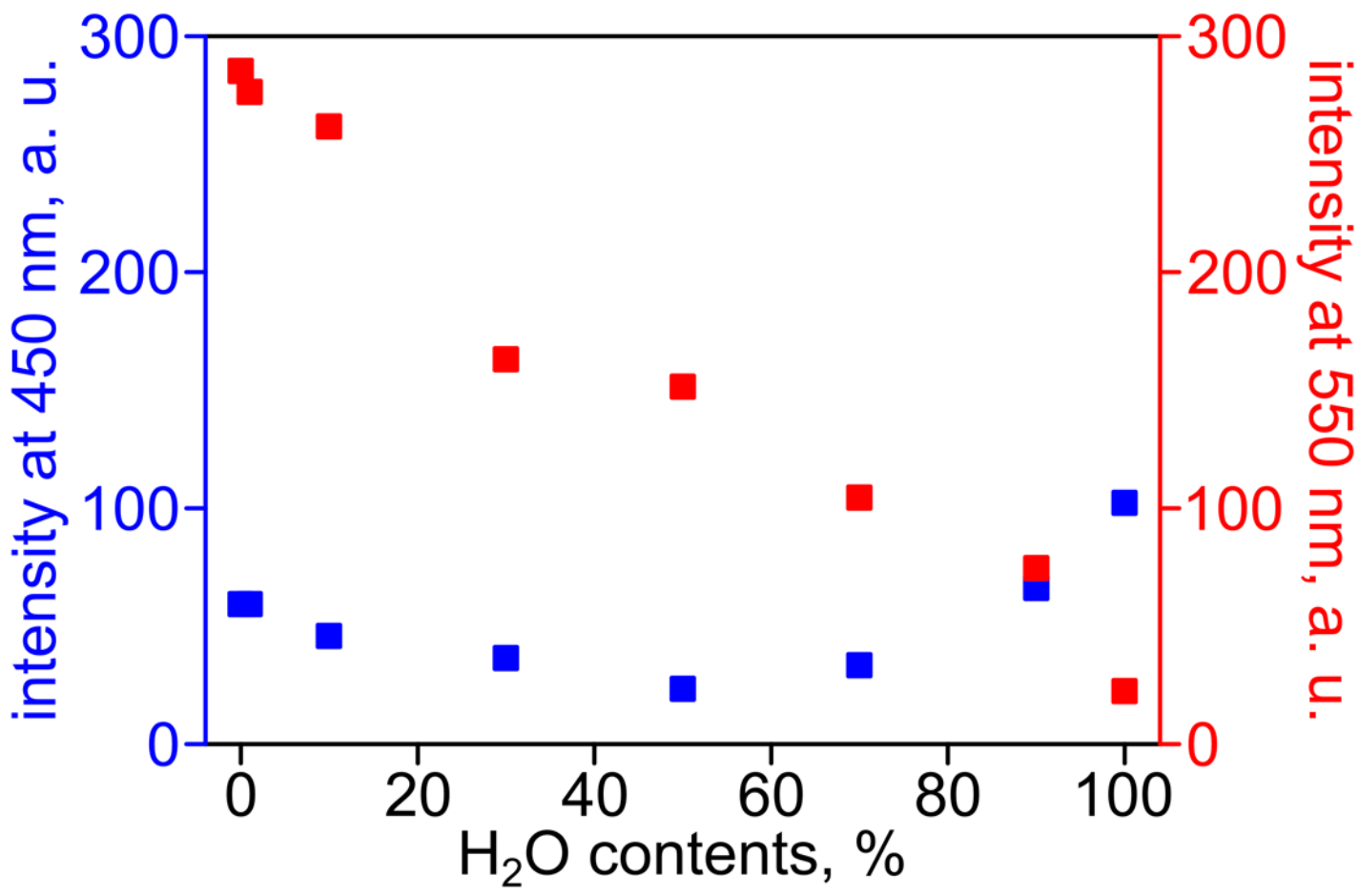

Figure S22. Fluorescence intensity of flexible MIL-53-$(OH)_2$ as a function of H_2O/D_2O ratio, measured at 450 nm (blue) and 550 nm (red) (λ_{ex} = 350 nm).

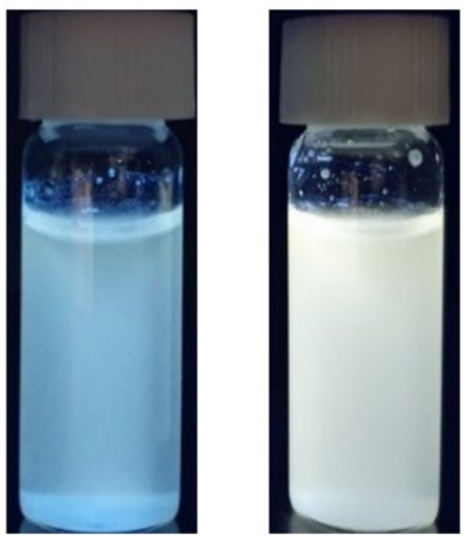

Figure S23. Emission images of flexible MIL-53-$(OH)_2$ dispersed in H_2O (*left*) and D_2O (*right*) under excitation at 365 nm.

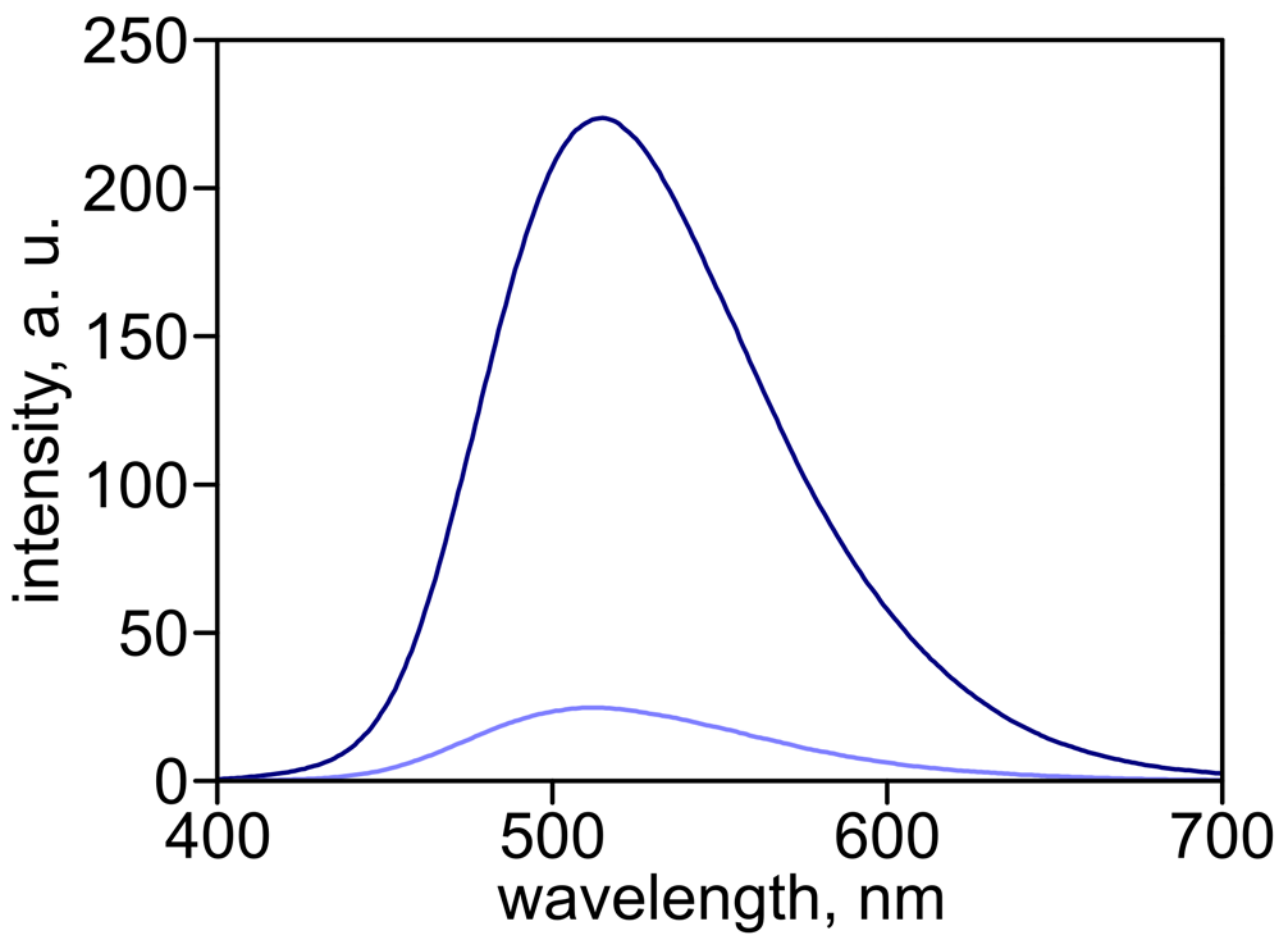

Figure S24. Fluorescence spectra of H_2BDC-$(OH)_2$ in H_2O (blue) and D_2O (dark blue) excited at 350 nm.

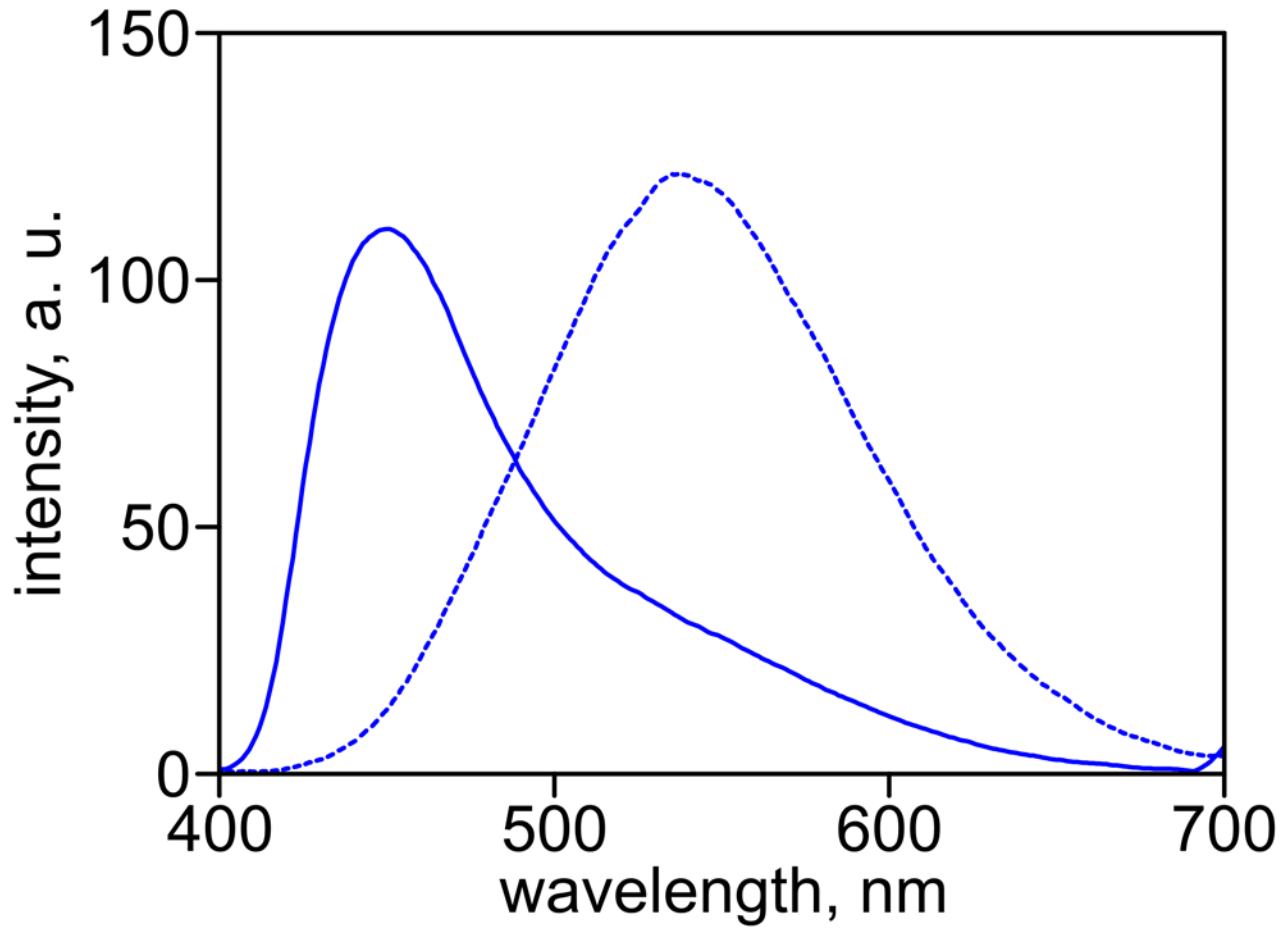

Figure S25. Fluorescence spectra of bulk flexible MIL-53-$(OH)_2$ crystals (solid line) and the corresponding filtrate after crystal removal (dashed line), following soaking in H_2O (λ_{ex} = 350 nm).

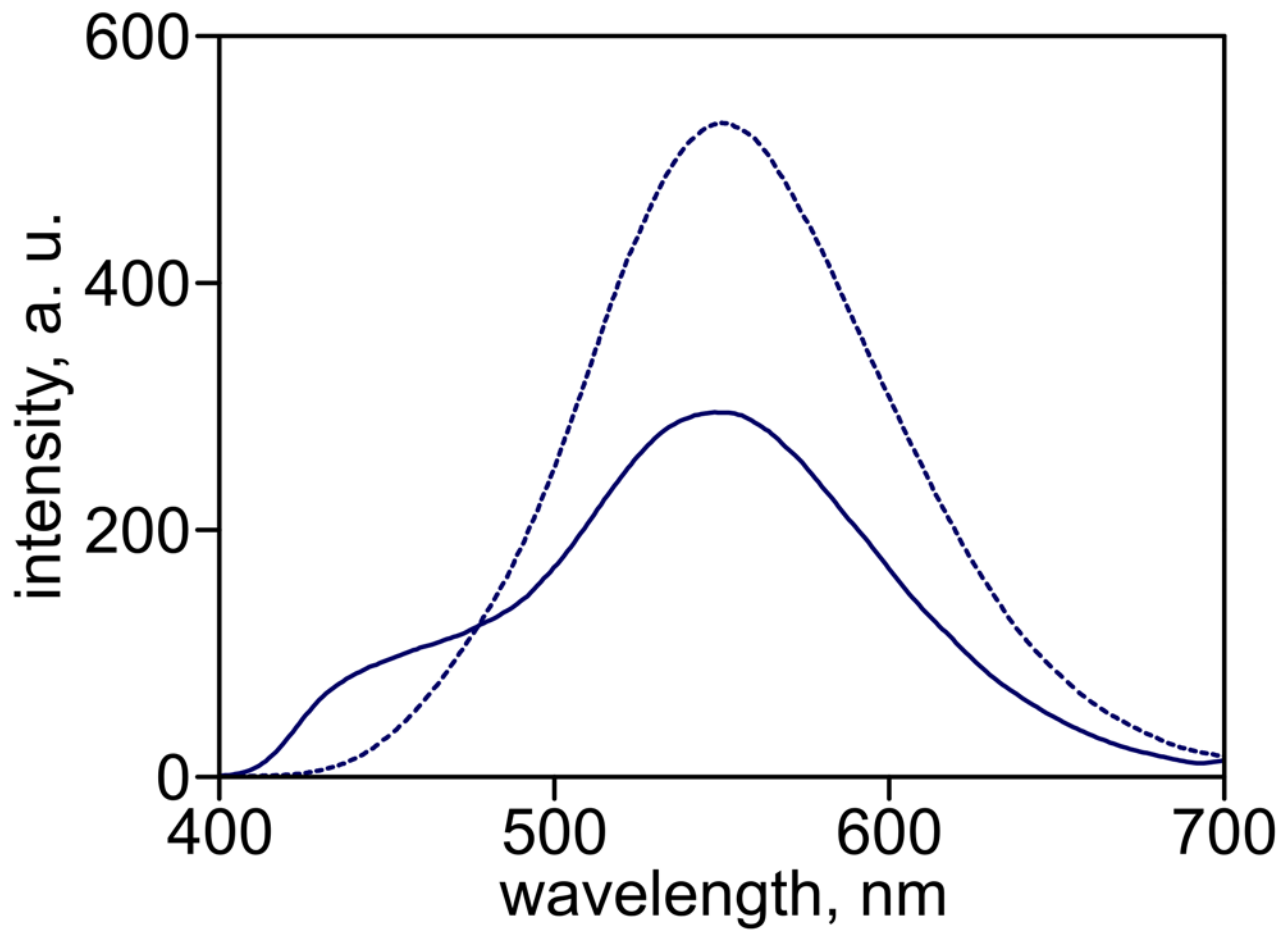

Figure S26. Fluorescence spectra of bulk flexible MIL-53-$(OH)_2$ crystals (solid line) and the corresponding filtrate after crystal removal (dashed line), following soaking in D_2O (λ_{ex} = 350 nm).

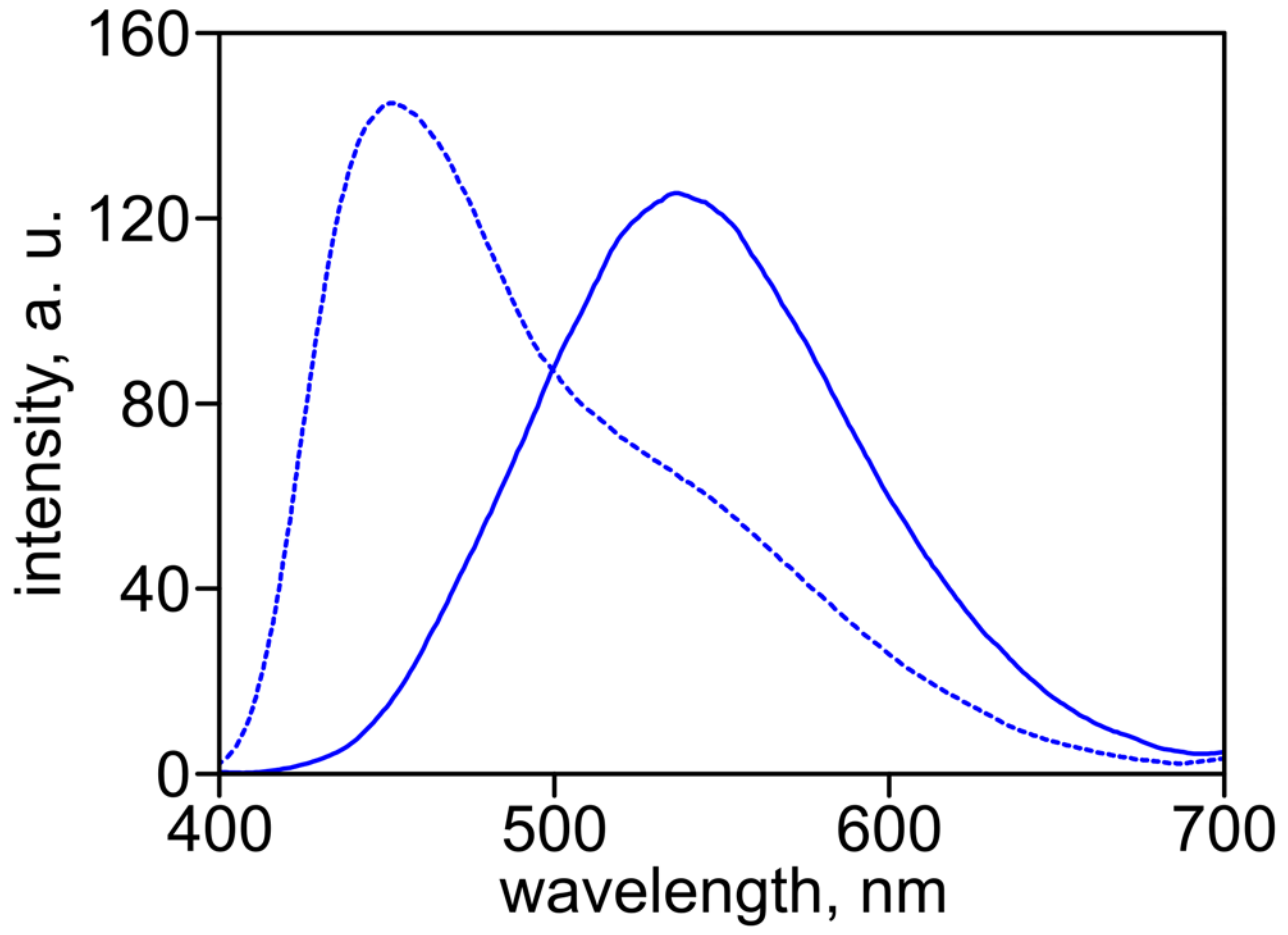

Figure S27. Fluorescence spectra of **rigid L@MIL-53-(OH)$_2$** (solid line) and after soaking in MeOH (dashed line), measured in H_2O (λ_{ex} = 350 nm).

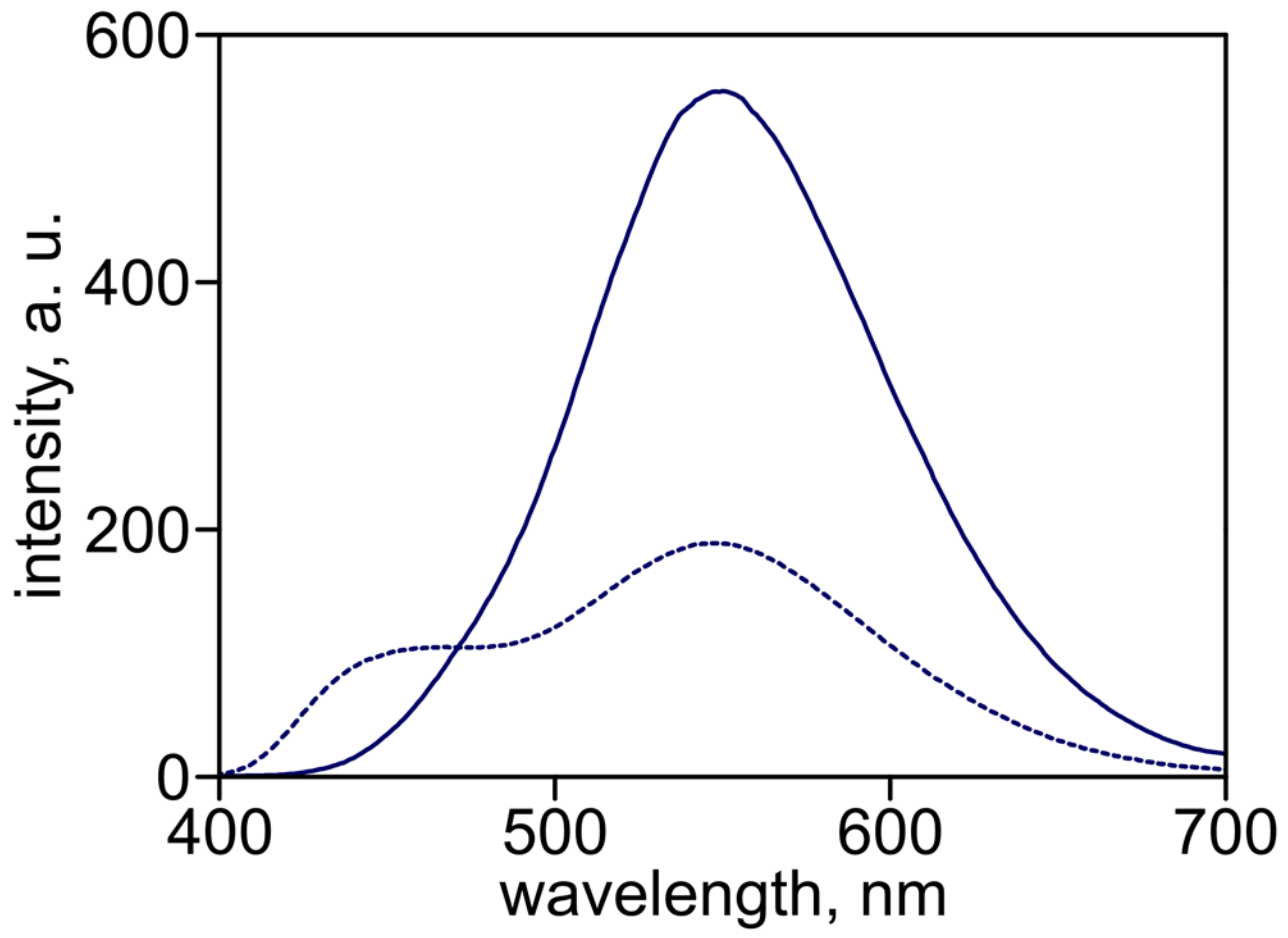

Figure S28. Fluorescence spectra of **rigid L@MIL-53-(OH)$_2$** (solid line) and after soaking in MeOH (dashed line), measured in D_2O (λ_{ex} = 350 nm).

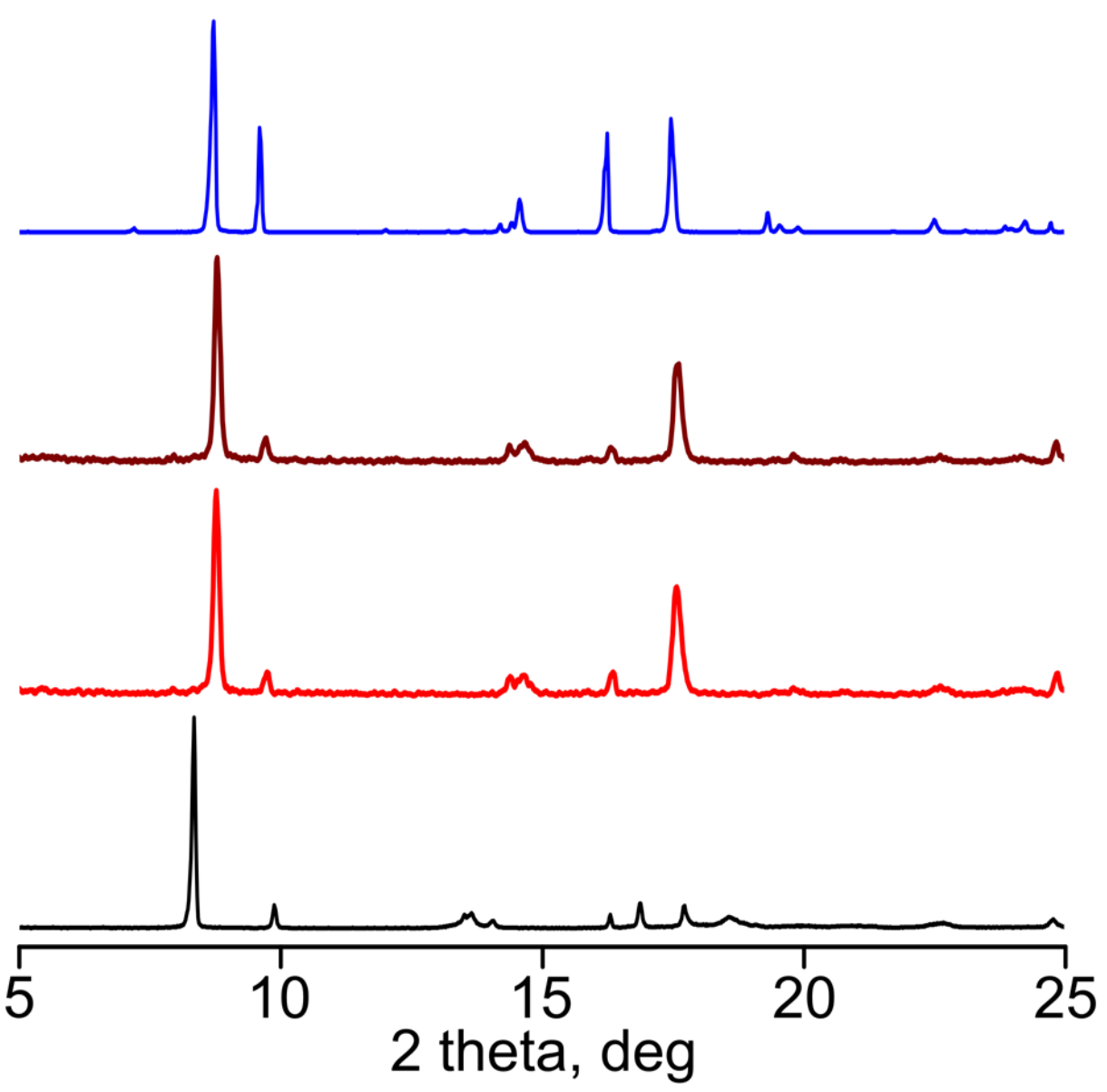

Figure S29. PXRD patterns of post encapsulation of $H_2BDC-NH_2$ to flexible MIL-53-$(OH)_2$ (NH_2@MIL-53-$(OH)_2$): as-synthesized flexible MIL-53-$(OH)_2$ (black), as-synthesized NH_2@MIL-53-$(OH)_2$ (red), air-dried NH_2@MIL-53-$(OH)_2$ (dark red), and rigid L@MIL-53-$(OH)_2$ (blue).

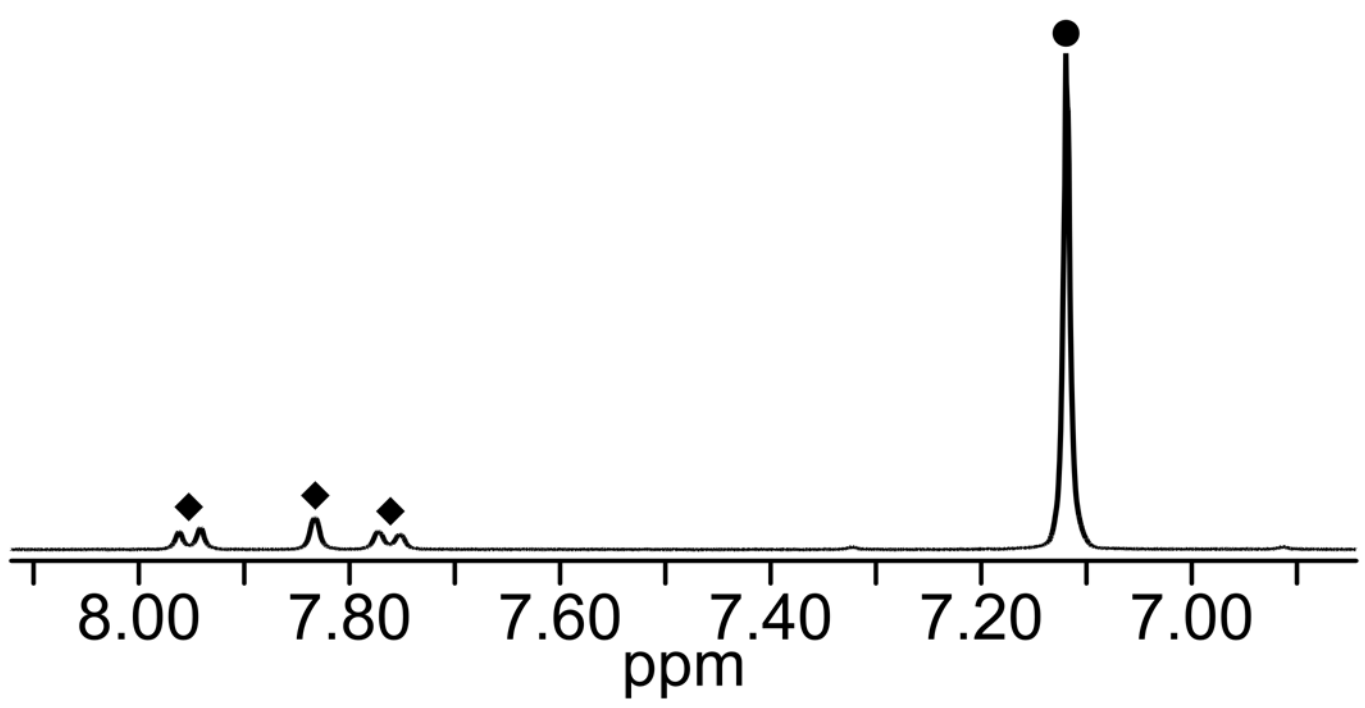

Figure S30. ^{1}H NMR spectrum of after post encapsulation of $H_2BDC-NH_2$ to flexible MIL-53-$(OH)_2$. Circle and squares represent $H_2BDC-(OH)_2$ and $H_2BDC-NH_2$, respectively. The formula is $[In(OH)((BDC)-(OH)_2)]\cdot(H_2BDC-NH_2)_{0.18}$.

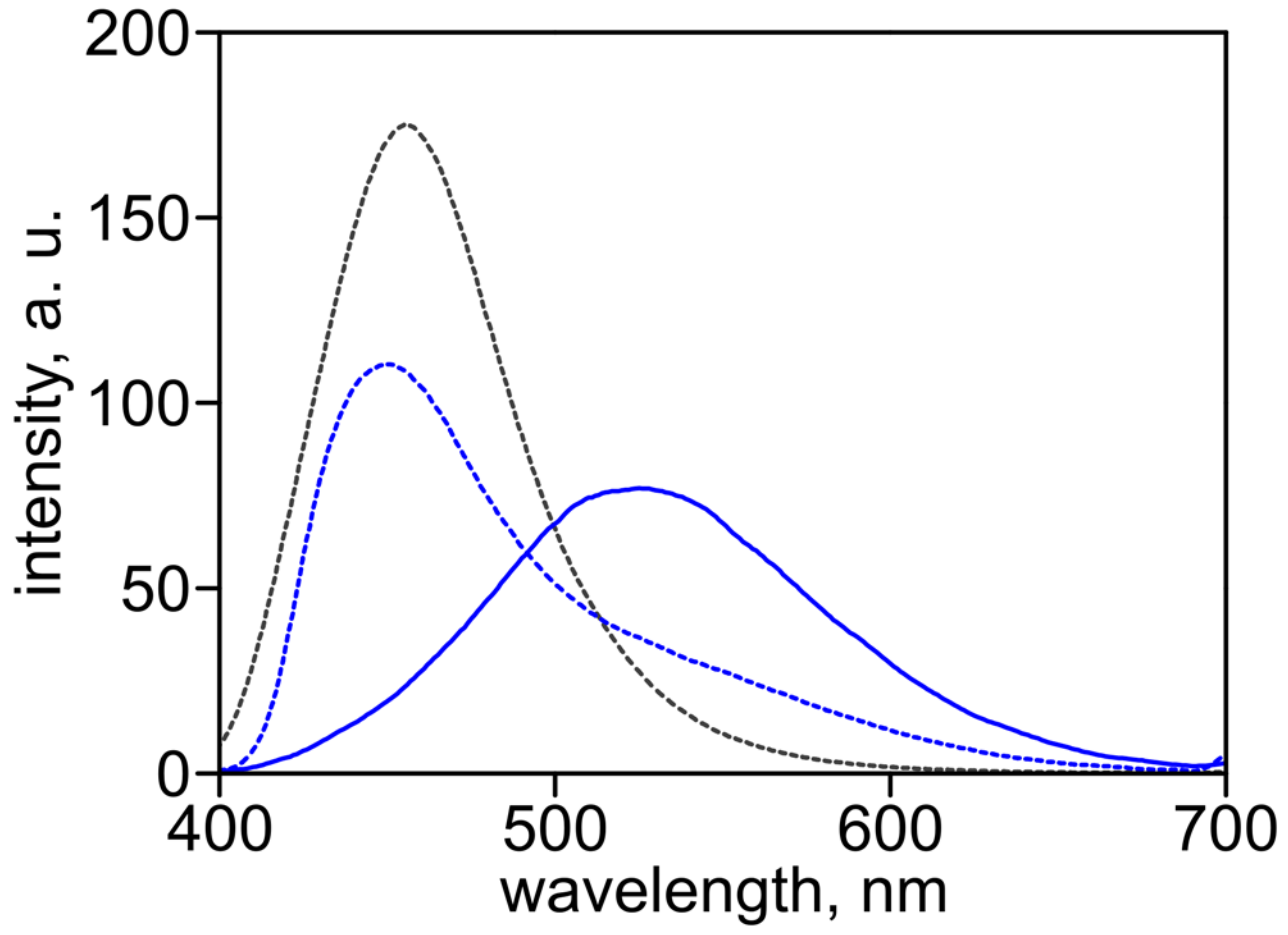

Figure S31. Fluorescence spectra of H_2BDC-NH_2 (gray dashed line), NH_2@MIL-53-$(OH)_2$ (solid blue line), and flexible MIL-53-$(OH)_2$ (dashed blue line) measured in H_2O (λ_{ex} = 350 nm).

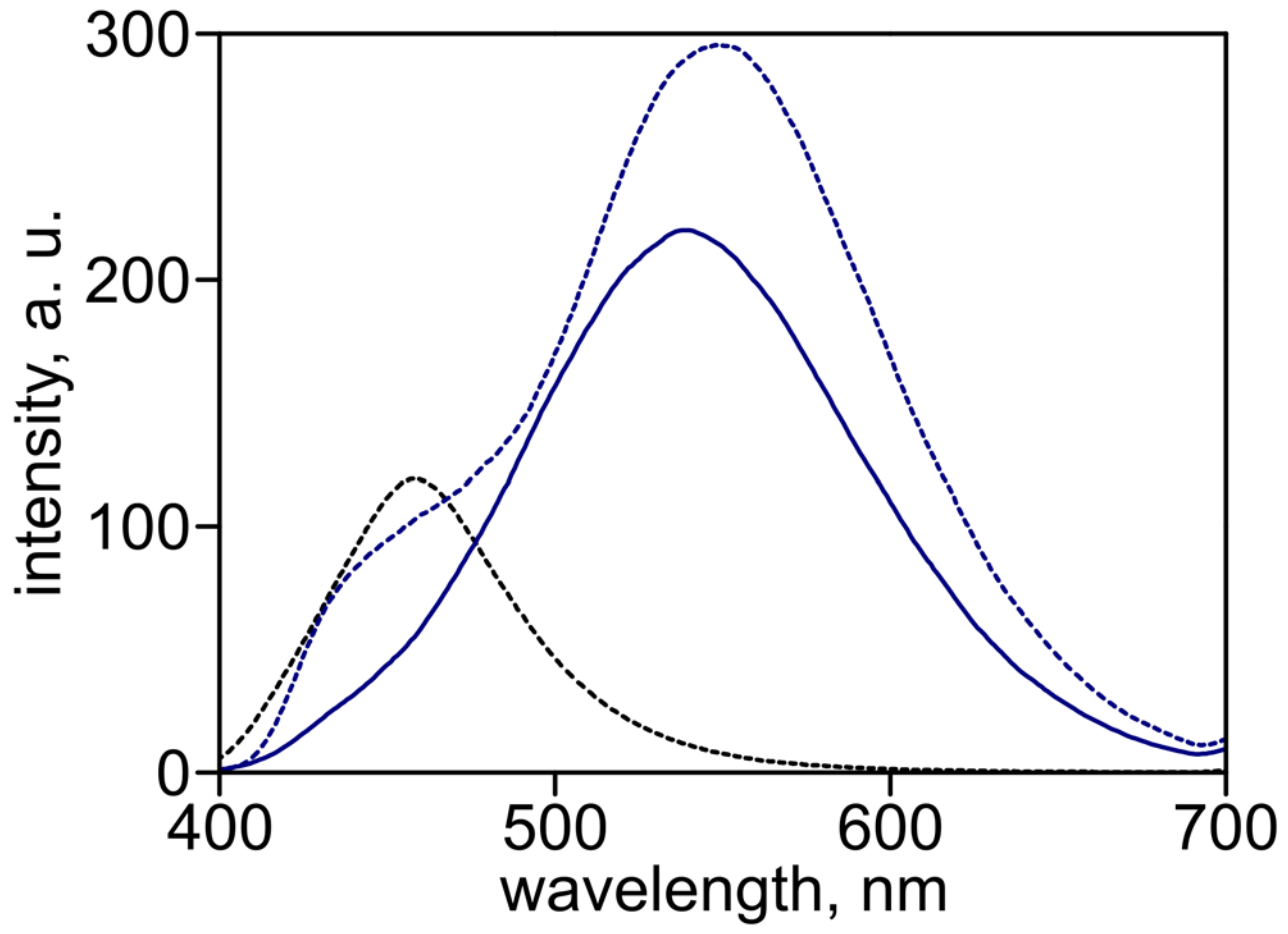

Figure S32. Fluorescence spectra of H_2BDC-NH_2 (black dashed line), NH_2@MIL-53-$(OH)_2$ (solid dark blue line), and flexible MIL-53-$(OH)_2$ (dashed dark blue line) measured in D_2O (λ_{ex} = 350 nm).

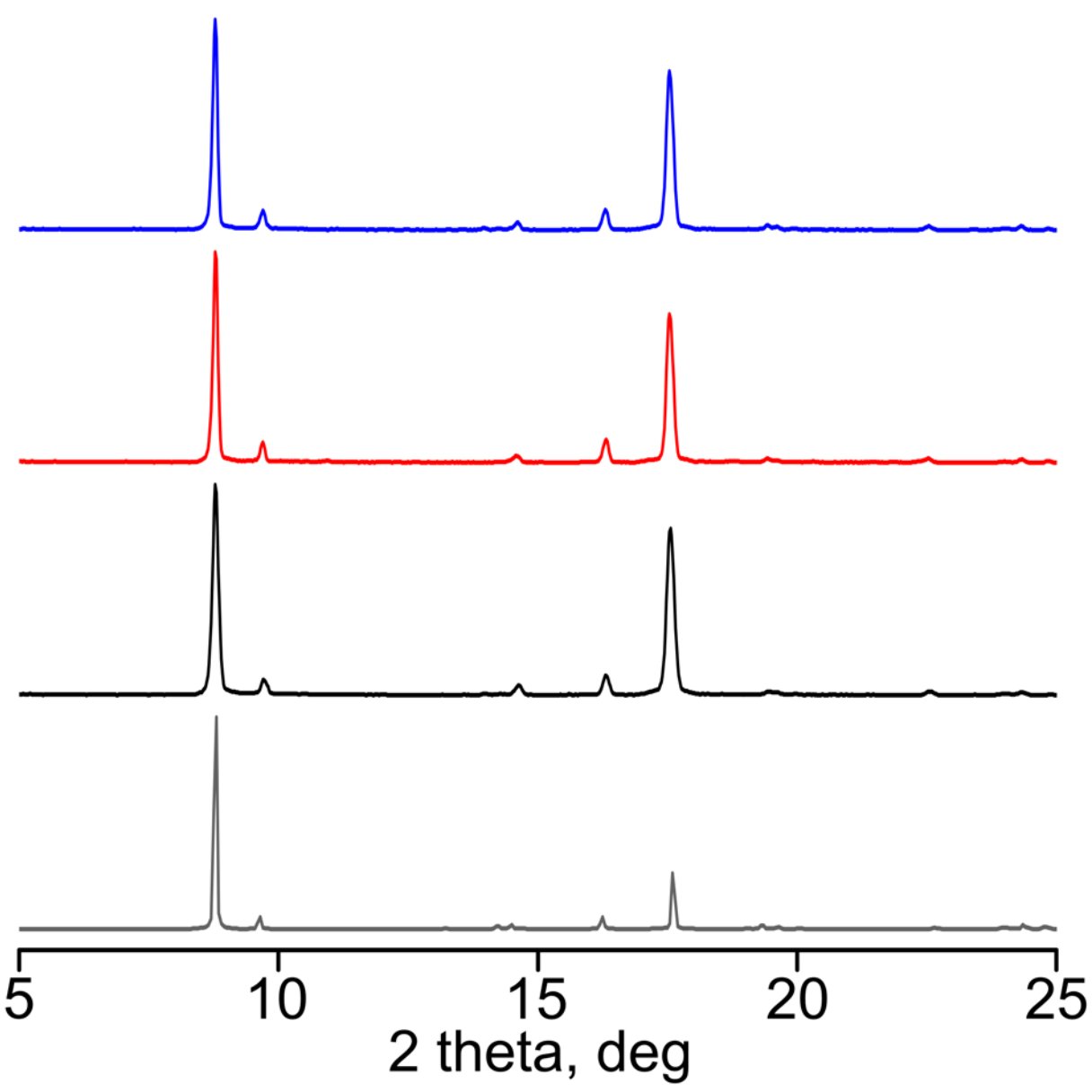

Figure S33. PXRD patterns of **rigid L@MIL-53-(OH)$_2$** evaluated for chemical stability: simulated (gray), as-synthesized (black), pH 1 (red), and pH 10 (blue).

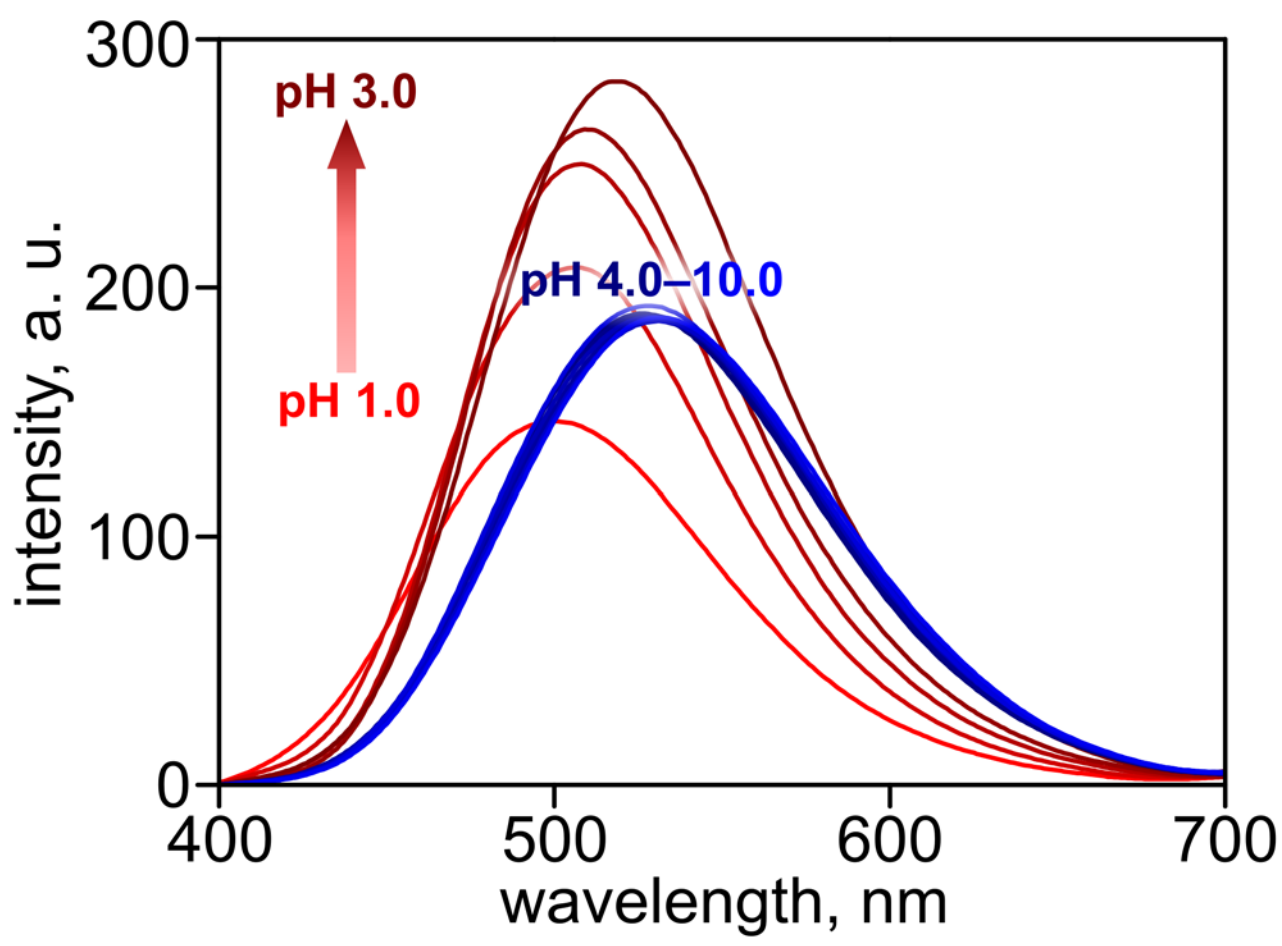

Figure S34. Fluorescence spectra of **rigid L@MIL-53-(OH)$_2$** across a pH range of 1.0 to 10.0 (λ_{ex} = 350 nm). Red to dark red spectra correspond to pH 1.0, 1.5, 2.0, 2.5, and 3.0, while dark blue to blue spectra correspond to pH 4.0, 5.0, 6.0, 7.0, 8.0, 9.0, and 10.0.

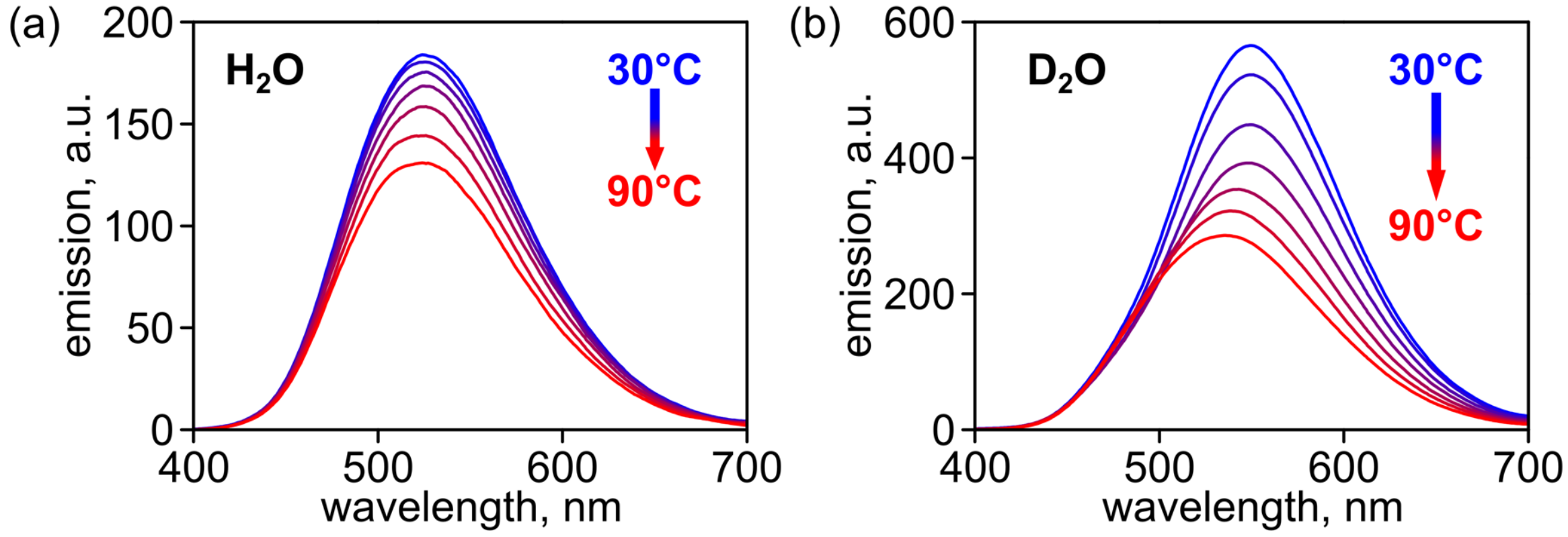

Figure S35. Fluorescence spectra of **rigid L@MIL-53-(OH)$_2$** measured at various temperatures (30–90 °C) in (a) H_2O and (b) D_2O (λ_{ex} = 350 nm).

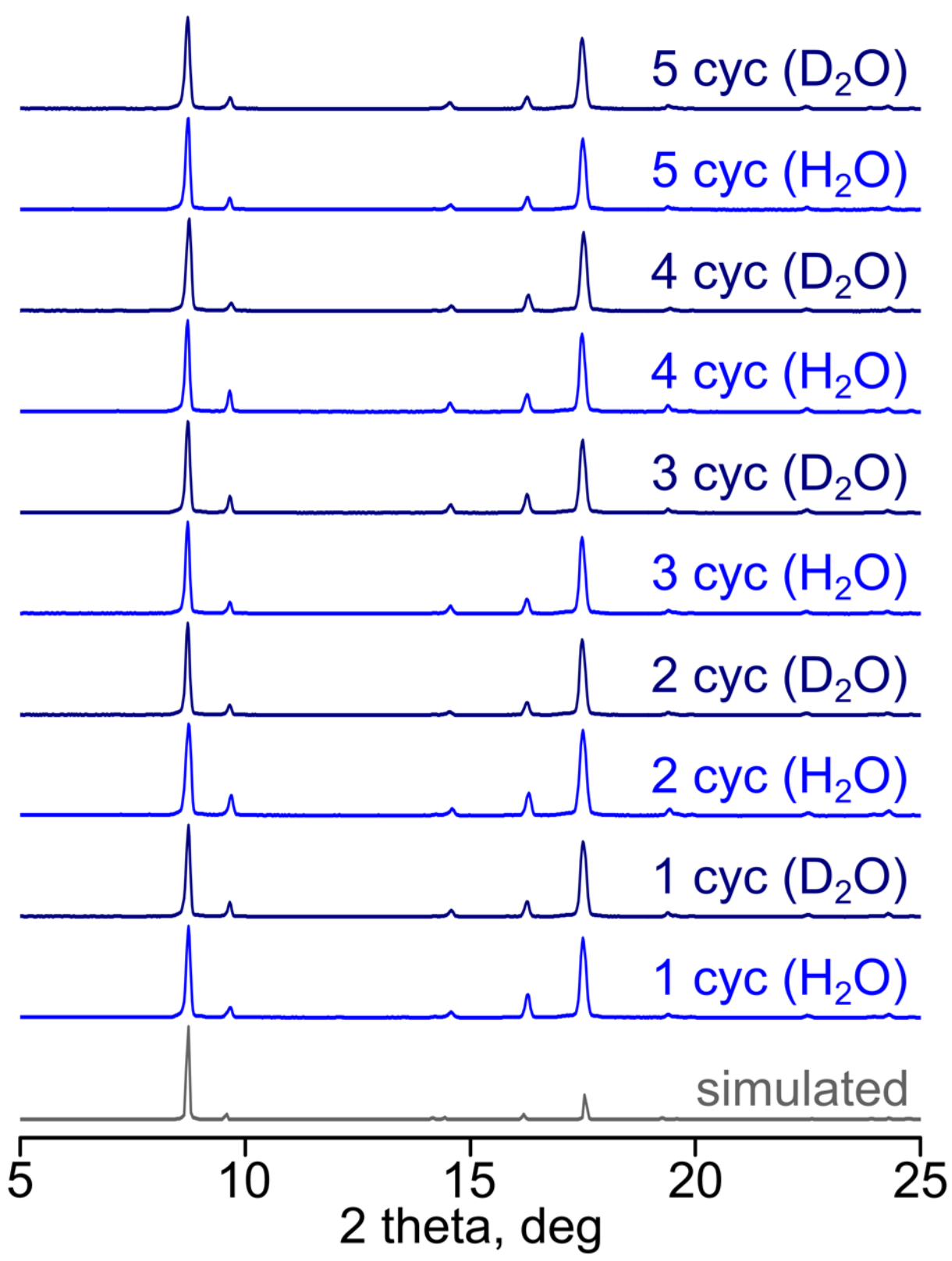

Figure S36. PXRD patterns of **rigid L@MIL-53-(OH)$_2$** after repeated exposure to H2O (blue) and D_2O (dark blue) for up to five cycles, compared with the simulated pattern (gray).

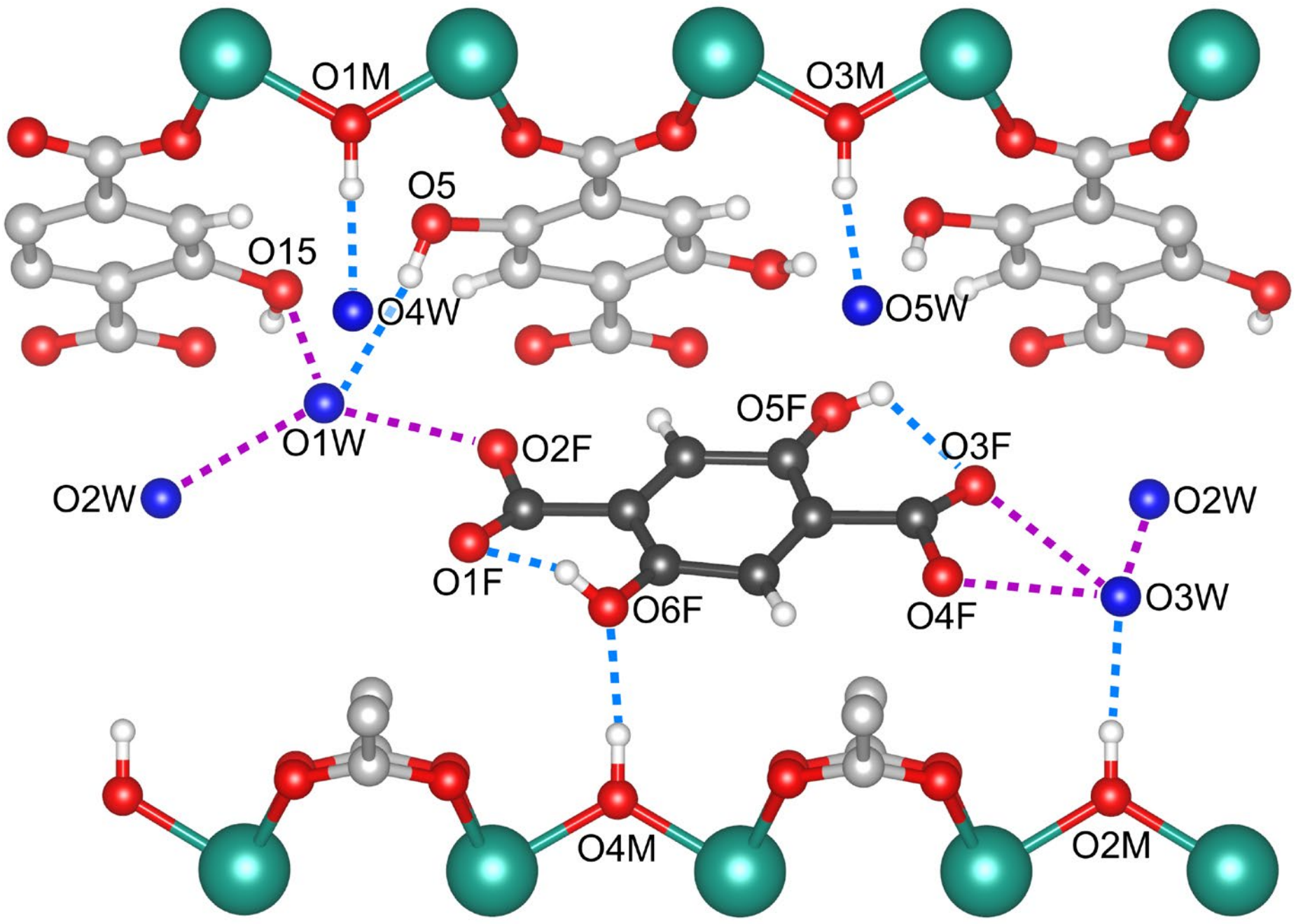

Figure S37. Crystal structure of **rigid L@MIL-53-(OH)$_2$**. White, gray, red, and green spheres represent hydrogen, carbon, oxygen, and indium atoms in the framework, respectively. Dark gray spheres represent carbon atoms in the encapsulated ligand, and blue spheres represent oxygen atoms for structural water. Sky-blue dashed lines represent O–H···O hydrogen bonds, while pink dashed lines indicate potential O···O hydrogen-bonding interactions involving structural water molecules, whose hydrogen atoms were not crystallographically resolved.

References.

[1] A. Sharma, J. Lim, S. Lee, S. Han, J. Seong, S. B. Baek, M. S. Lah, "Superprotonic Conductivity of MOFs Confining Zwitterionic Sulfamic Acid as Proton Source and Conducting Medium," *Angewandte Chemie International Edition* **62**, (2023): e202302376.
[2] A. Sharma, D. Kim, J.-H. Park, S. Rakshit, J. Seong, G. H. Jeong, O.-H. Kwon, M. S. Lah, "Mechanistic insight into the sensing of nitroaromatic compounds by metal-organic frameworks," *Communications Chemistry* **2**, (2019): 39.
[3] J. Perego, C. X. Bezuidenhout, S. Bracco, B.-Q. Song, M. Shivanna, S. Mukherjee, M. J. Zaworotko, A. Comotti, "Coordination-Isomerism-Driven Threshold Pressure Control of CO_2 and C_2H_2 in Mixed-Ligand Switching Metal–Organic Framework," *Journal of the American Chemical Society* **148**, (2026): 8700–8710.
[4] S. Watanabe, S. Hiraide, H. Arima, A. Fukuta, M. Mori, H. Tanaka, M. T. Miyahara, "Size-dependent guest-memory switching of the flexible and robust adsorption characteristics of layered metal-organic frameworks," *Science Advances* **10**, (2024): eadr1387.
[5] A. Chakraborty, S. Ilic, M. Cai, B. J. Gibbons, G. Yang, C. C. Slamowitz, A. Morris, "Role of Spin–Orbit Coupling in Long Range Energy Transfer in Metal–Organic Frameworks," *Journal of the American Chemical Society* **142**, (2020): 20434–20443.
[6] G. R. Wilson, K. C. Park, G. C. Thaggard, C. R. Martin, A. R. Hill, J. Haimerl, J. Lim, B. K. P. Maldeni Kankanamalage, B. J. Yarbrough, K. L. Forrester, R. A. Fischer, P. J. Pellechia, M. D. Smith, S. Garashchuk, N. B. Shustova, "Cooperative and Orthogonal Switching in the Solid State Enabled by Metal-Organic Framework Confinement Leading to a Thermo-Photochromic Platform," *Angewandte Chemie International Edition* **62**, (2023): e202308715.
[7] B. Wang, R. He, Z. J. Lin, X. Zhang, J. Wang, H. Huang, Z. Zhang, K. S. Schanze, J. Zhang, S. Xiang, B. Chen, "Microporous Hydrogen-Bonded Organic Framework for Highly Efficient Turn-Up Fluorescent Sensing of Aniline," *Journal of the American Chemical Society* **142**, (2020): 12478–12485.
[8] D. DeSantis, J. A. Mason, B. D. James, C. Houchins, J. R. Long, M. Veenstra, "Techno-economic Analysis of Metal−Organic Frameworks for Hydrogen and Natural Gas Storage," *Energy Fuels* **31**, (2017): 2024–2032.
[9] A. J. Arvai, C. Nielsen, *ADSC Quantum-210 ADX Program, Area Detector System Corporation*, Poway, CA, USA, (1983).
[10] Z. Otwinowski, W. Minor, "*HKL3000 program," in Methods in Enzymology* (Eds.: Jr. C. W. Carter, R. M. Sweet), Academic Press, New York, 276, (1997): 307.
[11] G. M. Sheldrick, "SHELX program," *Acta Crystallographica Section C: Structural Chemistry* **71**, (2015): 3–8.
[12] K. N. Raymond, G. S. Girolami, "Pathological crystal structures,"*Acta Cryst.* **C79** (2023): 445–455.
[13] A. L. Spek, "PLATON SQUEEZE: a Tool for the Calculation of the Disordered Solvent Contribution to the Calculated Structure Factors," *Acta Crystallographica Section C: Structural Chemistry* **71**, (2015): 9–18.
[14] I. Campeotto, A. Lebedev, A. M. M. Schreurs, L. M. J. Kroon-Batenburg, E. Lowe, S. E. V. Phillips, G. N. Murshudov, A. R. Pearson, "Pathological macromolecular crystallographic data affected by twinning, partial-disorder and exhibiting multiple lattices for testing of data processing and refinement tools," *Sci. Rep.* **8**, (2018): 14876.
[15] S. Liu, A. R. DeFilippo, M. Balasubramanian, Z. Liu, S. G. Wang, Y.-S. Chen, S. Chariton, V. Prakapenka, X. Luo, L. Zhao, J. S. Martin, Y. Lin, Y. Yan, S. K. Ghose, T. A. Tyson, "High-Resolution In-Situ Synchrotron X-Ray Studies of Inorganic Perovskite $CsPbBr_3$: New Symmetry Assignments and Structural Phase Transitions," *Adv. Sci.* **8**, (2021): 2003046.
